# Brief problem-solving therapy for antenatal depressive symptoms in primary care in rural Ethiopia: protocol for a randomised, controlled feasibility trial

**DOI:** 10.1186/s40814-021-00773-8

**Published:** 2021-01-30

**Authors:** Tesera Bitew, Roxanne Keynejad, Bronwyn Myers, Simone Honikman, Girmay Medhin, Fikirte Girma, Louise Howard, Katherine Sorsdahl, Charlotte Hanlon

**Affiliations:** 1 Department of Psychology, Institute of Educational and Behavioural Sciences, Debre Markos University, Debre Markos, Ethiopia; 2 Department of Psychiatry, College of Health Sciences, School of Medicine, Addis Ababa University, Addis Ababa, Ethiopia; 3 Institute of Psychiatry, Psychology & Neuroscience, Section of Women’s Mental Health, King’s College London, London, UK; 4 Alcohol, Tobacco and Other Drug Research Unit, South African Medical Research Council, Cape Town, South Africa; 5 Department of Psychiatry & Mental Health, University of Cape Town, Cape Town, South Africa; 6 Department of Psychiatry and Mental Health, Perinatal Mental Health Project, University of Cape Town, Cape Town, South Africa; 7 Aklilu Lemma Institute of Pathobiology, Addis Ababa University, Addis Ababa, Ethiopia; 8 Department of Psychiatry and Mental Health, Alan J. Fisher Centre for Public Mental Health, University of Cape Town, Cape Town, South Africa; 9 Health Service and Population Research Department, Centre for Global Mental Health, Institute of Psychiatry, Psychology and Neuroscience, King’s College London, London, UK; 10 Centre for Innovative Drug Development and Therapeutic Trials for Africa (CDT-Africa), College of Health Sciences, Addis Ababa University, Addis Ababa, Ethiopia

**Keywords:** Problem solving therapy, Antenatal depression, Psychological interventions, Perinatal mental health; Ethiopia, Low- and middle-income countries

## Abstract

**Background:**

Despite a high prevalence of antenatal depression in low- and middle-income countries, there is very little evidence for contextually adapted psychological interventions delivered in rural African settings. The aims of this study are (1) to examine the feasibility of procedures for a future fully powered efficacy trial of contextually adapted brief problem solving therapy (PST) for antenatal depression in rural Ethiopia, and (2) to investigate the acceptability, fidelity and feasibility of delivery of PST in routine antenatal care.

**Methods:**

Design: A randomised, controlled, feasibility trial and mixed method process evaluation. Participants: Consecutive women attending antenatal clinics in two primary care facilities in rural Ethiopian districts. Eligibility criteria: (1) disabling levels of depressive symptoms (Patient Health Questionnaire (PHQ-9) score of five or more and positive for the 10^th^ disability item); (2) gestational age 12–34 weeks; (3) aged 16 years and above; (4) planning to live in the study area for at least 6 months; (5) no severe medical or psychiatric conditions. Intervention: Four sessions of adapted PST delivered by trained and supervised antenatal care staff over a maximum period of eight weeks. Control: enhanced usual care (EUC). Sample size: *n* = 50. Randomisation: individual randomisation stratified by intimate partner violence (IPV). Allocation: central phone allocation. Outcome assessors and statistician masked to allocation status. Primary feasibility trial outcome: dropout rate. Primary future efficacy trial outcome: change in PHQ-9 score, assessed 9 weeks after recruitment. Secondary outcomes: anxiety symptoms, trauma symptoms, intimate partner violence, disability, healthcare costs at 9 weeks; postnatal outcomes (perinatal and neonatal complications, onset of breast feeding, child health) assessed 4–6 weeks postnatal. Other trial feasibility indicators: recruitment, number and duration of sessions attended. Audio-recording of randomly selected sessions and in-depth interviews with purposively selected participants, healthcare providers and supervisors will be analysed thematically to explore the acceptability and feasibility of the trial procedures and fidelity of the delivery of PST.

**Discussion:**

The findings of the study will be used to inform the design of a fully powered efficacy trial of brief PST for antenatal depression in routine care in rural Ethiopia.

**Trial registration:**

The protocol was registered in the Pan-African clinical trials registry, (PACTR): registration number: PACTR202008712234907 on 18/08/2020; URL: https://pactr.samrc.ac.za/TrialDisplay.aspx?TrialID=9578.

**Supplementary Information:**

The online version contains supplementary material available at 10.1186/s40814-021-00773-8.

## Introduction

Depression in the antenatal period is an important public health challenge [1–3] which makes a substantial contribution to maternal morbidity worldwide [4]. In low- and middle-income countries (LMICs), depression is estimated to adversely affect about 16% of pregnant women [5, 6]. In our previous work in rural Ethiopia, depression during pregnancy was associated with increased use of emergency healthcare [7], increased perinatal complications [8] and unplanned (emergency) institutional delivery [9]. Antenatal depression has been associated with increased functional impairment [2, 10, 11], somatic complaints [2, 12, 13] and reduced self-care [14–16] in the pregnant woman, as well as greater household food insecurity [17], delayed initiation of breast-feeding [18], poor mother-infant attachment, maternal and child undernutrition [18, 19] and poor child health [18].

Improved detection and treatment of antenatal depression in maternal care platforms, particularly within primary healthcare (PHC) settings, has been recommended to reduce maternal morbidity [2, 12, 13], mortality linked to suicide and adverse perinatal outcomes [20]. Although reducing maternal mortality and achieving universal health coverage are key sustainable development goals [21], integration of the detection and treatment of antenatal depression into PHC-based maternal care services has not been prioritised in many LMICs [22]. Antidepressant medication has low acceptability in pregnant women and is only appropriate for moderate-severe depression [23, 24]. However, levels of depressive symptoms that are associated with functional impairment, but that do not necessarily meet criteria for a diagnosis of major depressive disorder have been shown to have adverse effects on the mother and unborn child [25, 26] and can be treated effectively with psychological treatments [27–30]. The World Health Organization mental health gap (mhGAP) intervention guide, designed for PHC workers in LMICs, recommends several brief, manualised psychological therapies for people with depression: interpersonal psychotherapy, cognitive behavioural therapy, behavioural activation therapy and problem solving therapy (PST) [31]. These recommendations are largely based on findings from systematic reviews and meta-analyses of randomised control trials conducted in high-income countries (HICs) or in urban settings in middle-income countries where there are high levels of literacy [27–30]. These interventions are not widely available in most LMICs, especially in rural areas. Available evidence supports the effectiveness of psychological interventions for ante- and postnatal depression that focuses on social problems, female empowerment and involvement of child and family [27, 28], and are delivered by non-specialist community and healthcare workers [29] or peers [30] within the context of routine maternal and child healthcare.

In Ethiopia, mental healthcare remains highly centralized, with mental health specialists located in urban hospitals while more than 80% of the population lives in rural areas. Furthermore, specialist mental healthcare focuses on severe mental illness, with little or no attention given to antenatal depression. As a consequence, there is a large treatment gap (96%) for perinatal depression in Ethiopia [32]. Efforts to expand mental health care in Ethiopia are underway, through integration in PHC and maternal care platforms [33], but is hampered by the lack of therapeutic options for depressed pregnant women. Culturally and contextually relevant and scalable psychological interventions are needed to address this gap. In response to this need, we adapted problem solving therapy (PST) for a rural Ethiopian setting using the Medical Research Council framework for development and evaluation of complex interventions [34] and the ‘ADAPT-ITT’ approach [35].

PST focuses on improving a person’s ability to cope with problems and stressful life experiences [36]. In meta-analyses, PST has been found to be effective for treatment of depression in comparison to no treatment [37, 38]. However, there is limited evidence for PST for antenatal depression in rural, low-income country settings. PST was considered a good fit with the problem-oriented nature of presentations of psychological distress in antenatal care [39], the association of antenatal depression with maladaptive problem-solving and poor coping [40] and because of its simplicity, acceptability and effectiveness in a low literacy population [37, 41–43]. In our qualitative formative work [44], pregnant and postpartum women and community healthcare workers perceived depressive symptoms as responses to situational challenges which may thus be amenable to psychological interventions such as PST.

## Objectives

In the proposed study, we aim to (1) examine the feasibility of trial procedures for a future randomised controlled trial of contextually-adapted brief PST for antenatal depression in rural Ethiopia, and (2) investigate the acceptability, fidelity and feasibility of delivery of PST in routine antenatal care settings.

## Methods

### Setting

The study will be conducted in purposively selected primary healthcare facilities in Sodo and Kibet districts of the Southern Nations, Nationalities and Peoples’ Region (SNNPR). The primary healthcare facilities, primary hospitals and health centres, are staffed by nurses, midwives and health officers. Each health centre is linked to about five health posts, community-based health facilities staffed by health extension workers (HEWs). HEWs are community-based healthcare workers who provide the first antenatal contact, before referring women for further ANC at a health centre or primary hospital, and maintain contact with women during pregnancy.

The study sites are located 100–150 km south of Addis Ababa, the capital city of Ethiopia. Kibet district is in the Silte Zone. Sodo district is one of 15 districts in the Gurage zone of SNNPR. Sodo is where the UK Department for International Development-funded Programme for Improving Mental Health carE (PRIME) [45] worked with local leaders and stakeholders to develop and implement a mental health care plan based on task-shared mental health care delivered in primary and maternal health care settings. The official language of the region and in both districts is Amharic.

### Study design

**Fig. 1:**
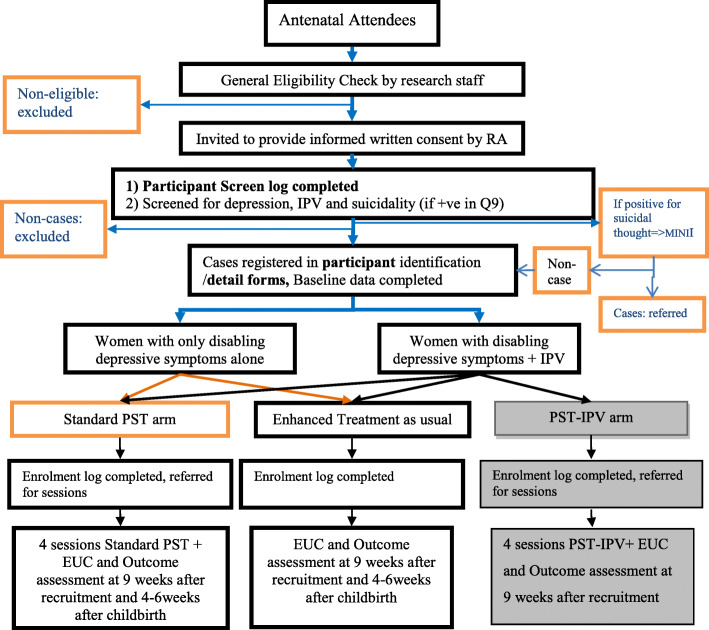
Participant recruitment procedures. Grey boxes indicate nested trial (Keynejad et al. 2020)

The proposed study is a randomised, controlled feasibility trial with two parallel groups and three time point assessments: at baseline, nine weeks after recruitment and 4–6 weeks postnatal (Fig. 1). A mixed qualitative and quantitative methods process evaluation will be nested within the trial. A second feasibility trial involving a sub-sample of trial participants who report past-year exposure to intimate partner violence will be nested within the trial; detailed in the published trial protocol [46].

### Randomised, controlled feasibility trial

The randomised, controlled feasibility trial will compare the adapted PST with enhanced usual care (EUC). Eligibility criteria, participant recruitment procedures, sample size and randomisation procedures, intervention delivery, assessment and data analysis methods will be described in the following sections.

#### Eligibility criteria

##### Inclusion criteria:

Women can be included if they (1) endorse elevated and disabling depression symptoms (scoring 5 or more on the locally validated Patient Health Questionnaire (PHQ-9) [47] and endorse impaired functioning on the 10^th^ PHQ-9 item); (2) are between 12 and 34 weeks gestation; (3) are aged 16 years and above, as this is the age at which married adolescent women become legally autonomous in Ethiopia; (4) are planning to live in the study area for at least 6 months; and (5) speak Amharic (the official regional language).

##### Exclusion criteria:

Women will be excluded if they (1) present with acute medical illness or evidence of severe mental illness; or (2) other comorbid medical conditions such as hypertension or renal disease or diabetes; (3) endorse the 9^th^ item of the PHQ-9 indicating risk of suicide and scoring more than 17 on the Mini International Neuropsychiatric Interview (MINI) [48]; (4) require emergency treatment; or [5] have a condition that impairs their capacity to understand the interview (e.g. diagnosed with severe intellectual disability or dementia).

#### Participant recruitment and screening

Consecutive women attending primary health care-based antenatal care in two primary care facilities will be the target sample for recruitment into the study (Fig. 1) after the research staff obtains informed consent. The research staff will check initial eligibility of antenatal attendees based on information available from the clinical records (gestational age, age, co-morbid conditions and residence). Eligible women will be invited to provide informed consent to an initial screen for depressive symptoms associated with functional impairment using the PHQ-9 and the PHQ-9 disability item. The disability item asks ‘Over the last two weeks, how difficult have these problems made it for you to do your work, take care of things at home, or get along with other people?’ The response options are: ‘Not difficult at all’ = 0, ‘somewhat difficult’ = 1, ‘very difficult’ = 2 or ‘extremely difficult’ = 3. The MINI will be administered to women who endorse any frequency of suicidality on the ninth item of PHQ-9. Women with risk of suicide (MINI≥17) [48] will be excluded from the study and referred for review by a mental health-trained primary care worker [31] who will employ mhGAP [49] to assess risk of suicide. The research staff will facilitate women’s attendance of services at the referral sites and their transportation costs to access care.

Any woman who is found to be eligible following the initial screen will receive written and verbal information about the study from research staff and invited to participate. Women who provide informed consent to participate in the trial will receive a fully structured baseline interview after receipt of their routine clinical care.

Participants’ costs incurred to attend research interviews and PST sessions will be reimbursed. Time spent during research interviews will be compensated, but time spent attending PST sessions will not be compensated to avoid incentivising engagement with the intervention. The healthcare workers will be compensated for their additional effort in the trial.

#### Sample size

Fifty participants (25 in the intervention and 25 in the enhanced usual care group) will be recruited for this feasibility trial. This sample size will enable us to detect a dropout rate of 7% with 95% confidence interval and a 5% margin of error [50]. A sample size of 24–50 is recommended for feasibility studies [51].

#### Randomisation, allocation concealment and masking

Randomisation will be stratified by women’s report of exposure to intimate partner violence, in order to accommodate the nested feasibility trial [46]. Women with disabling antenatal depressive symptoms but no exposure to IPV will be randomised to one of two arms (the PST intervention or enhanced usual care) using simple randomisation in a 1:1 ratio. Women with both disabling antenatal depression and intimate partner violence will be randomised to one of three arms: the PST intervention arm, enhanced usual care arm and a third arm of PST adapted for women exposed to intimate partner violence. Analysis of this third arm’s results will be confined to the nested study (Fig. 1).

Central telephone randomisation will be used to conceal allocation of the participants. A data manager in the Centre for Innovative Drug Development and Therapeutic Trials for Africa (CDT-Africa), external to the study, will allocate each participant to a study arm (PST, PST adapted for women exposed to IPV or enhanced usual care) using a random number list generated by a statistician independent of the research team working in the field (GM). Separate members of research staff based in Sodo/Silte will telephone the data manager to arrange allocation of each new participant. Whenever a new participant has been allocated to that intervention, research staff will inform a clinician trained to deliver the relevant arm. Intervention and control groups will be anonymously coded so that the data analysts remain masked during analysis. Participating women and healthcare providers will not be masked to allocation status due to the nature of the intervention; however, outcome assessors will be masked to allocation status.

#### PST intervention

PST is a commonly used psychotherapy [38, 52] that has comparable efficacy to other psychotherapies such as behavioural activation, cognitive behavioural therapy and social skills training and medication [53, 54]. The PST approach assumes that depressive symptoms are the negative consequences of maladaptive coping in response to problems [55]. The intervention applies principles of evidence-based problem-solving therapy [55] to improve a person’s problem solving and coping skills. In this approach, there are three phases. In the first phase, women are helped to identify the most important things in their life. In the second phase, they identify a list of problems that challenge attainment of important goals in their life and classify the problems into three categories: ‘Problems that are upsetting but not relevant to the most important things in one’s life’ (group A); ‘Problems that are important but, insoluble’ (group B); and ‘important and soluble problems’ (group C). Any psychological therapy should be adapted to the cultural setting to ensure effectiveness, acceptability and feasibility [56]. PST has been contextually adapted and manualised for pregnant women with antenatal depressive symptoms in rural Ethiopia (Bitew 2020, unpublished). The adaptation was based on the Medical Research Council guidelines for developing and evaluating complex interventions [34] and the ADAP-ITT approach [35]. This comprised a series of theory of change workshops, adaptation workshops and in-depth interviews with women with a history of perinatal depression and healthcare providers, and discussions after role play (theatre test).

Participants randomised to the intervention arm will receive four individual sessions of locally adapted and manualised PST, along with enhanced usual care, at a time convenient to them in a private room of the PHC facility. Intervals between sessions will range from a minimum of 2 days to a maximum of 2 weeks over a period of 8 weeks ensuring that all sessions are completed during pregnancy. The first session will last for 1 h and the remaining sessions will last for approximately 40 min each.

The general approach to treatment is introduced in the first session, during which the structure of PST is fully explained, the participant identifies the most important priorities in her life and a list of her current problems is established and classified into the three problem categories: group A, group B and group C problems. In each session, at least one ‘problem busting session’ or ‘group C’ problem, important to the participant and that can potentially be solved, will be discussed and the participant will agree a ‘take home activity’ based on that problem.

The second session will recap on the previous session, review the outcome of the first take-home activity, introduce problem solving strategies for problems categorised as ‘group A’ and include one problem busting session for a group C problem. The third session will review the outcome of the second take-home activity, introduce strategies for problems categorised as ‘group B’ and include one or two problem busting sessions for ‘group C’ problems. The final session will recap the third session and include a problem busting session for any problems which the participant wishes to prioritise.

Trained healthcare workers will deliver the intervention under the supervision of an Ethiopian clinical or counselling psychologist (master’s level) or psychiatrist/psychiatric resident trained in psychological therapies. Healthcare worker training will consist of (a) a 5-day classroom-based training course which includes training on counselling and communication skills as well as PST-specific skills, and (2) accelerated delivery of four sessions of PST, using high-intensity supervision and feedback to build competency [57, 58]. Competency will be established using the Enhancing Assessment of Common Therapeutic factors (ENACT) scale [57, 58] administered by trained clinicians. The healthcare worker will be required to score level 3 (‘Done Well’) out of the three levels: (‘Done Well’, ‘Done partially’ and ‘Need improvement’) on each of the competencies in order to participate in the trial. Feedback will be obtained from women receiving the accelerated PST intervention in the form of three structured questions with open-ended responses: (1) What parts of PST did you find helpful? (2) What parts of PST were unhelpful/need to be improved? How should they be improved? and (3) How convenient was it for you to attend PST? How could this be improved? All intervention sessions will be audio-recorded. A random selection of sessions will be assessed by an expert who has been shown to rate reliably with kappa greater than or equal to 0.80 for repeated scoring. A checklist that contains five dimensions of fidelity [59] will be adapted and used to assess the audio records and then to give feedback focussed on intervention fidelity and core competencies in PST and communication skills. The five dimension of fidelity include (1) adherence (extent to which program components such as program content, methods and activities are delivered as prescribed by the model); (2) exposure (number of sessions or contacts, attendance and the frequency and duration of sessions); (3) quality of delivery (provider enthusiasm, interaction style, respectfulness, confidence and ability to respond to questions and communicate clearly); (4) participant responsiveness (participants’ level of interest in the program; perceptions about the relevance and usefulness of a program; and their level of engagement, enthusiasm and willingness to engage in discussion or activities); and (5) program differentiation (degree to which the critical components of a program are distinguishable from each other and from other programs).

Clinical records and consent forms will be kept in the research office for confidentiality. Research staff will use telephone contact to follow up participants who withdraw without reporting to the research office in order to check for unreported adverse events.

#### Enhanced usual care

Participants allocated to the enhanced usual care arm will receive usual antenatal care which focuses on advice about reproductive and family health issues and information about sources of general support. In addition, the Federal Ministry of Health in Ethiopia has prepared evidence-aligned guidelines on how to care for mental health problems in primary health care and maternal care settings [60]. According to the guideline, primary healthcare staff are expected to detect mental health problems and to provide basic mental healthcare (non-specific psychosocial care for all and supervised prescription of antidepressant medication depending on severity) [61]. All healthcare providers participating in the trial will have been trained in the World Health Organisation’s mhGAP for a minimum of 5 days, as per the mental health scale-up plans of the Federal Ministry of Health. As mhGAP has not been implemented at scale in Ethiopia, this represents an enhancement in usual care [62, 63].

Participants allocated to enhanced usual care and their healthcare providers will be informed about the results of screening and will receive an information sheet and sources of general psychosocial support. These women will attend three contacts: one pre-intervention assessment and two follow-up assessments at 9 weeks after recruitment and 4–6 weeks after childbirth.

#### Assessment of trial participant outcomes

A list of assessment variables and details about their measurement is described in Table 1. Assessment will be conducted at three time points: pre-intervention (T0; baseline), 9 weeks after recruitment (T1; first follow-up) and 4–6 weeks after delivery (T2; second follow-up).

**Table 1:**
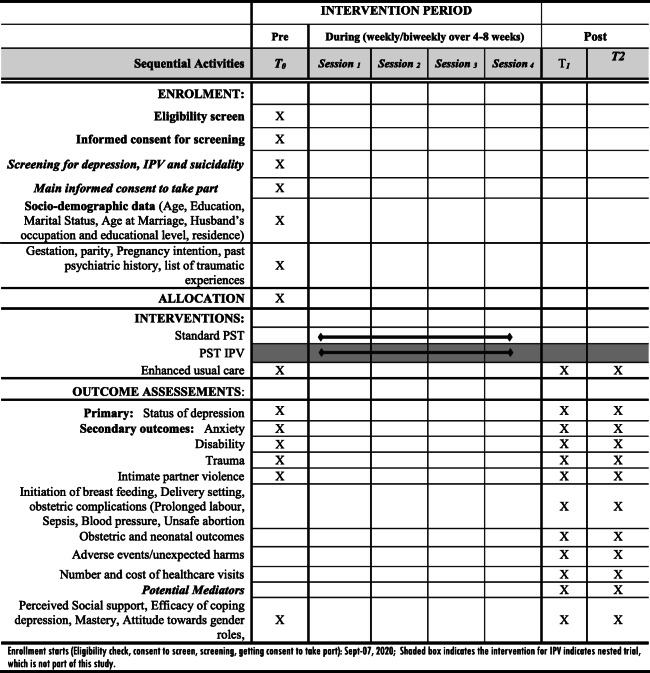
Schedule of enrolment, interventions and assessments

The primary outcome for a future efficacy trial will be change in PHQ-9 score using the locally validated Amharic version of PHQ-9 [64], assessed at T0, T1 and T2. The PHQ-9 has been widely used as a clinical outcome measure of treatment for depression [65, 66]. In Ethiopia, PHQ-9 has been validated in antenatal women [67] and in primary care settings in the neighbouring district of the current study, with the optimal cut-off point indicating probable depression identified as five or more in primary care attendees [47]. At that cut-off, PHQ-9 had a sensitivity of 83.3% and specificity of 74.7% for detection of major depressive disorder. In previous studies, a 50% reduction in PHQ-9 depressive symptom scores [66] after 6–8 weeks was defined as treatment response. A 50% reduction of PHQ-9 score at nine weeks (T1) follow up will be defined as a treatment response for future efficacy trial.

Secondary outcomes to be collected at T0, T1 and T2 are disability (change in WHO disability Assessment Scale, WHODAS, score) [68], anxiety [Generalised Anxiety Disorder-7 scale; GAD-7) [69], PTSD symptoms checklist (PCL-5) [70, 71], intimate partner violence (non-graphic IPV test with five items [72] and WHO multi-country study questions [73]), number of emergency healthcare visits and costs (locally adapted Client Service Receipt Inventory (CSRI) [74]).

At T2, only (4 to 6 weeks postnatal) self-reported information on delivery setting, prolonged labour, sepsis, unsafe abortion, perinatal mortality (stillbirth and neonatal mortality), time of onset of breast-feeding and child health (maternal report of diarrhoea, fever and refusal to breast feed) will be collected.

Potential mediators will be assessed at T0, T1 and T2: attitudes towards gender roles (attitude towards gender roles scale [75]), mastery (Multicultural mastery scale [76]]), self-efficacy of coping with depression (adapted self-efficacy scale [76]) and perceived social support (Oslo Social Support Scale (OSS-3)), previously used in Ethiopia [77] (Table 1).

At T0, a questionnaire will collect data about socio-economic and demographic characteristics of the participants (age, education, marital status, age at marriage, husband’s occupation and educational level, residence and pregnancy intention), obstetric history, including parity, pregnancy intention and gestational age, past psychiatric history and list of traumatic experiences. An item from Ethiopian Demographic Health Survey (EDHS (previously used in our study [7]) will be used to assess pregnancy intention.

### Mixed quantitative and qualitative methods process evaluation

A mixed quantitative and qualitative methods process evaluation will be nested within the feasibility trial. The aims are (1) evaluate the feasibility and acceptability of trial procedures, and (2) to investigate the implementation (feasibility, fidelity and acceptability) of delivery of PST.

#### Participants

Trial participant women, research assistants, supervisors and intervention providers will be included in this mixed study design.

#### Sampling

Women who received the intervention will be purposively selected for semi-structured interview based on their status of completion of the intervention sessions (four sessions of PST). We estimate that about eight participants, both who did and did not complete the PST sessions, will be selected. However, the number of participants will depend on when no new information is obtained. All research assistants, supervisor and providers will be invited to participate in the FGDs. Two separate FGD sessions (one for providers and second for supervisors and research assistants) will be conducted.

#### Process evaluation outcomes

The nested process evaluation investigates two categories of feasibility/implementation outcomes: trial procedure feasibility and acceptability (see Table 2) and implementation of delivery of PST (Table 3). The primary outcome for feasibility trial procedures is dropout rate. Dropout will be defined as the number of women lost to follow up at either of the follow up assessments. Secondary outcomes related to trial procedures and data collection procedures are listed in Table 2. These include participant recruitment rate, feasibility and suitability of eligibility criteria, feasibility of data collection procedures (clarity of baseline questionnaires, characteristics of outcome measures such as missing data, time needed to complete the questionnaires and data collection), adequacy of masking and practical administrative challenges and acceptability of trial procedures (number of refusals to recruitment and screening; qualitative exploration of experience of trial procedures).

**Table 2:** Outcomes of trial procedures

Feasibility outcomes	Data source	Feasibility success	Timing
Feasibility of recruitment			
• Dropouts from the trial/completion of sessions	screening logs, supervision minutes and session records	50% or more women complete sessions	Pre- and during intervention
▪ Feasibility and suitability of eligibility criteria restrictive/too inclusive	Administrative (screening logs) Proceedings of weekly supervision meeting (Agenda: suitability of eligibility)	# of women excluded due to each exclusion criteria	Pre-intervention
Feasibility of data collection procedures			
▪ Clarity of baseline questionnaires (layout, instructions, order of tools))	Proceedings of weekly supervision meeting (Agenda: clarity, time and acceptability of items	20 minutes to complete baseline and follow up battery	Pre-intervention
▪ Characteristics of outcome measures (SD of the outcome and missing data)	Outcome assessment tools	Less than 5% missing data	Post baseline assessment
▪ Time needed to complete questionnaires (reasonable or burden to participants)	Time recorded on questionnaire FGD with supervisors and research assistants	20 minutes to complete baseline and follow up battery	Pre-intervention
• adequacy of masking of assessors and time needed to collect and analyse the data	Administrative documents FGD with supervisors and research assistants		since start of recruitment
▪ Practical administrative and resource challenges to conduct the study (e.g. budget requirements, time, space, staff, skill, intervention materials)	In-depth interviews with participants and healthcare providers, FGD with supervisors and research assistants		Post/ During intervention
Acceptability of trial procedures			
▪ Obstacles to recruitment (recruitment rates, number of eligible patients, number of refusals and screenings)	Administrative data and qualitative data from healthcare providers and participants Proceedings of weekly supervision meeting (Agenda: recruitment challenges)	Less than 10% refusal to be randomized and screened in the flowchart	Pre-intervention
▪ Participant willingness to be randomized (# of women)	Administrative data (enrolment log)	Less than 10% refusal to be randomized	Post/during intervention

**Table 3:** Implementation outcomes for delivery of PST

Implementation outcomes	Data source	Feasibility success	Timing
Feasibility of PST			
▪ Completion of PST activities (homework)	Clinical logs	Completion of homework (80%)	During intervention
▪ Practical administrative and resource challenges to conduct the study (e.g. budget requirements, time, space, staff, skill, intervention materials)	Semi-structured interviews with participants and healthcare providers, FGD with supervisors and research assistants		During intervention
▪ All adverse events, including both expected and unexpected (identification, documentation, referral and reporting)	Administrative data (notes in routine clinical data), participant self-report, identified if the woman drops out of the study.	5% or less report of Adverse Events	During intervention
Fidelity (adherence of therapist to contents, methods and ethical standards)	Audio rating using adherence/fidelity checklist, supervision records	More than 80% adherence	During intervention
Acceptability of intervention delivery			
• Participants’ satisfaction with therapeutic services	Helping alliance questionnaire (HAQ) [78]		After intervention
• Suitability of measures	Proceedings of weekly supervision meeting and FGD with research assistants		During intervention
▪ Provider and service user perception	In-depth interviews (participants and healthcare providers) [79]		After intervention
▪ Relevance of the intervention	In-depth interviews (participants and healthcare providers) [79], session records.		After intervention
▪ Retention and follow up rates, number and duration of sessions attended	Administrative data	50% or more women complete sessions	During intervention
Appropriateness and Sustainability of intervention	In-depth interviews (participants and healthcare providers) [79]		After intervention
Implementation costs	locally adapted Client Service Receipt Inventory (CSRI) [73]) and administrative data		During/post intervention
Implementation strategy (task-shared psychological intervention)	Therapist competence to deliver PST (ENACT scale [56, 57])	90% or more done well (3 points) on each item	

To document the successful implementation of an intervention, the following implementation outcomes are recommended: acceptability, adoption, appropriateness, feasibility, fidelity, implementation cost and sustainability [78] as detailed in Table 3.

#### Assessment

The primary outcome of the feasibility study, dropout from the trial, will be documented as part of routine administrative data. Administrative documents, log books, FGD with research assistants and supervisors and proceedings of supervisory team will be used to document data about these primary and secondary trial procedure outcomes.

Two categories of assessment tools will be employed to assess the PST delivery implementation outcomes: (1) qualitative data (interview, FGD, audio recorded sessions) and (2) assessment tools (outcome assessment tools, Helping Alliance Questionnaire (HAQ), ENACT, fidelity checklist). See Table 3 for an overall summary of the data to be collected to document PST delivery implementation outcomes. Semi-structured interviews will be carried out with research assistants, supervisors and participants who received the intervention and FGDs with providers of the intervention to investigate acceptability and feasibility of the intervention delivery. Semi-structured interviews with women who received the intervention will make use of the same three structured questions with open-ended responses that were used as a competency check during provider training ((1) What parts of PST did you find helpful? (2) What parts of PST were unhelpful/need to be improved? How should they be improved? and (3) How convenient was it for you to attend PST? How could this be improved?). The interview topic guides with participants will focus on any perceived benefits of the intervention, challenges of delivering and receiving the intervention and the strengths and weaknesses of intervention delivery and their experience of the intervention delivery.

Intervention sessions will be audio recorded and some randomly selected participants’ sessions will be assessed. Expert who rated the intervention sessions during provider training will rate the sessions using the checklist described previously that contains five dimensions of fidelity [59]. FGD topic guides for providers, supervisor and research assistants will focus on reflection of their experiences about the intervention, including opportunities and challenges related to intervention delivery in routine settings.

### Data collection procedures and quality

Double data entry will be used for the quantitative data. Range checks will be done for data values. For qualitative data, the data will be collected from multiple sources (supervisors, research assistants, intervention providers, women receiving the intervention) for triangulation. Experienced qualitative data collectors with master’s degree and above will conduct the semi-structured interviews and facilitate the FGDs. Audio records of selected PST sessions, in-depth interviews to elicit women’s feedback about delivery of the intervention and from FGDs will be transcribed verbatim and translated into English.

### Data analysis

#### Trial participant outcomes

Descriptive statistics will be used to summarize trial participant characteristics and future efficacy trial outcomes. The proportion of each group meeting criteria for treatment success (a 50% reduction of PHQ-9 score at T1) and mean change in PHQ-9 score will be described for the two arms. All outcomes (measured at T1 and T2) will be summarized for PST and enhanced usual care arms. For continuous outcomes, the standard deviation will be computed, to inform future sample size calculations for the main study. Standard deviation will be computed from participants for whom outcome measures are available without need of imputations. STAT version 13 will be used to analyse the quantitative data.

#### Process evaluation outcomes

Descriptive statistics will be used to summarize characteristics of participants and the primary outcome. PST delivery implementation outcomes such as extent of missing data, numbers of sessions attended, number of homework activities attempted, rates of initial engagement, providers’ fidelity to the intervention protocol and rates of recruitment will be described using descriptives. The transcripts will be coded for thematic analysis to identify issues related to context, mechanisms and implementation as suggested in key guidance for the systematic conduct of process evaluations of complex interventions [79].

## Discussion

There is a need for rigorous studies to provide high-quality evidence about psychological interventions [56] in low-income countries. The MRC guidance for development and evaluation of complex interventions [34] recommends feasibility studies to optimize potential interventions for future efficacy trials. Where feasibility studies have been conducted, it has been found that this leads to improved intervention and evaluation designs for fully powered trials [80, 81]. We have previously described the process followed to produce a contextually appropriate adaptation of PST for women with antenatal depression in a rural Ethiopian setting with a high burden of social adversity and low literacy. In this paper, we have detailed a proposal for a feasibility trial and process evaluation study in order to test procedures for a future trial and investigate implementation outcomes related to the delivery of the PST intervention in routine antenatal care settings. This is an essential step towards planning a future fully powered randomised controlled trial. This study may be limited by the need to pay health workers to deliver the new intervention because it is not yet a recognised part of their work; this compensation may affect the assessment of future feasibility. Thus, we anticipate a challenge in balancing the need to gain a proof-of-concept by ensuring that the intervention is delivered optimally, against real-world applicability. Our process evaluation may help us to identify potential solutions to overcome challenges in a future efficacy trial. Unforeseen geo-political events and the COVID-19 pandemic may compromise the conduct and completion of this protocol as currently planned. Telephone interviews and use of large rooms while using facemasks would be options to reduce the effects of the pandemic during data collection. Early indications of the high mental health burden associated with the COVID-19 pandemic (Ambaw et al. unpublished data, 2020) and the associated infection control measures mean that this study will remain highly relevant to improving maternal care outcomes.

## Trial status

This is protocol version 1.0. This protocol has been prospectively submitted to the Pan-African clinical trials registry on 13 December 2019. Recruitment of participants is anticipated to commence in October, 2020. Any proposed changes to the protocol will be submitted to Ababa University ethics review boards and updated on the pan-African clinical trials registry. Research staff will inform trial participants, where required, and changes will be discussed in the ultimate results publication. The trial sponsor is Addis Ababa University, Addis Ababa, Ethiopia.

## Supplementary Information

**Additional file 1.**

## Data Availability

Not applicable.

## Abbreviations

ANC: Antenatal care
DSM-IV: Diagnostic statistical manual–IV
FGD: Focus group discussion
HAQ: Helping alliance questionnaire
HEWs: Health extension workers
HICs: High-income countries
LMICs: Low- and middle-income countries
PHC: Primary health care
PHQ-9: Patient health questionnaire-9
PST: Problem solving therapy
RCT: Randomised controlled trial
SNNPR: Southern nations and nationalities and peoples region
WHODAS: World health Organization disability scale

## References

[1] Grote N, Bridge J, Gavin A, Melville J, Iyengar S, Katon W (2010). A meta-analysis of depression during pregnancy and the risk of preterm birth, low birth weight, and intrauterine growth restriction. Arch Gen Psychiatry.

[2] Senturk V, Hanlon C, Medihin G, Dewey M, Araya M, Alem A, Prince M, Stewart R (2012). Impact of Perinatal somatic and common mental disorder symptoms on functioning on Ethiopian Women: The P-MaMiE population based cohort study. J Affect Disord.

[3] Stein A, Pearson R, Goodman S, Rapa E, Rahman A, McCallum M (2014). Effects of perinatal mental disorders on the fetus and child. Lancet.

[4] WHO (2017). Depression and other common mental disorders: global health estimates.

[5] Chowdhary N, Sikander S, Atif N, Singh S, Fuhr D, Rahman A (2014). The content and delivery of psychological interventions for perinatal depression by non-specialist health workers in low and middle income countries: A systematic review. Best Pract Res Clin Obstet Gynaecol.

[6] Fisher J, Mello M, Patel V, Rahman A, Tran T, Holtn S (2012). Prevalence and determinants of common perinatal mentala disorders in low income and lower middle income countries: a systematic review. Bull World Health Organ.

[7] Bitew T, Hanlon C, Kebede E, Medihn G, Fekadu A (2016). Antenatal depressive symptoms and maternal health care utilisation: A population-based study of pregnant women in Ethiopia. BMC Pregnancy Childbirth.

[8] Bitew T, Hanlon C, Kebede E, Honikman S, Fekadu A. Antenatal depressive symptoms and perinatal complication. a prospective study in rural Ethiopia. BMC Psychiatry. 2017;17(301). 10.1186/s12888-017-1462-4 PMC5568236 28830395

[9] Bitew T, Hanlon C, Kebede E, Honikman S, Fekadu A. Effect of ANDs on delivery and postnatal care utilisation: propective studt. BMC Pregnancy Childbirth. 2017;17. 10.1186/s12884-017-1383-8 PMC5492297 28662641

[10] Peltzer K, Szrek H, Ramlagan S, Leite R, Chao L (2015). Depression and social functioning among HIV-infected and uninfected persons in South Africa. AIDS Care..

[11] Bindt C, Appiah-Poku J, Te Bonle M, Schoppen S, Feldt T, Barkmann C (2012). Antepartum depression and anxiety associated with disability in African women: cross-sectional results from the CDS study in Ghana and Cote d'Ivoire. PLoS One.

[12] Hanlon CMG, Alem A, Araya M, Abdulahi A, Tesfaye M, Wondimagegn D, Patel V, Prince M (2008). Detecting perinatal common mental disorders in Ethiopia: validation of self reporting questionaire and Edinburgh postnatal depression scale. J Affect Disord.

[13] Tesfaye M, Hanlon C, Wondimagegn D, Alem A (2009). Detecting Posnatal common mental disorders in Addis Ababa, Ethiopia: Validation of the Edinburgh Postnatal depression scale and Kessler scales. J Affect Disord.

[14] Katon J, Russo J, Gavin A, Melville J, Katon W. Diabetes and depression in pregnancy: is there an association? J Womens Health. 2011;20(7). 10.1089/jwh.2010.2662 PMC3130515 21668382

[15] Katon W (2011). Epidemiology and treatment of depression in patients with chronic medical illness. Dialogues Clin Neurosci.

[16] Katon W, Russo J, Melville J, Katon J, Gavin A (2012). Depression in pregnancy is associated with pre-existing but not pregnancy-induced hypertension. Gen Hosp Psychiatry.

[17] Heyningen T, Myer L, Onah M, Tomlinson M, Field S, Honikman S (2016). Antenatal depression and adversity in urban South Africa. J Affect Disord.

[18] Medihin G, Hanlon C, Dewey M, Alem A, Tesfaye F, Lakew Z (2010). The effect of maternal common mental disorders on infant undernutrition in Butajira, Ethiopia: P-MaMiE study. BMC Psychiatry.

[19] Saeed A, Raana T, Saeed AM, Humayun A (2016). Effect of antenatal depression on maternal dietary intake and neonatal outcome: a prospective cohort. Nutr J.

[20] Kamo T (2015). Perinatal depression: The meaning of the paradigm shift from "postnatal" to "perinatal". Seishin Shinkeigaku Zasshi.

[21] Bárcena A. The 2030 Agenda and the sustainable development goals an opportunity for Latin America and the Caribbean. Sandiago: United Nations; UN.

[22] Baron E, Hanlon C, Mall S, Honikman S, Breuer E, Kathree T (2016). Mental health in primary care in five low and middle income countries: a situtational analysis. BMC Health Serv Res.

[23] Sharma V, Sommerdyk C. Are antidepressants effective in the treatment of postpartum depression? A Systematic Review. Prim Care Comparison CNC Diord. 2013;15(6). 10.4088/PCC.13r01529 PMC3977774 24800125

[24] Kennedy SH, Rizvi S. Comparative efficacy of newer antidepressants for major depression: a Canadian perspective. Can J Diagn. 2009:81–6.

[25] Solomon A, Haaga DA, Arnow BA (2001). Is clinical depression distinct from subthreshold depressive symptoms? A review of the continuity issue in depression research. J Nerv Ment Dis.

[26] Klein DN. Classification of depressive disorders in the DSM-V: proposal for a two-dimension system. J Abnormal Psychol. 2008 2008/08//;117(3):552-560. PubMed PMID: 18729608. eng. 10.1037/0021-843X.117.3.552 PMC3057920 18729608

[27] Patel V. et al., Improving access to psychological treatments: Lessons from developing countries. Behav Res Ther. 2011:1–6. 10.1016/j.brat.2011.06.012. 10.1016/j.brat.2011.06.012 PMC3242164 21788012

[28] Munodawafa M, Mall S, Lund L, Schneider M. Process evaluations of task sharing interventions for perinatal depression in low and middle income countries (LMIC): a systematic review and qualitative meta-synthesis. BMC Health Serv Res. 2018;18. 10.1186/s12913-018-3030-0 PMC5865346 29566680

[29] Rahman A, Fisher F, Bower P, Luchters S, Tran T, Yasamy T (2013). Interventions for common perinatal mental disorders in women in low- and middle-income countries: a systematic review and meta-analysis. Bull World Health Organ.

[30] Singla D, Kohrt B, Murray L, Anand A, Chorpita B, Patel V (2017). Psychological treatments for the world: lessons from low- and middle-income countries: Annual Review of Clinical Psychology. Annu Rev Clin Psychol.

[31] DoMHaS A, WHO (2016). mhGAP intervention guide mental health gap action programme for mental, neurological and substance use disorders in non-specialized health settings.

[32] Azale T, Fekadu A, Hanlon C. Treatment gap and help-seeking for postpartum depression in a rural African setting. BMC Psychiatry. 2016;16:196. 10.1186/s12888-016-0892-8 PMC4901434 27287387

[33] FMOH. National Mental Health Strategy of Ethiopia (2012/13 -2015/16), Addis Ababa. 2012.

[34] Craig P, Dieppe P, Macintyre S, Michie S, Nazareth I, Petticrew M, Council MR (2008). Developing and evaluating complex interventions: new guidance: new guideline.

[35] Wingood G, DiClemente R. The ADAPT-ITT model: a novel method of adapting evidence-based HIV interventions. J Acquir Immune Defic Syndr. 2008;47(supplement 1). 10.1097/QAI.0b013e3181605df1 18301133

[36] Sorsdahl K, Stein D, Carrara H, Myers B (2014). Problem solving styles among peoplewho use alcohol and other drugs in South Africa. Addict Behav.

[37] Bell A, D'Zurilla T (2009). Problem-solving therapy for depression: a meta-analysis☆. Clin Psychol Rev.

[38] Malouff J, Thorsteinsson E, Schutte N (2007). The efficacy of problem solving therapy in reducing mental and physical health problems: a meta-analysis. Clin Psychol Rev.

[39] Azale T, Fekadu A, Hanlon C. Postpartum depressive symptoms in the context of high social adversity and reproductive health threats: a population-based study. Int J Ment Health Syst. 2018 2018/07/28;12(1):42. 10.1186/s13033-018-0219-x PMC6064119 30069229

[40] Azale T, Fekadu A, Medhin G, Hanlon C. Coping strategies of women with postpartum depression symptoms in rural Ethiopia: a cross-sectional community study. BMC Psychiatry. 2018 2018//;18. 10.1186/s12888-018-1624-z PMC5806287 29422037

[41] Chibanda D, Mesu P, Kajawu L, Cowan F, Araya R, Abas M (2011). Problem-solving therapy for depression and common mental disorders in Zimbabwe: piloting a task-shifting primary mental health care intervention in a population with a high prevalence of people living with HIV. BMC Public Health.

[42] Areán P, Raue P, McCulloch C, Kanellopoulos D, Seirup J, Banerjee S (2015). Effects of problem-solving therapy and clinical case management on disability in low-income older adults. Am J Geriatr Psychiatry.

[43] Hof E, Stein D, Marks I, Tomlinson M, Cuijpers P (2011). The effectiveness of problem solving therapy in deprived South African communities: results from a pilot study. BMC Psychiatry.

[44] Bitew T, Keynejad R, Honikman S, Sorsdahl K, Myers B, Abebaw Fekadu A, et al. Stakeholder perspectives on antenatal depression and the potential for psychological intervention in rural Ethiopia: a qualitative study. BMC Pregnancy Childbirth. 2020;20(371). 10.1186/s12884-020-03069-6 PMC7310345 32571246

[45] Lund CTM, De Silva M, Fekadu A, Shidhaye R, Jordans M (2012). PRIME: A Programme to Reduce the Treatment Gap for Mental Disorders in Five Low- and Middle-Income Countries. PLoS Med.

[46] Keynejad R, Bitew T, Sorsdahl K, Myers B, Honikman S, Medhin G, et al. Problem solving therapy (PST) tailored for intimate partner violence (IPV) versus standard PST and enhanced usual care for pregnant women experiencing IPV in rural Ethiopia: protocol for a randomised controlled feasibility trial. Trials. 2020;21:454. 10.1186/s13063-020-04331-0 PMC7268746 32487250

[47] Hanlon C, Medhin G (2015). c, Selamu M, Breuer E., Worku B, Hailemariam H, et al. Validity of brief screening questionnaires to detect depression in primary care in Ethiopia. J Affect Disord.

[48] Roaldset J, Linaker O, Bjørkly S (2012). Predictive validity of the MINI suicidal scale for self-harm in acute psychiatry: a prospective study of the first year after discharge. Arch Suicide Res.

[49] Fekadu A, Hanlon C, Medhin G, Alem A, Selamu M, Giorgis TW, et al. Development of a scalable mental healthcare plan for a rural district in Ethiopia. Br J Psychiatry. 2016;208(Suppl 56):4–12. 10.1192/bjp.bp.114.153676 PMC4698551 26447174

[50] Viechtbauer W, Smits L, Kotz D, Budé L, Spigt M, Serroyen J (2015). A simple formula for the calculation of sample size in pilot studies. J Clin Epidemiol.

[51] Abbas Tavallaii S, Ebrahimnia M, Shamspour N, Assari S (2009). Effect of depression on health care utilization in patients with end-stage renal disease treated with hemodialysis. Eur J Intern Med.

[52] Zhang A, Park S, Sullivan JE, Jing S (2018). The effectiveness of problem-solving therapy for primary care Patients' depressive and/or anxiety disorders: a systematic review and meta-analysis. J Am Board Fam Med.

[53] Bell AC, D'Zurilla TJ (2009). Problem-solving therapy for depression: a meta-analysis. Clin Psychol Rev..

[54] Kirkham JG, Choi N, Seitz DP (2016). Meta-analysis of problem solving therapy for the treatment of major depressive disorder in older adults. Int J Geriatr Psychiatry.

[55] Oxman T, et al. Problem-Solving Treatment and Coping Styles in Primary Care Minor Depression. J Consult Clin Psychol. 2008;76(6): 933–43. 10.1037/a0012617 PMC2593861 19045962

[56] Chowdhary N, Jotheeswaran A, Nadkarni A, Hollon S, King M, Jordans M (2014). The methods and outcomes of cultural adaptations of psychological treatments for depressive disorders: a systematic review. Psychologl Med.

[57] Kohrt B, Jordans M, Rai S, Shrestha P, Luitel N, Ramaiuya M (2015). Therapist competence in global mental health: development of the enhancing assessment of common therapeutic factors (ENACT) scale. Bhavr Res Ter.

[58] Kohrt B, Ramaiya M, Bhardwaj A, Jordans M (2015). Development of a scoring systeme for non-specialits rating of clincial competernce in global mental health: a qualtiative process evaluation of the enhancing assessment of common therapeutic factors (ENACT) scale. Global Mental Health.

[59] JBA. Evaluation brief: Measuring implementation fidelity. In: Associates JB, editor. Arlington, VA: Author.2009.

[60] Feyissa Y, Hanlon C, Emyu S, Cornick R, Fairall L, Gebremichael D (2019). Using a mentorship model to localise the Practical Approach to Care Kit (PACK): from South Africa to Ethiopia. BMJ Global Health.

[61] Fdro E, FMoH (2017). Ethiopia primary healthc are clinical juidelines: care of children 5-14 years and adulte 15 years or older in health centeres.

[62] Gureje O, Oladeji BD, Araya R, et al. Expanding care for perinatal women with depression (EXPONATE): study protocol for a randomized controlled trial of an intervention package for perinatal depression in primary care. BMC Psychiatry. 2015;15(136). 10.1186/s12888-015-0537-3 PMC4486135 26122982

[63] Gureje O, Oladeji B, Montgomery A, Araya R, Bello T, Chisholm D, et al. High- versus low-intensity interventions for perinatal depression delivered by non-specialist primary maternal care providers in Nigeria: cluster randomised controlled trial (the EXPONATE trial). British J Psychiatry. 2019:1–8. 10.1192/bjp.2019.4 30767826

[64] Kroenke K, Spitzer R, Wiliams J (2001). Validity of a brief depression severity measure. J Gen Intern Med..

[65] Dejusu R, Vickers K, Melin G, Williams M (2007). A system-based approach to depression management in primary care using the patient health questionnaire-9. Mayo Clin Proc.

[66] Mclntyre R, Fallu A, Konarski J. Measurable Outcomes in Psychiatric Disorders: Remission as a Marker of Wellness. Clin Ther. 2006;28(11). 10.1016/j.clinthera.2006.11.007 17213009

[67] Girma F (2013). Detecting depression during pregnancy: validation of PHQ-9, Kessler-10, Kessler-6 and SRQ-20 in Butajira area health centers antenatal care clinics, Ethiopia. Addis Ababa University, Ethiopia.

[68] WHO. Measuring health and disability manual for WHO disability assessment schedule: WHODAS 2.0. 2010.

[69] Spitzer R, Kroenke K, Williams J (2006). al e. A brief measure for assessing generalized anxiety disorder The GAD-7. Arch Intern Med.

[70] Yohannes K, Gebeyehu A, Adera T, Ayano G, Fekadu W (2018). Prevalence and correlates of post-traumatic stress disorder among survivors of road traffic accidents in Ethiopia. Int J Ment Health Syst.

[71] Blevins C, Weathers F, Davis M, Witte T, Domino J (2015). The Posttraumatic stress disorder checklist for DSM-5 (PCL-5): development and initial psychometric evaluation. J Trauma Stress..

[72] Zink T, Levin L, Putnam F, Beckstrom A (2007). Accuracy of five domestic violence screening questions with nongraphic language. Clin Pediatr.

[73] Garcia-Moreno C, Jansen HA, Ellsberg M, Heise L, Watts CH. Prevalence of intimate partner violence: findings from the WHO multi-country study on women's health and domestic violence. Lancet. 2006;368(9543):1260–1269. 10.1016/S0140-6736(06)69523-8 17027732

[74] Sorato B. client service reciept inventory 2003.

[75] Fok CCT, Allen J, Henry D, Mohatt GV. Multicultural Mastery Scale for Youth: multidimensional assessment of culturally mediated coping strategies. Psychol Assess. 2012;24(2):313. 10.1037/a0025505 PMC3394699 21928912

[76] Fok CCTAJ, Henry D, Mohatt GV (2012). Multicultural Mastery Scale for Youth: Multidimensional assessment of culturally mediated coping strategies. Psychol Assess.

[77] Berg L, Beutel M, Hinz A, Zenger M, Härter M, Nater U, Brähler E (2018). Social support in the general population: Standardization of the Oslo social support scale (OSSS-3). BMC Psychol.

[78] Proctor ESH, Raghavan R, Hovmand P, Aarons G, Bunger A (2011). Outcomes for implementation research: conceptual distinctions, measurement challenges, and research agenda. Adm Policy Ment Health Ment Health Serv Res.

[79] Moore GF, Audrey S, Barker M, Bond L, Bonell C, Hardeman W (2015). Process evaluation of complex interventions: Medical Research Council guidance. bmj..

[80] Richards SH, Campbell JL, Dickens C, Anderson R, Gandhi M, Gibson A (2018). Enhanced psychological care in cardiac rehabilitation services for patients with new-onset depression: the CADENCE feasibility study and pilot RCT. Health Technol Assess.

[81] Richards SH, Campbell JL, Dickens C, Anderson R, Gandhi M, Gibson A (2016). Assessing the effectiveness of enhanced psychological care for patients with depressive symptoms attending cardiacrehabilitation compared with treatment as usual (CADENCE): study protocol for a pilot cluster randomised controlled trial. Trials.

