# Supplementary material for: Brief problem-solving therapy for antenatal depressive symptoms in primary care in rural Ethiopia: protocol for a randomised, controlled feasibility trial
Source: Pilot Feasibility Stud. 2021 Jan 30;7:35. doi: 10.1186/s40814-021-00773-8 (PMC7846490; doi:10.1186/s40814-021-00773-8)
Supplement: Supplementary file 1 — Additional file 1. [file 40814_2021_773_MOESM1_ESM.docx]

# Suplementary Files

# Suplementary Files 1: Information Sheet for Participants

*IRB Reference Number:*

**YOU WILL BE GIVEN A COPY OF THIS INFORMATION SHEET**

Hello! My name is _______________________________________ and I am working with Addis Ababa University. We are conducting a research on maternal health care utilization in Sodo District, Guragie Zone, Ethiopia. We would like to invite you to participate in this original research project. You don't have to be in the survey, but we hope you will agree to answer the questions since your views are important. If I ask you any question you don't want to answer, just let me know and I will go on to the next question or you can stop the interview at any time.

You should only participate if you want to; choosing not to take part will not disadvantage you in any way. Before you decide whether you want to take part, it is important for you to understand why the research is being done and what your participation will involve. Please take time to read the following information carefully and discuss it with others if you wish. Ask us if there is anything that is not clear or if you would like more information.

We are going to ask you information about your maternity health care utilization and your experience in relation to pregnancy and delivery. In addition to our current interview, we will also ask you similar questions after your delivery. The information we collect will help planning health services. All of the answers you give will be confidential and will not be shared with anyone other than members of our survey team.

- **Aims of the research**

This study is primarily looking at the the potential barriers of maternal health care utilization during pregnancy, delivery and after birth. It tries to look at the relation between maternal mental health problems and its effect on maternal health care utilization as well as adverse obstetric outcomes.

- **Who are we recruiting?**

We are including pregnant women with consent who are at least in the second trimester and permanently residing in the study area. We will also be interviewing health workers and key members of the community like Traditional Birth Attendants, who have better awareness about the maternal health care utilization issue.

- **What will happen if you agree to take part?**

One of our data collectors will either come to your place of work or your home and ask you some questions. The questions will be asking about your own experience of maternal health care utilization and obstetric complications during pregnancy, delivery and postpartum and your own perrception health care utilization and obstetric commplications. The interview will take about one hour.

- **Risks of being in the study**

We don’t expect that the interview will cause you any difficulties. On rare occasions, people might be upset by the questions that are being asked. If you are distressed by the questions then you do not have to answer the question and the interview can be stopped. But, also note that the information is secured and be accessable aonly our survey team.

- **Possible benefits**

We hope that the information obtained will help to improve the quality and quantity of maternal health care services like antenatal care services, delivery care services and postpartum care services. We hope that it will fundamentally benefit to improve the health care of the mothers and the newborn. Once the study is completed, we will let you know what we found, either by inviting you to a meeting or giving you a leaflet.

- **What we will do with your data**

The questionnaires will not include your name so nobody except the project co-ordinators (Ato Tesera Bitew) and project data managers will know the information that belongs to you.

We will keep the questionnaires in a locked cupboard.

If you take part in the tape-recorded interview, we will make sure that the tapes do not include your name or identifying information. The tapes will be kept in a locked cupboard. Once the interview tapes have been written down, and the data has been analysed, the tapes will be cleared.

After the end of this study, the information you tell us may be used by other researchers, but they will not be able to identify you in any way.

**Main researcher:** You can contact the main researcher for any other detailed information using the following address at working hours.

- Ato Tesera Bitew, PhD candidate in Addis Ababa University, Department of Psychiatry
- Tel number 0911 17 36 56

It is up to you to decide whether to take part or not. If you decide to take part you are still free to withdraw at any time and without giving a reason.

If this study has harmed you in any way you can contact the Institutional Review Board, Addis Ababa University, using the details below for further advice and information:

Institutional Review Board, School of Medicine, Addis Ababa University

Telephone number: 0115-5538734

- You may withdraw your data from the project at any time up until it is transcribed for use in the final report.
- If you do decide to take part you will be given this information sheet to keep and be asked to sign a consent form.

#
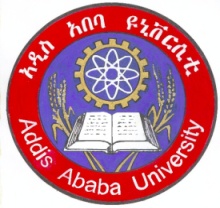
 Suplementary Files 2: Consent Form (English version)

Please complete this form after you have read the Information Sheet and/or listened to an explanation about the research.

**Title of Study: Brief problem-solving therapy for antenatal depressive symptoms in primary care in rural Ethiopia: protocol for a feasibility trial**

**Addis Ababa University Research Ethics Committee Ref:** .................................................

*Thank you for considering taking part in this research. The person organizing the research must explain the project to you before you agree to take part. If you have any questions arising from the Information Sheet or explanation already given to you, please ask the researcher before you decide whether to join in. You will be given a copy of this Consent Form to keep and refer to at any time.*

- *I understand that if I decide at any time during the research that I no longer wish to participate, or for my child to participate, in this project, I can notify the researchers involved and withdraw from it immediately without giving any reason. Furthermore, I understand that I will be able to withdraw my data up until they are published.*
- *I consent to the processing of my personal information for the purposes explained to me. I understand that such information will be handled in accordance with the terms of the national data protection rules.*
- *If I am selected to be interviewed in more detail then, I consent to that interview being audio-taped.*
- *The information you have submitted will be published as a report. Please note that confidentiality and anonymity will be maintained and it will not be possible to identify you from any publications.*
- *I agree that the research team may use anonymized data for future research.*

***Participant’s Statement:***

**I ­­­­­­­­­­­­_____________________________,** *agree that the research project named above has been explained to me to my satisfaction and I agree to take part in the study. I have read both the notes written above and the Information Sheet about the project, and understand what the research study involves***.**

*Signed ______________________ Date _______________________*

***Witness Statement (in event that participant is not literate)****:*

*I ­­­­­­­­­­­­______________________________, agree that the research project named above has been explained to __________________ (participant) to her satisfaction and that she agrees to take part in the study. Both the notes written above and the Information Sheet about the project have been read to her, and she understands what the research study involves.*

*Signed _______________________ Date _______________________*

***Investigator’s Statement:***

*I, _________________________, confirm that I have carefully explained the nature, demands and any foreseeable risks (where applicable) of the proposed research to the participant.*

*Signed _______________________ Date _______________________*

##

# Suplementary Files 3: Instruments

## 1.1. General Information

### 1.1.1. Socio-demographic Information

| 101 | How old are you now? | /🖎_____/ years | AGE |
| --- | --- | --- | --- |
| 102 | how old when you were first married? | 🖎 /_____/ Years | AGEM |
| 103 | How old were you at your 1st pregnancy in completed years? | 🖎 /_____/ Years | AGEPR |
| 104 | Respondent's **residence**? [1] Rural [0] Urban | | RESID |
| 105 | What is your Marital Status?  [1] Single [2] Married [3] Divorced  [4] widowed [5] Living with a partner [6] Married but living apart | | MARIT |
| 106 | What is your **religion**?  [1] Orthodox Christian [2] Muslim [3] Protestant  [4] Catholic [9] Other | | RELIG |

### 1.1.2. Socio-economic Information

| 108 | | **Education** (What is your level of education)?  [1] Illiterate [2] Literate but no formal education  [3] Grade 1-4 [4] Grade 5-8  [5] Grade 9-10 [6] Grade 11-12  [7] Certificate [8] Diploma  [9] BA/BSc [10] MA/MSc and above | EDUCM | | | |
| --- | --- | --- | --- | --- | --- | --- |
| 109 | | What is your main **occupation**?  [1] House wife [2] Daily Laborer [3] Farmer  [4] Employee [5] Student [6] Trader/Merchant  [7] unemployed [8] Pensioned [97] other [specify ___________] | OCCM | | | |
| 111 | **Relative wealth** (Compared to others in the Kebele, how do you see your economic Standing?) [1] Poor [2] Average [3] Better off | | | WELZR | |  |
| 114 | How many hectares of farm land do you have? 🖎_______________ hectares | | | MASA | |  |
| 115 | | **Husband’s Occupation (**What is the main occupation of your husband?)  [1] unemployed [2] Daily Laborer [3] Farmer  [4] Trader/Merchant [5] Student [5] Employee [77] Other | | | OCCH | |
| 116 | | **Education** (What is your husband’s level of education)?  [1] Illiterate [2] Literate but no formal education  [3] Grade 1-4 [4] Grade 5-8  [5] Grade 9-10 [6] Grade 11-12  [7] Certificate [8] Diploma  [9] BA/BSc [10] MA/MSc and above | | | EDUCH | |

## 1.2. Patient Health Questionnaire (PHQ-9)

Over the past 2 weeks, have you been bothered by any of the following problems?

| 1 | Little interest or pleasure in doing things?^[[1]](#footnote-1)^ | | Yes^[[2]](#footnote-2)^ | | If yes, how frequently in the last 2 weeks?^[[3]](#footnote-3)^ | | | Several days | 1 |
| --- | --- | --- | --- | --- | --- | --- | --- | --- | --- |
|  |  |  | No | |  |  |  | More than half the days | 2 |
|  |  |  |  |  |  |  |  | Nearly everyday | 3 |
| 2 | Feeling down, depressed or hopeless? | | Yes | | If yes, how frequently in the last 2 weeks? | | | Several days | 1 |
|  |  |  | No | |  |  |  | More than half the days | 2 |
|  |  |  |  |  |  |  |  | Nearly everyday | 3 |
| 3A^[[4]](#footnote-4)^ | Trouble falling or staying asleep? | | Yes | | If yes, how frequently in the last 2 weeks? | | | Several days | 0.5 |
|  |  |  | No | |  |  |  | More than half the days | 1 |
|  |  |  |  |  |  |  |  | Nearly everyday | 1.5 |
| 3B | Sleeping too much? | | Yes | | If yes, how frequently in the last 2 weeks? | | | Several days | 0.5 |
|  |  |  | No | |  |  |  | More than half the days | 1 |
|  |  |  |  |  |  |  |  | Nearly everyday | 1.5 |
| 4 | Feeling tired or having little energy? | | Yes | | If yes, how frequently in the last 2 weeks? | | | Several days | 1 |
|  |  |  | No | |  |  |  | More than half the days | 2 |
|  |  |  |  |  |  |  |  | Nearly everyday | 3 |
| 5A | Poor appetite? | Yes | | If yes, how frequently in the last 2 weeks? | | | Several days | | 1 |
|  |  | No | |  |  |  | More than half the days | | 2 |
|  |  |  |  |  |  |  | Nearly everyday | | 3 |
| 5B | Over-eating? | Yes | | If yes, how frequently in the last 2 weeks? | | | Several days | | 1 |
|  |  | No | |  |  |  | More than half the days | | 2 |
|  |  |  |  |  |  |  | Nearly everyday | | 3 |
| 6 | Feeling bad about yourself?  Or that you are a failure?  Or have let yourself or your family down? | Yes | | If yes, how frequently in the last 2 weeks? | | | Several days | | 1 |
|  |  | No | |  |  |  | More than half the days | | 2 |
|  |  |  |  |  |  |  | Nearly everyday | | 3 |
| 7 | Trouble concentrating on things, such as reading the newspaper or watching television? | Yes | | If yes, how frequently in the last 2 weeks? | | | Several days | | 1 |
|  |  | No | |  |  |  | More than half the days | | 2 |
|  |  |  |  |  |  |  | Nearly everyday | | 3 |
| 8A | Moving or speaking so slowly that other people could have noticed? | Yes | | If yes, how frequently in the last 2 weeks? | | | Several days | | 1 |
|  |  | No | |  |  |  | More than half the days | | 2 |
|  |  |  |  |  |  |  | Nearly everyday | | 3 |
| 8B | Being so fidgety or restless that you have been moving around a lot more than usual? | Yes | | If yes, how frequently in the last 2 weeks? | | | Several days | | 1 |
|  |  | No | |  |  |  | More than half the days | | 2 |
|  |  |  |  |  |  |  | Nearly everyday | | 3 |
| 9 | Thought that you would be better off dead or of hurting yourself in some way? | Yes | | If yes, how frequently in the last 2 weeks? | | | Several days | | 1 |
|  |  | No | |  |  |  | More than half the days | | 2 |
|  |  |  |  |  |  |  | Nearly everyday | | 3 |
| [If responded 0 for ALL question PHQ1-PHQ9 🡪 go to next section] | | | | | | | | |  |
| 10 | Over the last two weeks, how difficult have these problems made it for you to do your work, take care of things at home, or get along with other people? | | | | | Not difficult at all | | | 0 |
|  |  |  |  |  |  | Somewhat difficult | | | 1 |
|  |  |  |  |  |  | Very difficult | | | 2 |
|  |  |  |  |  |  | Extremely difficult | | | 3 |

## 1.3. Intimate Partner Violence Measures

### 1.3.1. Non-Graphic Language Screening Questions

No matter how well a couple gets along, there are times when they disagree. Couples get annoyed with the other person, or just have spats or fights because they’re in a bad mood or tired of for some other reason. They also use many different ways of trying to settle their differences. I’m going to ask what you and your partner might feel when you have an argument.

| 1 | How do you and your partner work out arguments? | No difficulty | 0 |  |
| --- | --- | --- | --- | --- |
|  |  | Some difficulty | 1 |  |
|  |  | Great difficulty | 2 |  |
| 2 | In general, how do you describe your relationship? | No tension | 0 |  |
|  |  | Some tension | 1 |  |
|  |  | A lot of tension | 2 |  |
| 3 | How is your partner treating you (and your children)? | Always well | 0 |  |
|  |  | Well most of the time | 1 |  |
|  |  | Neutral | 2 |  |
|  |  | Not well most of the time | 3 |  |
|  |  | Never well | 4 |  |
| 4 | Do you feel safe in your current relationship? | Always safe | 0 |  |
|  |  | Safe most of the time | 1 |  |
|  |  | Neutral | 2 |  |
|  |  | Not safe most of the time | 3 |  |
|  |  | Never safe | 4 |  |
| 5 | Considering your current partners, friends, or any past partners or friends, is there anyone who is making you feel unsafe now? | Always safe | 0 |  |
|  |  | Safe most of the time | 1 |  |
|  |  | Neutral | 2 |  |
|  |  | Not safe most of the time | 3 |  |
|  |  | Never safe | 4 |  |

### 13.2. WHO Multi-country Survey: IPV Questions

When two people marry or live together, they usually share both good and bad moments. I would now like to ask you some questions about your current and past relationships and how your husband/partner treats/treated you. If anyone interrupts us I will change the topic of conversation. I would again like to assure you that your answers will be kept secret, and that you do not have to answer any questions that you do not want to. May I continue?

| 1 | In your relationship with your husband, how often do you argue? | | Rarely | 0 |
| --- | --- | --- | --- | --- |
|  |  |  | Sometimes | 1 |
|  |  |  | Often | 2 |
| 2 | I will now ask you about some situations that are true for many women. Thinking about your husband, is it generally true that he: | (a) Tries to stop you from seeing your friends? | Yes | 1 |
|  |  |  | No | 0 |
|  |  | (b) Tries to stop you contacting your family of birth? | Yes | 1 |
|  |  |  | No | 0 |
|  |  | (c) Insists on knowing where you are all the time? | Yes | 1 |
|  |  |  | No | 0 |
|  |  | (d) Ignores you and treats you indifferently? | Yes | 1 |
|  |  |  | No | 0 |
|  |  | (e) Gets angry if you speak to another man? | Yes | 1 |
|  |  |  | No | 0 |
|  |  | (f) Is often suspicious that you are unfaithful? | Yes | 1 |
|  |  |  | No | 0 |
|  |  | (g) Expects you to ask his permission before seeking health care for yourself? | Yes | 1 |
|  |  |  | No | 0 |

| 3 | The next questions are about things that happen to many women, and that your husband may have done to you. I want you to tell me if your husband or any other partner has ever done the following things to you in the past 12 months. | | | | | |
| --- | --- | --- | --- | --- | --- | --- |
|  | (a) | Insulted you or made you feel bad about yourself? | Yes | If yes, how many times in the past 12 months? | Once | 1 |
|  |  |  | No (scores 0)  Skip to 3(b) |  | A few times | 2 |
|  |  |  |  |  | Many times | 3 |
|  | (b) | Belittled or humiliated you in front of other people? | Yes | If yes, how many times in the past 12 months? | Once | 1 |
|  |  |  | No (scores 0)  Skip to 3(c) |  | A few times | 2 |
|  |  |  |  |  | Many times | 3 |
|  | (c) | Did things to scare or intimidate you on purpose (e.g. by the way he looked at you, by yelling and smashing things)? | Yes | If yes, how many times in the past 12 months? | Once | 1 |
|  |  |  | No (scores 0)  Skip to 3(d) |  | A few times | 2 |
|  |  |  |  |  | Many times | 3 |
|  | (d) | Threatened to hurt you or someone you care about? | Yes | If yes, how many times in the past 12 months? | Once | 2 |
|  |  |  | No (scores 0) |  | A few times | 3 |
|  |  |  |  |  | Many times | 4 |

| 4 | Has he or any other partner ever… | | | | | |
| --- | --- | --- | --- | --- | --- | --- |
|  | (a) | Slapped you or thrown something at you that could hurt you? | Yes | If yes, how many times in the past 12 months? | Once | 1 |
|  |  |  | No (scores 0)  Skip to 4(b) |  | A few times | 2 |
|  |  |  |  |  | Many times | 3 |
|  | (b) | Pushed you or shoved you? | Yes | If yes, how many times in the past 12 months? | Once | 1 |
|  |  |  | No (scores 0)  Skip to 4(c) |  | A few times | 2 |
|  |  |  |  |  | Many times | 3 |
|  | (c) | Hit you with his fist or with something else that could hurt you? | Yes | If yes, how many times in the past 12 months? | Once | 1 |
|  |  |  | No (scores 0)  Skip to 4(d) |  | A few times | 2 |
|  |  |  |  |  | Many times | 3 |
|  | (d) | Kicked you, dragged you or beaten you up? | Yes | If yes, how many times in the past 12 months? | Once | 1 |
|  |  |  | No (scores 0)  Skip to 4(e) |  | A few times | 2 |
|  |  |  |  |  | Many times | 3 |
|  | (e) | Choked or burnt you on purpose? | Yes | If yes, how many times in the past 12 months? | Once | 1 |
|  |  |  | No (scores 0)  Skip to 4(f) |  | A few times | 2 |
|  |  |  |  |  | Many times | 3 |
|  | (e) | Threatened to use or actually used a gun, knife or other weapon against you? | Yes | If yes, how many times in the past 12 months? | Once | 1 |
|  |  |  | No (scores 0) |  | A few times | 2 |
|  |  |  |  |  | Many times | 3 |

| 5 | Has he or any other partner ever… | | | | | |
| --- | --- | --- | --- | --- | --- | --- |
|  | (a) | Physically forced you to have sexual intercourse when you did not want to? | Yes | If yes, how many times in the past 12 months? | Once | 1 |
|  |  |  | No (scores 0)  Skip to 5(b) |  | A few times | 2 |
|  |  |  |  |  | Many times | 3 |
|  | (b) | Did you ever have sexual intercourse you did not want because you were afraid of what he might do? | Yes | If yes, how many times in the past 12 months? | Once | 1 |
|  |  |  | No (scores 0)  Skip to 5(c) |  | A few times | 2 |
|  |  |  |  |  | Many times | 3 |
|  | (c) | Did he ever force you to do something sexual that you found degrading or humiliating? | Yes | If yes, how many times in the past 12 months? | Once | 1 |
|  |  |  | No (scores 0) |  | A few times | 2 |
|  |  |  |  |  | Many times | 3 |

| 6 | Check: did she answer Yes to any question on Physical Violence? | Yes | 1 |
| --- | --- | --- | --- |
|  |  | No | 0 |
| 7 | Check: did she answer Yes to any question on Sexual Violence? | Yes | 1 |
|  |  | No | 0 |

## 1.4. PCL-5


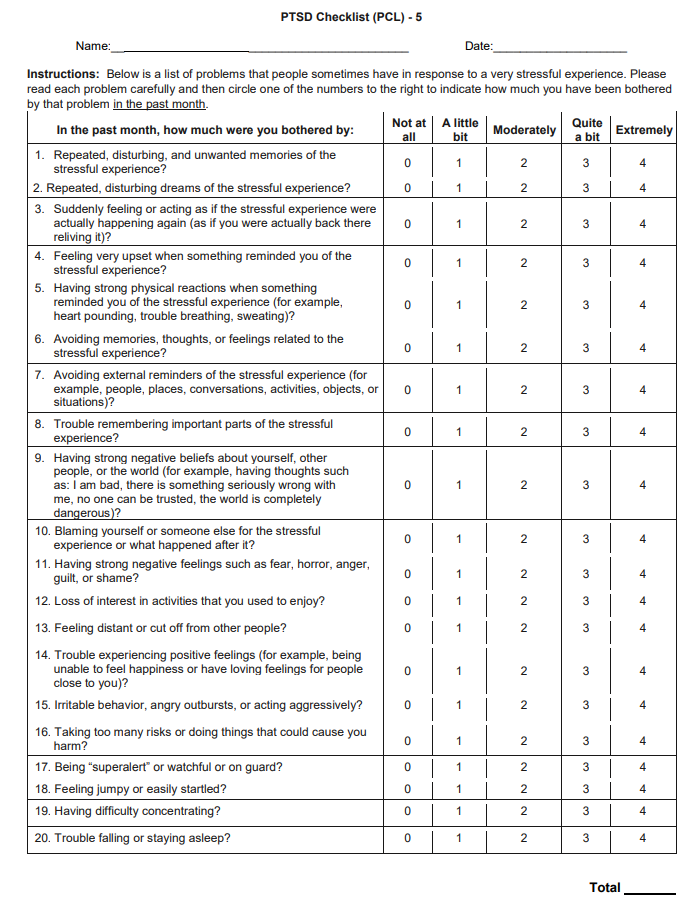


## 1.5. GAD-7

| Over the last 2 weeks, how often have you been bothered by the following problems? | | | | | |
| --- | --- | --- | --- | --- | --- |
| 1 | Feeling nervous, anxious or on edge? | Yes^1^ | If yes, how frequently over the last 2 weeks?^2^ | Several days | 2 |
|  |  | No (scores 1) |  | More than half the days | 3 |
|  |  |  |  | Nearly every day | 4 |
| 2 | Not being able to stop or control worrying? | Yes | If yes, how frequently over the last 2 weeks? | Several days | 2 |
|  |  | No (scores 1) |  | More than half the days | 3 |
|  |  |  |  | Nearly every day | 4 |
| 3 | Worrying too much about different things? | Yes | If yes, how frequently over the last 2 weeks? | Several days | 2 |
|  |  | No (scores 1) |  | More than half the days | 3 |
|  |  |  |  | Nearly every day | 4 |
| 4 | Trouble relaxing? | Yes | If yes, how frequently over the last 2 weeks? | Several days | 2 |
|  |  | No (scores 1) |  | More than half the days | 3 |
|  |  |  |  | Nearly every day | 4 |
| 5 | Being so restless that it is hard to sit still? | Yes | If yes, how frequently over the last 2 weeks? | Several days | 2 |
|  |  | No (scores 1) |  | More than half the days | 3 |
|  |  |  |  | Nearly every day | 4 |
| 6 | Becoming easily annoyed or irritable? | Yes | If yes, how frequently over the last 2 weeks? | Several days | 2 |
|  |  | No (scores 1) |  | More than half the days | 3 |
|  |  |  |  | Nearly every day | 4 |
| 7 | Feeling afraid as if something awful might happen? | Yes | If yes, how frequently over the last 2 weeks? | Several days | 2 |
|  |  | No (scores 1) |  | More than half the days | 3 |
|  |  |  |  | Nearly every day | 4 |

^1^ Response categories were simplified (initial yes/no, with further probing of the frequency in persons responding ‘yes’).

^2^ Reference period is repeated for each item.

## 1.6. World Health Organization Disability Assessment Schedule (WHODAS-12)

| The next few questions are about difficulties people have because of health conditions.  [Hand flashcard to respondent]  By health condition I mean diseases or illnesses, other health problems that may be short or long lasting, injuries, mental or emotional problems and problems with alcohol or drugs.  I remind you to keep all of your health problems in mind as you answer the questions. When I ask you about difficulties in doing an activity think about [Point to flashcard #1].  • Increased effort  • Discomfort or pain  • Slowness  • Changes in the way you do the activity  [Point to flashcard #1]. When answering, I’d like you to think back over the last 30 days. I also would like you to answer these questions thinking about how much difficulty you have, on average over the past 30 days, while doing the activity as you usually do it.  [Hand flashcard #2 to interviewee] Use this scale when responding.  [Read scale aloud]: None, mild, moderate, severe, extreme or cannot do.  [Flashcards #1 and #2 should remain visible to the respondent throughout the interview] | | | | | |
| --- | --- | --- | --- | --- | --- |
| 1 | How do you rate your overall health in the past 30 days? | Very good | 1 | | OVERALL |
|  |  | Good | 2 | |  |
|  |  | Moderate | 3 | |  |
|  |  | Bad | 4 | |  |
|  |  | Very bad | 5 | |  |
| [Show flashcard #2 to participant.] In the last 30 days how much difficulty did you have in: | | | | | |
| 2 | Standing for long periods such as 30 minutes? | None | 1 | | STAND |
|  |  | Mild | 2 | |  |
|  |  | Moderate | 3 | |  |
|  |  | Severe | 4 | |  |
|  |  | Extreme/cannot do | 5 | |  |
| 3 | Taking care of your household responsibilities? | None | 1 | | HOUSE |
|  |  | Mild | 2 | |  |
|  |  | Moderate | 3 | |  |
|  |  | Severe | 4 | |  |
|  |  | Extreme/cannot do | 5 | |  |
| 4 | Learning a new task, for example, learning how to get to a new place? | None | 1 | | LEARN |
|  |  | Mild | 2 | |  |
|  |  | Moderate | 3 | |  |
|  |  | Severe | 4 | |  |
|  |  | Extreme/cannot do | 5 | |  |
| 5 | How much of a problem did you have in joining community activities (for example, festivities, religious or other activities) in the same way as anyone else can? | None | 1 | | JOIN |
|  |  | Mild | 2 | |  |
|  |  | Moderate | 3 | |  |
|  |  | Severe | 4 | |  |
|  |  | Extreme/cannot do | 5 | |  |
| 6 | How much have you been emotionally affected by your health problems? | None | 1 | | EMOTE |
|  |  | Mild | 2 | |  |
|  |  | Moderate | 3 | |  |
|  |  | Severe | 4 | |  |
|  |  | Extreme/cannot do | 5 | |  |
| In the last 30 days, how much difficulty did you have in: | | | | | |
| 7 | Concentrating on doing something for 10 minutes? | None | 1 | | CONC |
|  |  | Mild | 2 | |  |
|  |  | Moderate | 3 | |  |
|  |  | Severe | 4 | |  |
|  |  | Extreme/cannot do | 5 | |  |
| 8 | Walking a long distance such as a kilometre? | None | 1 | | WALK |
|  |  | Mild | 2 | |  |
|  |  | Moderate | 3 | |  |
|  |  | Severe | 4 | |  |
|  |  | Extreme/cannot do | 5 | |  |
| 9 | Washing your whole body? | None | 1 | | WASH |
|  |  | Mild | 2 | |  |
|  |  | Moderate | 3 | |  |
|  |  | Severe | 4 | |  |
|  |  | Extreme/cannot do | 5 | |  |
| 10 | Getting dressed? | None | 1 | | DRESS |
|  |  | Mild | 2 | |  |
|  |  | Moderate | 3 | |  |
|  |  | Severe | 4 | |  |
|  |  | Extreme/cannot do | 5 | |  |
| 11 | Dealing with people you do not know? | None | 1 | | DEAL |
|  |  | Mild | 2 | |  |
|  |  | Moderate | 3 | |  |
|  |  | Severe | 4 | |  |
|  |  | Extreme/cannot do | 5 | |  |
| 12 | Maintaining a friendship? | None | 1 | | FRIEND |
|  |  | Mild | 2 | |  |
|  |  | Moderate | 3 | |  |
|  |  | Severe | 4 | |  |
|  |  | Extreme/cannot do | 5 | |  |
| 13 | Your day to day work? | None | 1 | | DAY |
|  |  | Mild | 2 | |  |
|  |  | Moderate | 3 | |  |
|  |  | Severe | 4 | |  |
|  |  | Extreme/cannot do | 5 | |  |
| 14 | Overall, how much did these difficulties interfere with your life? | None | 1 | | INTERF |
|  |  | Mild | 2 | |  |
|  |  | Moderate | 3 | |  |
|  |  | Severe | 4 | |  |
|  |  | Extreme/cannot do | 5 | |  |
| 15 | Overall, in the past 30 days, how many days were these difficulties present? | __________ days | | DIFFDAYS | |
| 17 | In the past 30 days, for how many days were you totally unable to carry out your usual activities or work because of any health condition? |  __________ days | | UNABLE | |
| 18 | In the past 30 days, not counting the days you were totally unable, for how many days did you cut back or reduce your usual activities or work because of any health condition? |  __________ days | | CUTBACK | |

## 1.7. Attitudes Towards Gender Roles (WHO Multi-Country Study)

In this community and elsewhere, people have different ideas about families and what is acceptable behaviour for men and women in the home. I am going to read you a list of statements, and I would like you to tell me whether you generally agree or disagree with the statement. There are no right or wrong answers.

| 1 | A good wife obeys her husband even if she disagrees | | Agree | 1 |
| --- | --- | --- | --- | --- |
|  |  |  | Disagree | 2 |
|  |  |  | Don’t know | 8 |
| 2 | Family problems should only be discussed with people in the family | | Agree | 1 |
|  |  |  | Disagree | 2 |
|  |  |  | Don’t know | 8 |
| 3 | It is important for a man to show his wife/partner who is the boss | | Agree | 1 |
|  |  |  | Disagree | 2 |
|  |  |  | Don’t know | 8 |
| 4 | A woman should be able to choose her own friends even if her husband disapproves | | Agree | 1 |
|  |  |  | Disagree | 2 |
|  |  |  | Don’t know | 8 |
| 5 | It’s a wife’s obligation to have sex with her husband even if she doesn’t feel like it | | Agree | 1 |
|  |  |  | Disagree | 2 |
|  |  |  | Don’t know | 8 |
| 6 | If a man mistreats his wife, others outside of the family should intervene | | Agree | 1 |
|  |  |  | Disagree | 2 |
|  |  |  | Don’t know | 8 |
| 7 | In your opinion, does a man have a good reason to hit his wife if: | (a) She does not complete her household work to his satisfaction | Agree | 1 |
|  |  |  | Disagree | 2 |
|  |  |  | Don’t know | 8 |
|  |  | (b) She disobeys him | Agree | 1 |
|  |  |  | Disagree | 2 |
|  |  |  | Don’t know | 8 |
|  |  | (c) She refuses to have sexual relations with him | Agree | 1 |
|  |  |  | Disagree | 2 |
|  |  |  | Don’t know | 8 |
|  |  | (d) She asks him whether he has other girlfriends | Agree | 1 |
|  |  |  | Disagree | 2 |
|  |  |  | Don’t know | 8 |
|  |  | (e) He suspects that she is unfaithful | Agree | 1 |
|  |  |  | Disagree | 2 |
|  |  |  | Don’t know | 8 |
|  |  | (f) He finds out that she has been unfaithful | Agree | 1 |
|  |  |  | Disagree | 2 |
|  |  |  | Don’t know | 8 |
| 8 | In your opinion, can a married woman refuse to have sex with her husband if: | (a) She doesn’t want to | Agree | 1 |
|  |  |  | Disagree | 2 |
|  |  |  | Don’t know | 8 |
|  |  | (b) He is drunk | Agree | 1 |
|  |  |  | Disagree | 2 |
|  |  |  | Don’t know | 8 |
|  |  | (c) She is sick | Agree | 1 |
|  |  |  | Disagree | 2 |
|  |  |  | Don’t know | 8 |
|  |  | (d) He mistreats her | Agree | 1 |
|  |  |  | Disagree | 2 |
|  |  |  | Don’t know | 8 |

## 1.8. Adapted self-efficacy scale

Indicate your level of agreement with the following statements:

1. I feel confident in my ability to discuss problems with my partner.

| Strongly disagree | Disagree | Neither Disagree nor Agree | Agree | Strongly Agree |
| --- | --- | --- | --- | --- |
| 0 | 1 | 2 | 3 | 4 |

1. I feel confident in my ability to suggest solutions to problems to my partner.

| Strongly disagree | Disagree | Neither Disagree nor Agree | Agree | Strongly Agree |
| --- | --- | --- | --- | --- |
| 0 | 1 | 2 | 3 | 4 |

1. I feel confident I could discuss problems with my partner without making him unhappy.

| Strongly disagree | Disagree | Neither Disagree nor Agree | Agree | Strongly Agree |
| --- | --- | --- | --- | --- |
| 0 | 1 | 2 | 3 | 4 |

1. I feel confident I could suggest a solution to a problem to my partner without making him

unhappy.

| Strongly disagree | Disagree | Neither Disagree nor Agree | Agree | Strongly Agree |
| --- | --- | --- | --- | --- |
| 0 | 1 | 2 | 3 | 4 |

1. I feel confident in my ability to persuade my partner to do something.

| Strongly disagree | Disagree | Neither Disagree nor Agree | Agree | Strongly Agree |
| --- | --- | --- | --- | --- |
| 0 | 1 | 2 | 3 | 4 |

## 1.9. Translated Multicultural Mastery Scale

Indicate your level of agreement along the scale below:

1. Working together with friends I can solve many of my problems.

|  |  |
| --- | --- |
|  |  |

Not at all Somewhat A lot

1. Working together with family I can solve many of my problems.

|  |  |
| --- | --- |
|  |  |

Not at all Somewhat A lot

1. I can change many of the important things in my life with the help of my friends.

|  |  |
| --- | --- |
|  |  |

Not at all Somewhat A lot

1. I can change many of the important things in my life with the help of my family.

|  |  |
| --- | --- |
|  |  |

Not at all Somewhat A lot

1. I can do what I set my mind to do because I have the support of my friends.

|  |  |
| --- | --- |
|  |  |

Not at all Somewhat A lot

1. I can do what I set my mind to do because I have the support of my family.

|  |  |
| --- | --- |
|  |  |

Not at all Somewhat A lot

1. What happens to me in the future mostly depends on my being supported by friends.

|  |  |
| --- | --- |
|  |  |

Not at all Somewhat A lot

1. What happens to me in the future mostly depends on my being supported by family.

|  |  |
| --- | --- |
|  |  |

Not at all Somewhat A lot

1. I can get what I want by helping my friends get what they want.

|  |  |
| --- | --- |
|  |  |

Not at all Somewhat A lot

1. I can get what I want by helping my family get what they want.

|  |  |
| --- | --- |
|  |  |

Not at all Somewhat A lot

1. I can solve many of the problems I have on my own.

|  |  |
| --- | --- |
|  |  |

Not at all Somewhat A lot

1. I can change many of the important things in my life.

|  |  |
| --- | --- |
|  |  |

Not at all Somewhat A lot

1. I do well even when things are tough.

|  |  |
| --- | --- |
|  |  |

Not at all Somewhat A lot

1. What happens to me mostly depends on me.

|  |  |
| --- | --- |
|  |  |

Not at all Somewhat A lot

1. I can do just about anything I really set my mind to do.

|  |  |
| --- | --- |
|  |  |

Not at all Somewhat A lot

## 1.10. Enhancing Assessment of Common Therapeutic factors (ENACT) rating scale

| **Date ___ ___ / ___ ___ / ___ ___ ___ ___**  Day Month Year |  |
| --- | --- |

ID number for person being observed: __________ Age: ______ Gender: M/F

Name of the Person completing the form:______________________ Job position: _________

Location of assessment__________________

| 1. Non-Verbal Communication & communication through active listening and with the use of appropriate body language __ Not applicable | | |
| --- | --- | --- |
| 1 | Need improvement | = does not make appropriate eye contact with the patient or stares; shows anger; laughs at/mocks patient; turned away from patient; repeatedly interrupts the patient conversation; ignores patient; answers mobile phone without permission |
| 2 | Done Partially | = does not consistently use body language to express interest: rarely makes eye contact, expresses only limited emotion, appears artificial; |
| 3 | Done Well | = makes appropriate eye contact throughout their conversation; smiles when appropriate; sits at appropriate angle from patient, leans in to the patient to show interest; use of ‘uh-huh’, ‘hmm’ and other keys to signal interest in their conversation |
| 2. Verbal Communication Skills: Open-ended questions, REPEATING THE MAIN TOPIC, clarifying statements__ Not applicable | | |
| 1 | Need Improvement | = Uses mostly ‘yes/no’ questions, e.g. “do you take your medication?” |
| 2 | Done Partially | = Uses open-ended questions, but does not explore topics further or does not repeats the main topics for patient to reflect upon |
| 3 | Done Well | = Uses Open-ended questions, repeats the main topic and clarifies statements, e.g.,asks questions like “What happened? Tell me more.” |
| 3. building trust __ Not applicable | | |
| 1 | Need improvement | = clinician does not attempt to make the patient feel comfortable by treating her with respect and dignity |
| 2 | Done Partially | = Clinician does not attempt to make the patient feel comfortable but treats her with respect and dignity. |
| 3 | Done Well | = Clinician attempts to make the patient feel comfortable by treating her with respect and dignity. |
| 4. further Exploration, interpretation and normalization of feelings __ Not applicable | | |
| 1 | Need improvement | = clinician does not ask about patient’s feelings OR clinician is judgmental/critical about patient’s emotions and feelings (e.g., “You shouldn’t feel that way” “You should stop thinking or feeling that.” |
| 2 | Done Partially | = clinician asks but does not normalize (does not explain that it is common)/validate OR does not explore feelings in detail with patient ( Uses questions which need a Yes/No reply) |
| 3 | Done Well | = clinician explains that the patient’s feelings are common and expected for a person in his/her situation |
| 5. Empathy, Warmth, & Genuineness __ Not applicable | | |
| 1 | Need improvement | = is critical, or hostile, of patient’s concerns or complaints |
| 2 | Done Partially | = clinician is not critical or hostile but does not demonstrate that he/she understands the experience of patient or does not consider him seriously. |
| 3 | Done Well | = clinician demonstrates that he/she understands the experience of patient in genuine, sincere manner |
| 6. Assessing Impact of psychosocial problems on Life, functioning and day to day activity __ Not applicable | | |
| 1 | Need improvement | = clinician does not ask patient about the impact of stress, worry, thoughts and psychosocial problems on functioning and daily life. |
| 2 | Done Partially | = clinician asks functioning and day to day activities, but does NOT relate it to psychosocial concerns |
| 3 | Done Well | = clinician explores the relationship between psychosocial problem and functioning |
| 7. Explores patient's and social support network's Explanation for the cause of Problem (Causal Model) __ Not applicable | | |
| 1 | Need improvement | = clinician does not ask patient about his/her own view of the cause of his problem OR is judgmental/critical about patient’s explanation (e.g. “Witchcraft doesn’t cause these problems, that is an ignorant/backwards idea |
| 2 | Done Partially | = clinician asks patient about his/her own view of cause of problems, but does not explore if his/her view is similar to his family or other important people in support network. |
| 3 | Done Well | = clinician asks patient about his own view of the cause of his problem and asks if family or significant other support network have same or different explanations |
| 8. Assessing coping mechanisms for problems and challenges and prior solutions __ Not applicable | | |
| 1 | Need improvement | = clinician does not ask patient about how patient has coped with the problem OR clinician is judgmental/critical about how patient has coped (e.g., “Why did you think that work?” or “That isn’t helpful.”) |
| 2 | Done Partially | = clinician asks about coping and prior solutions, but does not provide positive feedback |
| 3 | Done Well | = clinician asks patient about how he has coped with the problem and provides positive feedback |

| 9. Assessing impact of patient's recent stressful life events on psychosocial wellbeing__ Not applicable | | |
| --- | --- | --- |
| 1 | Need improvement | = clinician does not ask about current stressful situations/events |
| 2 | Done Partially | = clinician asks about current stressful situations but does not see its relation with current mental health issues |
| 3 | Done Well | = clinician asks about current stressful situations and discusses connection with current mental health |
|  | | |
| 10. assessing, Alcohol or Drugs USE (including misuse of prescription drugs) __ Not applicable | | |
| 1 | Need improvement | = clinician does not ask about drug and alcohol use (including misuse of prescription drugs) , OR asks about drug or alcohol use in an inappropriate or insensitive way for the patient’s age and sex |
| 2 | Done Partially | = clinician takes partial history but does not explore positive responses about alcohol or drug use (including misuse of prescription drugs) |
| 3 | Done Well | = clinician assesses issues with alcohol or drugs (including misuse of prescription drugs) and explains relationship to patient’s condition when appropriate OR clinician does not ask about drug and alcohol use but this is appropriate to age and gender of patient OR clinician asks about alcohol or drug use in close family members |
| 11. Assessing Appropriate involvement of family member, significant other and caregiver __ Not applicable | | |
| 1 | Need improvement | = clinician only talks with the patient’s family and does not give the appropriate respect for the feedback of the patient and ignores patient perspective, (e.g., “You should listen to your family more.)OR (if family not present) fails to ask the patient about the involvement of the family |
| 2 | Done Partially | = clinician ask about family involvement, but does not explore patient’s reasons for involvement or non-involvement |
| 3 | Done Well | = clinician makes a treatment plan that considers the patient’s perspective on how much they want family involvement (even if the family is not present) and encourages interaction between the two |
| 12. Assessing Collaborative goals setting and expectations of the patient for recovery__ Not applicable | | |
| 1 | Need improvement | = clinician does not ask patient about his/her goals for recovery OR clinician just tells patient what to do without asking his/her opinion |
| 2 | Done Partially | = clinician asks patient about goals for recovery but does not discuss if these are realistic or can be accomplished |
| 3 | Done Well | = clinician asks about goals regarding the treatment and discusses with patient what is and is not realistic and achievable through treatment; collaboratively clinician and patient establish treatment plan |

| 13. promotion of realistic hope for change__ Not applicable | | |
| --- | --- | --- |
| 1 | Need improvement | = clinician either gives no hope (e.g. you will never get better) or gives unrealistic expectations (e.g. you will be cured in a few weeks and never have problems again) for what to expect in treatment and recovery |
| 2 | Done Partially | = clinician vaguely tells patient what will happen during treatment |
| 3 | Done Well | = clinician helps patient feel positive about the future and creates realistic expectations about what can and cannot be achieved through treatment and explains treatment checking patient understanding |
| 14. Assessing the usE of Local (ethnopsychological) terms in conducting psychoeducation__ Not applicable | | |
| 1 | Need improvement | = clinician uses technical jargon to explain about mental illness OR uses stigmatizing terms OR does not explain how treatment works |
| 2 | Done Partially | = clinician uses a limited amount of technical jargon but No stigmatizing terms |
| 3 | Done Well | = clinician conducts psychoeducation using local terminology and phrases to explain mental health and treatment in non-stigmatizing language, in a local language where appropriate, and checks to see if patient understands |
| 15. ASSESSING Problem Solving SKILLs: Problem Formulation & Prioritization, Solution Generation, Action Planning __ Not applicable | | |
| 1 | Need improvement | =Clinician attempts problem solving steps #2-4 (see below) but only completes 1 or 2 steps satisfactorily |
| 2 | Done Partially | =Clinician attempts problem solving steps #2-4 (see below) but only completes 3 steps satisfactorily |
| 3 | Done Well | =clinician helps patient to do all of the following (1) formulate and prioritize primary problem, (2) brainstorm solutions, (3) explores advantages and disadvantages, and (4) formulate action plan |
| 16. asking for Feedback and Providing advice, suggestions and recommendations__ Not applicable | | |
| 1 | Need improvement | = clinician lectures patient what to do without asking if this is acceptable and comfortable to patient, |
| 2 | Done Partially | = clinician gives useful advice but does not ask for feedback from patient about the usefulness of the advice to the patient |
| 3 | Done Well | = clinician gives appropriate advice for the patient and explicitly asks for feedback about the usefulness of the advice |

| 17. Clinician explains confidentiality of their discussion__ Not applicable | | | | |  |
| --- | --- | --- | --- | --- | --- |
| 1 | | Need improvement | = clinician does not address confidentiality (by explaining confidentiality or ensuring privacy) OR does not adjust conversation to setting (e.g. if other family members are present, does not take care with topics discussed) | |  |
| 2 | | Done Partially | = clinician tells patient that everything is confidential with explaining harm to self or others | |  |
| 3 | | Done Well | = clinician explains that all clinician-patient discussions are confidential with the exception of harm to self and others OR ensures privacy OR adjusts conversation to setting | |  |
| 18. Harm to self, harm to others, and harm from others and collaborative response plan__ Not applicable | | | | | |
| 1 | | Need improvement | | = clinician does not ask about harm to self or others or does not pick up on key signals of self harm or suicide | |
| 2 | | Done Partially | | = clinician asks about harm to self or others, but does not help patient to develop a crisis plan | |
| 3 | | Done Well | | = clinician asks about harm to self or others and facilitates appropriate actions to assure safety | |
| 19 Assesses for potential role of community or social networks in finding solutions to problems __ Not applicable | | | | | |
| 1 | Need Improvement | | Does not assess the role of community or social networks as potential solutions to improve patient’s situation | | |
| 2 | Done Partially | | Makes suggestions about role of community or social networks as potential solutions to improve patient’s situation without asking for patient input | | |
| 3 | Done Well | | makes appropriate suggestions and asks for feedback, about the role of strengthening community engagement or social networks as a potential solution to improve patient’s situation | | |

## 1.11. MINI Suicidality Scale

## 1.12. List of Threatening Experiences questionnaire

| Have you experienced any of the following in the past 12 months? | | | |
| --- | --- | --- | --- |
| 1 | You yourself suffered a serious illness, injury, or an assault | Yes | 1 |
|  |  | No | 2 |
| 2 | A serious illness, injury, or assault happened to a close relative | Yes | 1 |
|  |  | No | 2 |
| 3 | Your parent, child, or spouse died | Yes | 1 |
|  |  | No | 2 |
| 4 | A close family friend or another relative (aunt, cousin, grandparent) died | Yes | 1 |
|  |  | No | 2 |
| 5 | You had a separation due to marital difficulties | Yes | 1 |
|  |  | No | 2 |
| 6 | You broke off a steady relationship | Yes | 1 |
|  |  | No | 2 |
| 7 | You had a serious problem with a close friend, neighbor, or relative | Yes | 1 |
|  |  | No | 2 |
| 8 | You became unemployed or you were seeking work unsuccessfully for more than one month | Yes | 1 |
|  |  | No | 2 |
| 9 | You were sacked from your job | Yes | 1 |
|  |  | No | 2 |
| 10 | You had a major financial crisis | Yes | 1 |
|  |  | No | 2 |
| 11 | You had problems with the police and a court appearance | Yes | 1 |
|  |  | No | 2 |
| 12 | Something you valued was lost or stolen | Yes | 1 |
|  |  | No | 2 |

## 1.13. Household Food Insecurity Access Scale

| In the past four weeks… | | | | | |
| --- | --- | --- | --- | --- | --- |
| 1 | Did you worry that your household would not have enough food? | Yes | [If yes] how often did this happen? | Rarely (once or twice in the past four weeks) | 1 |
|  |  | No (scores 0)  Skip to Q2 |  | Sometimes (three to ten times in the past four weeks) | 2 |
|  |  |  |  | Often (more than ten times in the past four weeks) | 3 |
| 2 | Were you or any household member not able to eat the kinds of  foods you preferred because of a lack of resources? | Yes | [If yes] how often did this happen? | Rarely (once or twice in the past four weeks) | 1 |
|  |  | No (scores 0)  Skip to Q3 |  | Sometimes (three to ten times in the past four weeks) | 2 |
|  |  |  |  | Often (more than ten times in the past four weeks) | 3 |
| 3 | Did you or any household member have to eat a limited variety of foods due to a lack of resources? | Yes | [If yes] how often did this happen? | Rarely (once or twice in the past four weeks) | 1 |
|  |  | No (scores 0)  Skip to Q4 |  | Sometimes (three to ten times in the past four weeks) | 2 |
|  |  |  |  | Often (more than ten times in the past four weeks) | 3 |
| 4 | Did you or any household member have to eat some foods that you really did not want to eat because of a lack of resources to obtain other types of food? | Yes | [If yes] how often did this happen? | Rarely (once or twice in the past four weeks) | 1 |
|  |  | No (scores 0)  Skip to Q5 |  | Sometimes (three to ten times in the past four weeks) | 2 |
|  |  |  |  | Often (more than ten times in the past four weeks) | 3 |
| 5 | Did you or any household member have to eat a smaller meal than you felt you needed because there was not enough food? | Yes | [If yes] how often did this happen? | Rarely (once or twice in the past four weeks) | 1 |
|  |  | No (scores 0)  Skip to Q6 |  | Sometimes (three to ten times in the past four weeks) | 2 |
|  |  |  |  | Often (more than ten times in the past four weeks) | 3 |
| 6 | Did you or any household member have to eat fewer meals in a day because there was not enough food? | Yes | [If yes] how often did this happen? | Rarely (once or twice in the past four weeks) | 1 |
|  |  | No (scores 0)  Skip to Q7 |  | Sometimes (three to ten times in the past four weeks) | 2 |
|  |  |  |  | Often (more than ten times in the past four weeks) | 3 |
| 7 | Was there ever no food to eat of any kind in your household because of lack of resources to get food? | Yes | [If yes] how often did this happen? | Rarely (once or twice in the past four weeks) | 1 |
|  |  | No (scores 0)  Skip to Q8 |  | Sometimes (three to ten times in the past four weeks) | 2 |
|  |  |  |  | Often (more than ten times in the past four weeks) | 3 |
| 8 | Did you or any household member go to sleep at night hungry because there was not enough food? | Yes | [If yes] how often did this happen? | Rarely (once or twice in the past four weeks) | 1 |
|  |  | No (scores 0)  Skip to Q9 |  | Sometimes (three to ten times in the past four weeks) | 2 |
|  |  |  |  | Often (more than ten times in the past four weeks) | 3 |
| 9 | Did you or any household member go a whole day and night without eating anything because there was not enough food? | Yes | [If yes] how often did this happen? | Rarely (once or twice in the past four weeks) | 1 |
|  |  | No (scores 0) |  | Sometimes (three to ten times in the past four weeks) | 2 |
|  |  |  |  | Often (more than ten times in the past four weeks) | 3 |

## 1.14. Oslo Social Support Scale (OSSS-3)

| 1 | How many people are so close to you that you can count on them if you have great personal problems? | None | 1 |
| --- | --- | --- | --- |
|  |  | 1-2 | 2 |
|  |  | 3-5 | 3 |
|  |  | More than 5 | 4 |
| 2 | How much interest and concern do people show in what you do? | None | 1 |
|  |  | Little | 2 |
|  |  | Uncertain | 3 |
|  |  | Some | 4 |
|  |  | A Lot | 5 |
| 3 | How easy is it to get practical help from neighbours if you should need it? | Very difficult | 1 |
|  |  | Difficult | 2 |
|  |  | Possible | 3 |
|  |  | Easy | 4 |
|  |  | Very easy | 5 |

## 1.15. Helping Alliance Questionnaire


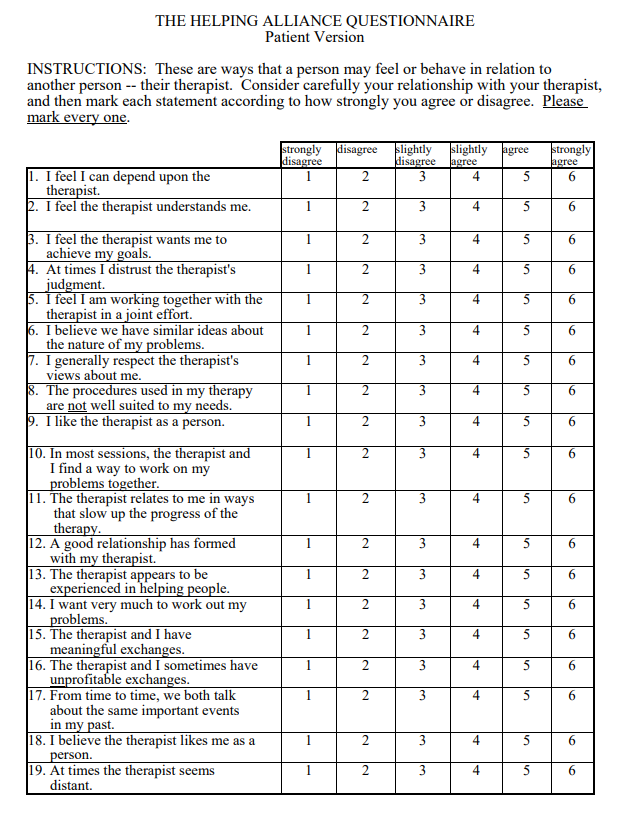


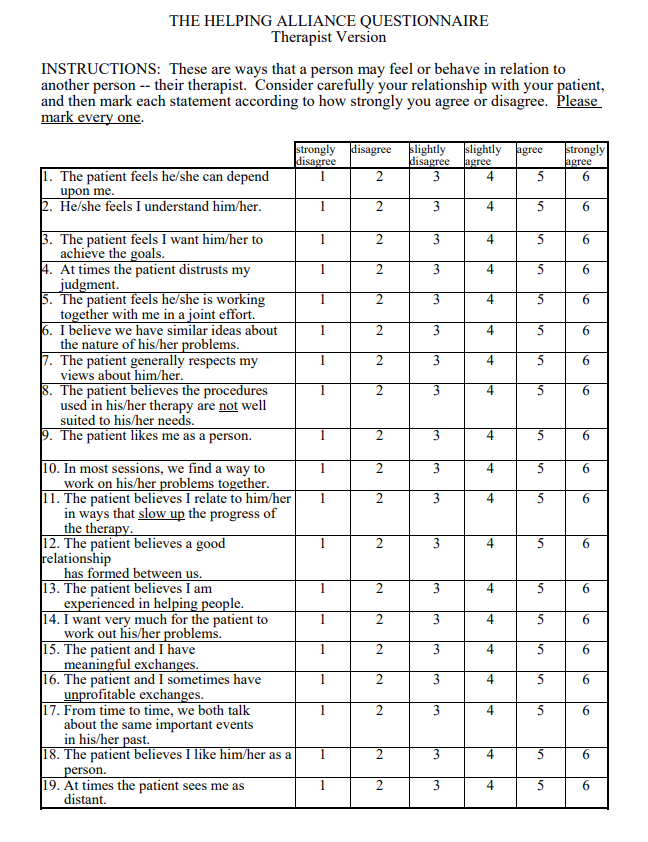


## 1.6. Modified Client service receipt inventory (CSRI)

| 101 | **In the past 3 months when you have had a health problem, what face-to-face contacts have you had with these professionals?**  **(excluding in-patient care)** | | | | | | | | | | | | | | | | |  |  |
| --- | --- | --- | --- | --- | --- | --- | --- | --- | --- | --- | --- | --- | --- | --- | --- | --- | --- | --- | --- |
| 101A | Health extension worker | | | Yes | 1 | | 101B | | | | | | | | | CHEW | | |  |
|  |  |  |  | No | 0 | |  |  |  |  |  |  |  |  |  |  |  |  |  |
|  |  | A | Where were you seen? | | | | | | Health post | | | | | | 1 | CHEWLO | | |  |
|  |  |  |  |  |  |  |  |  | Home | | | | | | 0 |  |  |  |  |
|  |  | B | How many times?  (in last 3 months) | | | | | | [ ] [ ]? | | | | | | | CHEWFR | | |  |
|  |  | C | Time taken for travel (both ways) | | | | | | [ ] [ ] hours | | | | | | | CHEWTR | | |  |
|  |  | D | Travel Cost | | | | | | [ ] [ ] [ ] [ ] Birr | | | | | | | CHEWEX | | |  |
|  |  | E | Accommodation cost | | | | | | [ ] [ ] [ ] [ ] Birr | | | | | | | CHEWAC | | |  |
|  |  | F | How many adults accompanied you? | | | | | | [ ] [ ] adults | | | | | | | CHEWNU | | |  |
|  |  | G | Total time taken with HEW / at health post (includes waiting, consultation and investigations) | | | | | | [ ] [ ] hours | | | | | | | CHEWDU | | |  |
|  |  | H | Total cost of medication and investigations | | | | | | [ ] [ ] [ ] [ ] Birr | | | | | | | CHEWME | | |  |
| 101B | Health centre | | | Yes | 1 | | 101C | | | | | | | | | CHC | | |  |
|  |  |  |  | No | 0 | |  |  |  |  |  |  |  |  |  |  |  |  |  |
|  |  | A | How many times?  (in last 3 months) | | | | | | [ ] [ ]? | | | | | | | CHCFR | | |  |
|  |  | B | Time taken for travel (both ways) | | | | | | [ ] [ ] hours | | | | | | | CHCTR | | |  |
|  |  | C | Travel cost | | | | | | [ ] [ ] [ ] [ ] Birr | | | | | | | CHCEX | | |  |
|  |  | D | Accommodation cost | | | | | | [ ] [ ] [ ] [ ] Birr | | | | | | | CHCAC | | |  |
|  |  | E | How many adult accompanied you? | | | | | | [ ] [ ] adults | | | | | | | CHCNU | | |  |
|  |  | F | Total time taken at health centre (includes waiting, consultation and investigations) | | | | | | [ ] [ ] hours | | | | | | | CHCDU | | |  |
|  |  | G | Total cost of medication and investigations | | | | | | [ ] [ ] [ ] [ ] Birr | | | | | | | CHCME | | |  |
| 101C | Public hospital (for out-patient care) | | | Yes | 1 | | 101D | | | | | | | | | CHOS | | |  |
|  |  |  |  | No | 0 | |  |  |  |  |  |  |  |  |  |  |  |  |  |
|  |  | A | How many times?  (in last 3 months) | | | | | | [ ] [ ]? | | | | | | | CHOSFR | | |  |
|  |  | B | Time taken for travel (both ways) | | | | | | [ ] [ ] hours | | | | | | | CHOSTR | | |  |
|  |  | C | Travel cost | | | | | | [ ] [ ] [ ] [ ] Birr | | | | | | | CHOSEX | | |  |
|  |  | D | Accommodation cost | | | | | | [ ] [ ] [ ] [ ] Birr | | | | | | | CHOSAC | | |  |
|  |  | E | How many adults accompanied you? | | | | | | [ ] [ ] adults | | | | | | | CHOSNU | | |  |
|  |  | F | Total time taken at hospital (includes waiting, consultation and investigations) | | | | | | [ ] [ ] hours | | | | | | | CHOSDU | | |  |
|  |  | G | Total cost of medication and investigations | | | | | | [ ] [ ] [ ] [ ] Birr | | | | | | | CHOSME | | |  |
| 101D | Private / NGO Hospital (for out-patient care | | | Yes | 1 | | 101E | | | | | | | | | CPHOS | | |  |
|  |  |  |  | No | 0 | |  |  |  |  |  |  |  |  |  |  |  |  |  |
|  |  | A | How many times?  (in last 3 months) | | | | | | [ ] [ ]? | | | | | | | CPHOSFR | | |  |
|  |  | B | Time taken for travel (both ways) | | | | | | [ ] [ ] hours | | | | | | | CPHOSTR | | |  |
|  |  | C | Travel cost | | | | | | [ ] [ ] [ ] [ ] Birr | | | | | | | CPHOSEX | | |  |
|  |  | D | Accommodation cost | | | | | | [ ] [ ] [ ] [ ] Birr | | | | | | | CPHOSAC | | |  |
|  |  | E | How many adults accompanied you? | | | | | | [ ] [ ] adults | | | | | | | CPHOSNU | | |  |
|  |  | F | Total time taken at hospital (includes waiting, consultation and investigations) | | | | | | [ ] [ ] hours | | | | | | | CPHOSDU | | |  |
|  |  | G | Total cost of medication and investigations | | | | | | [ ] [ ] [ ] [ ] Birr | | | | | | | CPHOSME | | |  |
| 101E | Private clinic  (out-patient service) | | | Yes | 1 | | 101F | | | | | | | | | CPC | | |  |
|  |  |  |  | No | 0 | |  |  |  |  |  |  |  |  |  |  |  |  |  |
|  |  | A | How many times?  (in last 3 months) | | | | | | [ ] [ ]? | | | | | | | CPCFR | | |  |
|  |  | B | Time taken for travel (both ways) | | | | | | [ ] [ ] hours | | | | | | | CPCTR | | |  |
|  |  | C | Travel cost | | | | | | [ ] [ ] [ ] [ ] Birr | | | | | | | CPCEX | | |  |
|  |  | D | Accommodation cost | | | | | | [ ] [ ] [ ] [ ] Birr | | | | | | | CPCAC | | |  |
|  |  | E | How many adults accompanied you? | | | | | | [ ] [ ] adults | | | | | | | CPCNU | | |  |
|  |  | F | Total time taken at private clinic (includes waiting, consultation and investigations) | | | | | | [ ] [ ] hours | | | | | | | CPCDU | | |  |
|  |  | G | Total cost of medication and investigations | | | | | | [ ] [ ] [ ] [ ] Birr | | | | | | | CPCME | | |  |
| 101F | Pharmacy / drug vender | | | Yes | 1 | | 101G | | | | | | | | | CPHA | | |  |
|  |  |  |  | No | 0 | |  |  |  |  |  |  |  |  |  |  |  |  |  |
|  |  | A | How many times?  (in last 3 months) | | | | | | [ ] [ ]? | | | | | | | CPHAFR | | |  |
|  |  | B | Time taken for travel (both ways) | | | | | | [ ] [ ] hours | | | | | | | CPHATR | | |  |
|  |  | C | Travel cost | | | | | | [ ] [ ] [ ] [ ] Birr | | | | | | | CPHAEX | | |  |
|  |  | D | Accommodation cost | | | | | | [ ] [ ] [ ] [ ] Birr | | | | | | | CPHAAC | | |  |
|  |  | E | How many adults accompanied you? | | | | | | [ ] [ ] adults | | | | | | | CHPHANU | | |  |
|  |  | F | Total time taken at pharmacy | | | | | | [ ] [ ] hours | | | | | | | CPHADU | | |  |
|  |  | G | Total cost of medication and investigations | | | | | | [ ] [ ] [ ] [ ] Birr | | | | | | | CPHAME | | |  |
| 101G | Holy water | | | Yes | 1 | | 101H | | | | | | | | | CHOL | | |  |
|  |  |  |  | No | 0 | |  |  |  |  |  |  |  |  |  |  |  |  |  |
|  |  | A | How many times?  (in last 3 months) | | | | | | [ ] [ ]? | | | | | | | CHOLFR | | |  |
|  |  | B | Time taken for travel (both ways) | | | | | | [ ] [ ] hours | | | | | | | CHOLTR | | |  |
|  |  | C | Travel cost | | | | | | [ ] [ ] [ ] [ ] Birr | | | | | | | CHOLEX | | |  |
|  |  | D | Accommodation cost | | | | | | [ ] [ ] [ ] [ ] Birr | | | | | | | CHOLAC | | |  |
|  |  | E | How many days did any adult with you miss from work? | | | | | | [ ] [ ] adults | | | | | | | CHOLNU | | |  |
|  |  |  |  |  |  |  |  |  | Adult 1 | | | [ ] [ ] days | | | | CHOLA1  CHOLA2  CHOLA3 | | |  |
|  |  |  |  |  |  |  |  |  | Adult 2 | | | [ ] [ ] days | | | |  |  |  |  |
|  |  |  |  |  |  |  |  |  | Adult 3 | | | [ ] [ ] days | | | |  |  |  |  |
|  |  | F | Total time spent at holy water (for all visits in the last 3 months) | | | | | | [ ] [ ] days | | | | | | | CHOLDU | | |  |
|  |  | G | Total cost of consultation and intervention | | | | | | [ ] [ ] [ ] [ ] Birr | | | | | | | CHOLME | | |  |
| 101H | Religious leader or priest | | | Yes | 1 | | 101J | | | | | | | | | CREL | | |  |
|  |  |  |  | No | 0 | |  |  |  |  |  |  |  |  |  |  |  |  |  |
|  |  | A | How many times?  (in last 3 months) | | | | | | [ ] [ ]? | | | | | | | CRELFR | | |  |
|  |  | B | Time taken for travel (both ways) | | | | | | [ ] [ ] hours | | | | | | | CRELTR | | |  |
|  |  | C | Travel cost | | | | | | [ ] [ ] [ ] [ ] Birr | | | | | | | CRELEX | | |  |
|  |  | D | Accommodation cost | | | | | | [ ] [ ] [ ] [ ] Birr | | | | | | | CRELAC | | |  |
|  |  | E | How many adults accompanied you? | | | | | | [ ] [ ] adults | | | | | | | CRELNU | | |  |
|  |  | F | Total time taken with priest / religious leader / healer (includes waiting and consultation) | | | | | | [ ] [ ] hours | | | | | | | CRELDU | | |  |
|  |  | G | Total cost of consultation and intervention | | | | | | [ ] [ ] [ ] [ ] Birr | | | | | | | CRELME | | |  |
| 101J | Muslim healer / Kalicha | | | Yes | 1 | | 101K | | | | | | | | | CKAL | | |  |
|  |  |  |  | No | 0 | |  |  |  |  |  |  |  |  |  |  |  |  |  |
|  |  | A | How many times?  (in last 3 months) | | | | | | [ ] [ ]? | | | | | | | CKALFR | | |  |
|  |  | B | Time taken for travel (both ways) | | | | | | [ ] [ ] hours | | | | | | | CKALTR | | |  |
|  |  | C | Travel cost | | | | | | [ ] [ ] [ ] [ ] Birr | | | | | | | CKALEX | | |  |
|  |  | D | Accommodation cost | | | | | | [ ] [ ] [ ] [ ] Birr | | | | | | | CKALAC | | |  |
|  |  | E | How many adults accompanied you? | | | | | | [ ] [ ] adults | | | | | | | CKALNU | | |  |
|  |  | F | Total time taken with kalicha (includes waiting and consultation) | | | | | | [ ] [ ] hours | | | | | | | CKALDU | | |  |
|  |  | G | Total cost of consultation and intervention | | | | | | [ ] [ ] [ ] [ ] Birr | | | | | | | CKALME | | |  |
| 101K | Herbalist | | | Yes | 1 | | 101L | | | | | | | | | CHER | | |  |
|  |  |  |  | No | 0 | |  |  |  |  |  |  |  |  |  |  |  |  |  |
|  |  | A | How many times?  (in last 3 months) | | | | | | [ ] [ ]? | | | | | | | CHERFR | | |  |
|  |  | B | Time taken for travel (both ways) | | | | | | [ ] [ ] hours | | | | | | | CHERTR | | |  |
|  |  | C | Travel cost | | | | | | [ ] [ ] [ ] [ ] Birr | | | | | | | CHEREX | | |  |
|  |  | D | Accommodation cost | | | | | | [ ] [ ] [ ] [ ] Birr | | | | | | | CHERAC | | |  |
|  |  | E | How many adults accompanied you? | | | | | | [ ] [ ] adults | | | | | | | CHERNU | | |  |
|  |  | F | Total time taken with herbalist (includes waiting and consultation) | | | | | | [ ] [ ] hours | | | | | | | CHERDU | | |  |
|  |  | G | Total cost of consultation and intervention (e.g. herbal remedy) | | | | | | [ ] [ ] [ ] [ ] Birr | | | | | | | CHERME | | |  |
| 101L | Wogesha | | | Yes | 1 | | 101M | | | | | | | | | CWO | | |  |
|  |  |  |  | No | 0 | |  |  |  |  |  |  |  |  |  |  |  |  |  |
|  |  | A | How many times?  (in last 3 months) | | | | | | [ ] [ ]? | | | | | | | CWOFR | | |  |
|  |  | B | Time taken for travel (both ways) | | | | | | [ ] [ ] hours | | | | | | | CWOTR | | |  |
|  |  | C | Travel cost | | | | | | [ ] [ ] [ ] [ ] Birr | | | | | | | CWOEX | | |  |
|  |  | D | Accommodation cost | | | | | | [ ] [ ] [ ] [ ] Birr | | | | | | | CWOAC | | |  |
|  |  | E | How many adults accompanied you? | | | | | | [ ] [ ] adults | | | | | | | CWONU | | |  |
|  |  | F | Total time taken with wogesha (includes waiting, consultation) | | | | | | [ ] [ ] hours | | | | | | | CWODU | | |  |
|  |  | G | Total cost of consultation and intervention | | | | | | [ ] [ ] [ ] [ ] Birr | | | | | | | CWOME | | |  |
| 101M | Sorcerer (Tenquaye) | | | Yes | 1 | | 101N | | | | | | | | | CTNQ | | |  |
|  |  |  |  | No | 0 | |  |  |  |  |  |  |  |  |  |  |  |  |  |
|  |  | A | How many times?  (in last 3 months) | | | | | | [ ] [ ]? | | | | | | | CTNQFR | | |  |
|  |  | B | Time taken for travel (both ways) | | | | | | [ ] [ ] hours | | | | | | | CTNQTR | | |  |
|  |  | C | Travel cost | | | | | | [ ] [ ] [ ] [ ] Birr | | | | | | | CTNQEX | | |  |
|  |  | D | Accommodation cost | | | | | | [ ] [ ] [ ] [ ] Birr | | | | | | | CTNQAC | | |  |
|  |  | E | How many adults accompanied you? | | | | | | [ ] [ ] adults | | | | | | | CTNQNU | | |  |
|  |  | F | Total time taken with Tanquaye (includes waiting, consultation) | | | | | | [ ] [ ] hours | | | | | | | CTNQDU | | |  |
|  |  | KG | Total cost of consultation and intervention | | | | | | [ ] [ ] [ ] [ ] Birr | | | | | | | CTNQME | | |  |
| 101N | Other traditional healer | | | Yes | 1 | | 101P | | | | | | | | | COTR | | |  |
|  |  |  |  | No | 0 | |  |  |  |  |  |  |  |  |  |  |  |  |  |
|  |  | A | How many times?  (in last 3 months) | | | | | | [ ] [ ]? | | | | | | | COTRFR | | |  |
|  |  | B | Time taken for travel (both ways) | | | | | | [ ] [ ] hours | | | | | | | COTRTR | | |  |
|  |  | C | Travel cost | | | | | | [ ] [ ] [ ] [ ] Birr | | | | | | | COTREX | | |  |
|  |  | D | Accommodation cost | | | | | | [ ] [ ] [ ] [ ] Birr | | | | | | | COTRAC | | |  |
|  |  | E | How many adults accompanied you? | | | | | | [ ] [ ] adults | | | | | | | COTRNU | | |  |
|  |  | F | Total time taken with traditional healer (includes waiting, consultation) | | | | | | [ ] [ ] hours | | | | | | | COTRDU | | |  |
|  |  | K | Total cost of consultation and any intervention | | | | | | [ ] [ ] [ ] [ ] Birr | | | | | | | COTRME | | |  |
| 102 | **In the past 12 months have you needed any in-patient care for a health problem?** | | | | | | | Yes | | | 1 | 🡪 103 | | | | CIP | | |  |
|  |  |  |  |  |  |  |  | No | | | 0 |  |  |  |  |  |  |  |  |
| 102A | Public / government hospital? | | | Yes | 1 | | 101B | | | | | | | | | CGIP | | |  |
|  |  |  |  | No | 0 | |  |  |  |  |  |  |  |  |  |  |  |  |  |
|  |  | A | In the last 12 months, how many times were you in hospital? (as an in-patient) | | | | | | [ ] [ ] times | | | | | | | CGIPFR | | |  |
|  |  | B | In the last 12 months, how many days (IN TOTAL) were you in hospital (as an in-patient)? | | | | | | [ ] [ ] [ ]  total number of days | | | | | | | CGIPDA | | |  |
|  |  | C | Time taken for travel (both ways) | | | | | | [ ] [ ] hours | | | | | | | CGIPTR | | |  |
|  |  | D | Travel Cost | | | | | | [ ] [ ] [ ] [ ] Birr | | | | | | | CGIPEX | | |  |
|  |  | E | Accommodation cost | | | | | | [ ] [ ] [ ] [ ] Birr | | | | | | | CGIPAC | | |  |
|  |  | F | How many days did any adult stay with you? | | | | | | [ ] [ ] adults | | | | | | | CGIPNU | | |  |
|  |  |  |  |  |  |  |  |  | Adult 1 | | | [ ] [ ] days | | | | CGIPA1  CGIPA2  CGIPA3 | | |  |
|  |  |  |  |  |  |  |  |  | Adult 2 | | | [ ] [ ] days | | | |  |  |  |  |
|  |  |  |  |  |  |  |  |  | Adult 3 | | | [ ] [ ] days | | | |  |  |  |  |
|  |  | G | Total cost of medication and investigations and procedures | | | | | | [ ] [ ] [ ] [ ] Birr | | | | | | | CGIPME | | |  |
| 102B | Private / NGO hospital? | | | Yes | 1 | | 103 | | | | | | | | | CMPIP | | |  |
|  |  |  |  | No | 0 | |  |  |  |  |  |  |  |  |  |  |  |  |  |
|  |  | A | In the last 12 months, how many times were you in hospital? (as an in-patient) | | | | | | [ ] [ ] times | | | | | | | CMGIPFR | | |  |
|  |  | B | In the last 12 months, how many days (IN TOTAL) were you in hospital (as an in-patient)? | | | | | | [ ] [ ] [ ]  total number of days | | | | | | | CMGIPDA | | |  |
|  |  | C | Time taken for travel (both ways) | | | | | | [ ] [ ] hours | | | | | | | CMGIPTR | | |  |
|  |  | D | Travel Cost | | | | | | [ ] [ ] [ ] [ ] Birr | | | | | | | CMGIPEX | | |  |
|  |  | E | Accommodation cost | | | | | | [ ] [ ] [ ] [ ] Birr | | | | | | | CMGIPAC | | |  |
|  |  | F | How many days did any adult stay with you? | | | | | | [ ] [ ] adults | | | | | | | CMGIPNU | | |  |
|  |  |  |  |  |  |  |  |  | Adult 1 | | | [ ] [ ] days | | | | CMGIPA1  CMGIPA2  CMGIPA3 | | |  |
|  |  |  |  |  |  |  |  |  | Adult 2 | | | [ ] [ ] days | | | |  |  |  |  |
|  |  |  |  |  |  |  |  |  | Adult 3 | | | [ ] [ ] days | | | |  |  |  |  |
|  |  | G | Total cost of medication and investigations and procedures | | | | | | [ ] [ ] [ ] [ ] Birr | | | | | | | CMGIPME | | |  |
| 104 | During the past 3 months have you had help from family and friends in the following areas as a result of your health problems? | | | | | | | | | | | | |  | | |  | | |
|  |  | | | | | Personal care | | | | Yes | | | 1 | [ ] [ ] hours / week | | | CPERS | | |
|  |  |  |  |  |  |  |  |  |  | No | | | 2 |  |  |  | PERSHR | | |
|  |  |  |  |  |  | Providing transport / accompanying to clinic | | | | Yes | | | 1 | [ ] [ ] hours / week | | | CTRANS | | |
|  |  |  |  |  |  |  |  |  |  | No | | | 2 |  |  |  | CTRANSHR | | |
|  |  |  |  |  |  | Preparing meals | | | | Yes | | | 1 | [ ] [ ] hours / week | | | CMEAL | | |
|  |  |  |  |  |  |  |  |  |  | No | | | 2 |  |  |  | CMEALHR | | |
|  |  |  |  |  |  | Housework | | | | Yes | | | 1 | [ ] [ ] hours / week | | | CHOUS | | |
|  |  |  |  |  |  |  |  |  |  | No | | | 2 |  |  |  | CHOUSHR | | |
|  |  |  |  |  |  | Shopping | | | | Yes | | | 1 | [ ] [ ] hours / week | | | CSHOP | | |
|  |  |  |  |  |  |  |  |  |  | No | | | 2 |  |  |  | CSHOPHR | | |
|  |  |  |  |  |  | Socialising / attending social | | | | Yes | | | 1 | [ ] [ ] hours / week | | | CSOC | | |
|  |  |  |  |  |  |  |  |  |  | No | | | 2 |  |  |  | CSOCHR | | |

##

#
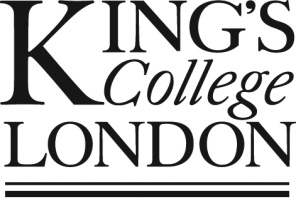
 Suplementary Files 4: Information Sheet (Amh Version)

APPENDIX 5: Information Sheet (Amharic Version)
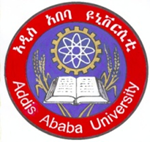


**ለተሳታፊዎች የተዘጋጀ ቅጽ**

IRB Reference Number፡

**የዚህ የመረጃ ወረቀት ግልባጭ በእጅዎ ይደርሳል**

በዚህ የጥናት ፕሮጀክት እንዲሳተፉ ተጋብዘዋል፡፡ ተሳትፎዎት በፍላጎት ላይ ብቻ የተመሰረተ መሆን አለበት፣ በጥናቱ ላለመሳተፍ ከመረጡ የሚያስከትለው ችግር የለም፡፡ በጥናቱ ለመሳተፍ ከመወሰንዎ በፊት ጥናቱ ለምን እንደሚሰራና የእርስዎ ተሳትፎ ምን እንደሚያካትት መረዳቱ አስፈላጊ ነው፡፡ እባክዎ ይህን ወረቀት ይውሰዱና ከዚህ በታች የተሰጡ መረጃዎችን በጥንቃቄ ለማስነበብ ጊዜ ይውሰዱ ከፈለጉም ከሌሎች ጋራ ይወያዩበት፡፡ ግልጽ ያልሆነ ነገር ካለና የበለጠ መረጃ ከፈለጉ ሊጠይቁን ይችላሉ፡፡

- **የምርምሩ አላማ**

ድባቴና ፆታዊ ትንኮሳ በሀገራችን በጣም የተለመዱ ችግሮች ናቸው፡፡ የእናቶችን ጤና፣ የመስራት አቅም፣ ልጅ የመንከባከብ አቅም ያዳክማሉ፡፡ ስለሆነም ከእንግሊዝ ዩኒቨርስቲና ከአዲስ አበባ የኒቨርስቲ ጋር በመተባበር ችግሮችን ለመቀነስ የሚያስችል ፕሮግራም አዘጋጅተናል፡፡

**በጥናቱ እንዲሳተፉ የሚመረጡት እነ ማን ናቸው**

ከላይ በተጠቀሰው ርእስ ላይ በዋነኝነት መረጃ ሊሰጡን የሚችሉት ነፍሰ-ጡር እና የወለዱ እናቶች ቢሆኑም ሌሎች መረጃው ይኖራቸዋል ብለን የምናስባቸው ሰዎችም ይካተታሉ፡፡ የጤና ባለሙያዎችን፤ የማህበረሰብ መሪዎችን፤ የሃይማኖትና የባህል ሃኪሞችን እና በአእምሮ ህመም የተነሳ የሚሰቃዩ እናቶችንና ቤተሰቦቻቸውን እናነጋግራለን፡፡

**በጥናቱ ለመሳተፍ ቢስማሙ ምን ይደረጋል፡**፡

በጥናቱ ላይ የሚሳተፉት በፍቃደኝነትዎ ላይ ተመስርቶ ነው፡፡ ያለመሳተፍ ይችላሉ፤ ምንም አይነት ጥያቄ ያለመመለስ መብት አሎት፡፡ መካከልም ጥያቄውን መመለስ ማቆም ይችላሉ፡፡ ጥያቄውን ያለመመለስ ወይም ሙሉ በሙሉ ማቋረጥ ለእርሶም ለቤተሰብዎም ለማህበረሰብዎም ልታገኙት የምትችሉትን ጥቅም አያሳጣም፡፡ መጠይቁም ከአንድ በላይ ንኡስ ክፍሎች አሉት፡፡ በመጀመሪያው ክፍል ስለ ትምህርትና ስለአጠቃላይ መረጃ በሁለተኛው በእርግዝና ጊዜ ስለሚከሰት ድባቴ ይመለከታሉ፡፡

በጥናቱ ለመሳተፍ ከተስማሙ ድባቴንና ከቤተሰብ ጋር ሊፈጠር የሚችለውን አለመስማማት የሚቀንስ ትምህርትና የምክክር አገልግሎት ይሰጣል፡፡ ከመረጃ ሰብሳቢዎቻችን አንዱ በስራ ቦታዎ ወይም ቤትዎ ድረስ በመምጣት አንዳንድ ጥያቄዎች ይቀርብሎታል፡፡ መጠይቆቹ በአገልግሎቱ ምክንያት የተገኘውን ለውጥ ከመገምገም የታቀዱ ናቸው፡፡ ቃለ መጠይቁ ወደ ግማሽ ሰአት ገደማ ይወስዳል፡፡ እርስዎ የሚፈቅዱ ከሆነ ቃለ ምልልሱን በመቅረጸ ድምጽ እንቀዳዋለን፡፡

- **በጥናቱ መሳተፍ ምን ጉዳት ይኖረዋል**?

በቃለመጠይቁ መሳተፍ የሚያስከትለው ችግር የለም፡፡ ቢሆንም አንዳንድ ሰዎች በሚነሱ ጥያቄዎች ላይመቻቸው ይችሉ ይሆናል፡፡ እርስዎ በጥያቄዎቹ ደስተኛ ካልሆኑ መልስ ይሰጡ ዘንድ አይገደዱም፡፡ ቃለ መጠይቁም ዕዚሁ ላይ መቆም ይችላል፡፡፣

የሚገኘው መረጃ በኢትዮጵያም ሆነ በሌሎች ሀገሮች ያለውን የአእምሮ ጤና አገልግሎት እንደሚያሻሽለው ተስፋ እናደረጋለን፡፡

ጥናቱ ሲጠናቀቅ ግኝታችንን ለማወቅ ይችሉ ዘንድ እርስዎን እንጋብዞታለን፡፡ አጭር የመረጃ ጽሁፍ እንሰጥዎታለን ወይም በወረዳው ውስጥ ለህዝብ እንዲገለጽ እናደርጋለን፡፡

**አማራጭ**

እርስዎ በጥናቱ ላይ ያለመሳተፍ መብት አለዎት፡፡ መሳተፍ ካልፈለጉ መሳተፍ የለብዎትም፡፡ በጥናቱ መሳተፍ የእርሶ ምርጫ ነው፤

ማንም ሊበሳጭብዎ/ሊያዝንብዎ አይችልም፤ መጀመሪያ ተስማመተው በመሃል ማቋረጥ ቢፈልጉም ጭምር፡፡

- **በሰጡን ቅድመ መረጃ ምን እናደርግበታለን?**

ጥያቄዎቹ የእርስዎን ስም አያካትቱም፡፡ ስለዚህ ከፕሮጀክቱ አስተባባሪዎች **ዶ/ር ተሠራ ቢተውና እና ዶ/ር ሮክሳን ኬኔጃድ** እና የፕሮጀክቱ የመረጃ ሰራተኞች ውጪ ማንም ሌላ ሰው መረጃው የእርስዎ ስለመሆኑ የሚያውቀው አይኖርም ፡፡

የመረጃ ሰነዶቹን በሚቆለፉ መሳቢያ / መደርደርያ / እናስቀምጣለን፡፡

ለድምጸ መቅረጹ ፍቃደኛ ከሆኑ ስምዎትንና እርስዎነቶን የሚያሳውቅ መረጃዎች አይቀረጹም፡፡

መቅረጸ ድምጹም በሚቆለፍ መደርደረያ ውስጥ ይቆለፍበታል፡፡

በመቅረፀ ድምጽ የተያዘው ቅድመ መረጃ ወደ ጽሑፍ ከተቀየረ በኋላ እና ከተተነተነ በኋላ በድምጽ የተቀረጸው ይሰረዛል፡፡

ከጥናቱ ማለቅ በኋላ የሰጡን መረጃ ሌሎች ተመራማሪዎች ይጠቀሙበት ይሆናል፡፡ ግን በማንኛውም መንገድ መረጃ የሰጠውን ሰው መለየት እናዳይችሉ ይደረጋል፡፡

በቃለ መጠይቁ ጊዜ የሚሰጡት መረጃ በሚስጥር የተጠበቀ መሆኑን አሳውቆታለሁ፡፡

ለፕሮጀክቱ የሚሰጡትን መረጃ ለማንም ሰው፤ (ለቤተሰብ ወይም ለባለቤትዎ) አይነገርም፡፡

ጥናቱ ሲመዘገብ በስምዎ ምትክ የሚስጥር ቁጥሮችን እንጠቀማለን፣ የሚስጥር ቁጥሮንም ማንም በማያውቅበት መልኩ ይሰጣል፡፡

መዝገቡም የአዲስ አበባ ዩኒቨርስቲ የአእምሮ ጤና ት/ክፍል ደህንነቱ ተጠብቆ እና ተቆልፎ ይቀመጣል፡፡

የምንነጋገርበት ጉዳይ ሁሉ በሚስጥር የተጠበቀ እና ለማንም የማይነገር ይሆናል፡፡

ይህ ጥናት በ2012 ዓ.ም ይጠናቀቃል፡፡ ኮምፒዩተር ላይ የሚቀመጠው መረጃ በኮድ የተቆለፈ ይሆናል፡፡

**ዋና አጥኚዎች**

የጥናቱን አስተባባሪ ደ/ር ተሠራ ቢተው ሲሆን ሊያገኙ ከፈለጉ ሞባይል ቁጥር 0911 17 36 56 በመጠቀም በስራ ሰአት በማንኛውም ቀን ሊደውሉልን ይችላሉ፡፡ በጥናቱ መሳተፍ የእርስዎ ውሳኔ ጉዳይ ይሆናል፡፡ በጥናቱ ለመሳተፍ ከወሰኑ በማንኛውም ሰአት ምክንያት መስጠት ሳይጠበቅብዎት በነጻነት ተሳትፎውን ማቋረጥ ይችላሉ፡፡

ይህ ጥናት በማንኛውም መንገድ ጉዳት ካደረሰብዎት የአ.አ ዩኒቨርሲቲ የህክምና ፋኩልቲ የስነምግባር (ኢቲክስ) ተቋማዊ የክለሳ ቦርድን በስልክ ቁጥር 0115-553 87 34 ማነጋገር ይችላሉ፡፡

| **ማስታወሻ፡** . ወደ መጨረሻ ሪፖርትነት እስኪቀየር ድረስ በፈለጉት ሰአት መረጃዎን ከፕሮጀክቱ ሊያወጡ ይችላሉ፡፡  . በጥናቱ ለመሳተፍ ከወሰኑ ይህን የመረጃ ቅጽ ይሰጥዎትና ስምምነት ግን በፊርማ እንዲያረጋግጡ ይጠየቃሉ፡፡ |
| --- |

#
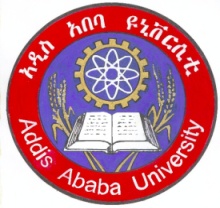
APPENDIX 5: Consent Form (Amharic Version)

**CONSENT FORM**

Please complete this form after you have read the Information Sheet and/or listened to an explanation about the research.

**በጥናቱ ላይ ስለመሣተፍ የስምምነት መግለጫ ቅፅ**

**የጥናቱ ርዕስ፡- የቅድመ-ወሊድ ድባቴና በጥንዶች መካከል ስለሚኖረው አለመስማማት**

የአ.አ.ዩ የምርምር ስነ-ምግባር ኮሚቴ መለያ ቁጥር **024/14/psy**

በዚህ ምርምር ለመሳተፍ ስላሰቡ እናመሰግናለን፡፡ በምርምሩ ለመሳተፍ ከመወሰንዎ በፊት ምርምሩን የሚመራው ሰው ስለፕሮጀክቱ ማብራሪያ ሊሰጥዎ ይገባል፡፡ እባክዎ ከመረጃው ወረቀት ወይም ከተደረገልዎት ገለጻ የመነጨ ጥያቄ ካለዎት በምርምሩ ለመሳተፍ ከመወሰንዎ በፊት ጥናቱን የሚያካሂደውን ግለሰብ ይጠይቁ በእጅዎ ይኖር ዘንድ እና በፈለጉ ጊዜ እንዲያመሳክሩበት የዚህ የስምምነት ቅጽ ግልባጭ ይሰጥዎታል፡፡

- በማንኛውም ጊዜ በምርምሩ ላለመሳተፍ ከወሰንኩኝ ለምርምሩ ለሚያካሂዱት ወይም ወኪሎቻቸው ማሳወቅ እንደምችልና ምንም ምክንያት ሳላቀርብ ከምርምሩ እራሴን ላገል እንደምችል ተረድቻለሁ፡፡ ከዚህም ባሻገር ጥናቱ እስኪታተም ድረስ የሰጠሁትን ቅጽ መረጃዎች ማውጣት እንደምችል ተረድቻለሁ፡፡
- የሰጠሁት የግል መረጃ ስለተገለፀልኝ አላማ ጥቅም ላይ ይውል ዘንድ ተስማምቻለሁ፡፡
- በጥልቀት ቃለ መጠይቁን ለመስጠት ከተመረጥኩ ቃለ መጠይቁ በመቅረጸ ድምጽ ይቀዳ ዘንድ እስማማለሁ፡፡
- የሰጡን መረጃ እንደ ሪፖርት ይታተማል፡፡ የሚሰጡን መረጃ ሚስጥራዊነት እንደሚጠበቅና ከሚወጡትም ሪፖርቶች ማንነቶን ለማወቅ እንደማይቻል ልናረጋግጥ እንወዳለን፡፡
- የምርምር ቡድኑ ቅድመ መረጃውን ለወደፊት ምርምር ሊጠቀም እንደሚችል እስማማለሁ፡፡
- የተሳታፊው መግለጫ__________________________________________

እኔ ________________________________የተባልኩኝ ግለሰብ ከላይ የተጠቀሰው የምርምር ፕሮጀክት በበቂ ሁኔታ ተብራርቶልኝ በጥናቱ ለመሳተፍ ተስማምቻለሁ፡፡ ከላይ የተጻፉትን ማሳሰቢያዎች አና ስለፕሮጀክቱ የሚገልጽ የመረጃ ወረቀት አንብቤ ጥናቱ የሚያካትተውን ተረድቻለሁ፡፡

ፊርማ ____________ ቀን ____________

**የምስክር ቃል (ተሳታፊው ያልተማረ ከሆነ)**

እኔ ________________________________ የተባልኩኝ ግለሰብ ከላይ የተጠቀሰው የምርምር ፕሮጀክት በበቂ ሁኔታ ለ _____________________ ተብራርቶላቸው በምርምሩ ለመሳተፍ ተስማምተዋል፡፡ ከላይ የተጻፉ ማሳሰቢያዎች እና ስለፕሮጀክቱ የሚገልጽ የመረጃ ወረቀት የተነበበላቸው ሲሆን ጥናቱ የሚያካትታቸውንም ጉዳዮች ተረድተዋል፡፡

ፊርማ ____________ ቀን ____________

**የቃለ መጠይቅ አቅራቢ ቃል፡-**

እኔ__________________________________ የጥናቱን ምንነት የሚፈልጋቸውን ነገሮችና በጥናቱ መሳተፍ ሊያከትል የሚችለውን ችግር (አግባብ ካለው) የሚችለውን ጉዳዮች( አስፈላጊ ሲሆን ) ለተሳታፊው በጥንቃቄ አብራርቻለሁ፡፡

ፊርማ ____________ ቀን ____________

# APPENDIX 6: Instruments Amharic

## General information

**አጠቃላይ መረጃ**

| 101 | ዕድሜዎ ስንት ነው? | /🖎_______ዓመት/ |  |
| --- | --- | --- | --- |
| 102 | መጀመሪያ **ጋብቻ ሲመሰርቱ** ዕድሜዎ ስንት ዓመት ነበር? | /🖎 _____/ ዓመት | AGEM |
| 103 | **የጋብቻ ሁኔታ**  [1] ያላገባች [2] በትዳር ላይ  [3] በፍቺ የተለየያች [4] በሞት የተለያየች  [5] ያለ ህጋዊ ጋብቻ አብራ የምትኖር [6] ያገባች ግን በሥራ ምክንያት አብራ የማትኖር | | MARIT |
| 104 | **ሐይማኖትዎ?**  [1] ኦርቶዶክስ ክርስቲያን [2] እስልምና [3] ፕሮቴስታንት  [4] ካቶሊክ [9] ሌላ 🖎 ……………………………. | | RELIG |
| 105 | **ብሔር** (ብሔርዎ ምንድን ነው?)  [1] ጉራጌ [2] ኦሮሞ [3] አማራ [4] ሌላ 🖎 _______ | | ETHN |

Socio-economic information

| 106 | **የትምህርት ሁኔታ** (የትምህርት ደረጃዎ ምንድን ነው)?  [1] ምንም አልተማርኩም  [2] ማንበብና መፃፍ እችላለሁ ግን መደበኛ ትምህርት የለኝም(ለምሳሌ፣ መሰረተ ትምህርት የተማሩ)  [3] 1ኛ-4ኛ ክፍል [4] 5ኛ-8ኛ ክፍል  [5] 9ኛ-10ኛ ክፍል [6] 11ኛ-12ኛ ክፍል  [7] ሰርተፍኬት [8] ዲፕሎማ  [9] የመጀመሪያ ዲግሪ [10] ማስትርስ እና ከዚያ በላይ | EDUCM |
| --- | --- | --- |
| 107 | **ስራዎ** (በዋናነት ገቢ የሚያገኙበት ወይም ጊዜዎን የሚያሳልፉበት ስራ) ምንድንነው?  [1] የቤት እመቤት [2] ወዝ አደር/ የጉልበት ሥራ [3] አርሶ አደር/ግብርና  [4] የግል ወይም የመንግስት ተቀጣሪ [5] ተማሪ [6] ነጋዴ  [7] ስራ አጥ [8] ጡረተኛ [97] ሌላ [ይገለፅ] /🖎 _____/ | OCCM |

| 108 | **የባለቤትዎ** **ሥራ** ምንድነው?  [1] ስራ አጥ [2] ወዝ አደር/ የጉልበት ሥራ [3] አርሶ አደር  [4] ነጋዴ [5] ተማሪ [6] የመንግስት/የግል ሰራተኛ  [7] ጡረተኛ [97] ሌላ [ይገለፅ] /🖎 __________________/ | OCCH |
| --- | --- | --- |

| 308 | **የባለቤትዎ የትምህርት ደረጃ** (የባለቤትዎ የትምህርት ደረጃ ምንድን ነው?)  [1] ምንም አልተማሩም [2] ያለመደበኛ ትምህርት ማንበብና መፃፍ ይችላሉ  [3] 1ኛ-4ኛ ክፍል [4] 5ኛ-8ኛ ክፍል  [5] 9ኛ-10ኛ ክፍል [6] 11ኛ-12ኛ ክፍል  [7] ሰርተፍኬት [8] ዲፕሎማ  [9] የመጀመሪያ ዲግሪ [10] ማስተርስ እና ከዚያ በላይ | EDUCH |
| --- | --- | --- |
| 309 | ጠቅላላ የቤተሰብ ብዛት ስንት ነው? /🖎 __________________/ | FAMS |
| 310 | ስንት ጥማድ የእርሻ ወይም የሰብል መሬት አለዎት? 🖎 /__________ / | MASA |
| 311 | በአካባቢዉ ካሉ ሌሎች ቤተሰቦች ጋር ሲነፃጸር በሃብት ደረጃ የራስዎን ቤተሰብ የት ያስቀምጡታል**?**  [1] ዝቅተኛ [2] መካከለኛ [3] ጥሩ ኑሮ ያለው | WELZR |

| 312 | **የገቢ ሁኔታ** (የቤተሰብዎ የቀን/የወር/የዓመት ገቢ ምን ያህል ነው?)  **ማስታዎሻ**፡ ***ለነጋዴ/ለቀን ሰራተኛ የቀን ገቢ፤ ለተቀጣሪ የወር ገቢ እን ለገበሬ የዓመት ገቢ ይጠየቅ፡፡*** | | INCOM |
| --- | --- | --- | --- |
|  | **ለነጋዴ/ለወዝ አደር፡** | የቀን ገቢ 🖎_________________________ ብር |  |
|  | **ለተቀጣሪ፡** | የወር ገቢ 🖎_________________________ ብር |  |
|  | **ለአርሶ አደር፡** | የዓመት ገቢ 🖎________________________ ብር |  |

## Patient Health Questionnaire (PHQ-9)

| ባለፉት ሁለት ሳምንታት ውስጥ የሚከተሉት ምልክቶች እያንዳንዳቸው ለስንት ቀናት ተከስተው ነበር? | | |
| --- | --- | --- |
| 901 | **ባለፉት ሁለት ሳምንታት ውስጥ** የእለት ተእለት ተግባርዎን ለማከናወን (ለመስራት) ያለዎት ተነሳሽነት ለምን ያህል ቀን ቀንሶ ነበር;  *[0] በፍፁም [1] ከ7 ቀናት ያነሰ [2] ከ7 ቀናት በላይ [3] ከሞላ ጎደል በየቀኑ* | PHQ1 |
| 902 | **ባለፉት ሁለት ሳምንታት ውስጥ** የመከፋት፣ የመደበት ወይም ተስፋ የመቁረጥ ስሜት ለምን ያህል ቀን ይሰማዎ ነበር;  *[0] በፍፁም [1] ከ7 ቀናት ያነሰ [2] ከ7 ቀናት በላይ [3] ከሞላ ጎደል በየቀኑ* | PHQ2 |
| 903 | **ባለፉት ሁለት ሳምንታት ውስጥ** ለምን ያህል ቀን እንቅልፍ አልወስድዎ ብሎ ወይም በደንብ መተኛት አቅቶዎት ወይም እንቅልፍ እየበዛብዎት ይቸገሩ ነበር;  *[0] በፍፁም [1] ከ7 ቀናት ያነሰ [2] ከ7 ቀናት በላይ [3] ከሞላ ጎደል በየቀኑ* | PHQ3 |
| 904 | **ባለፉት ሁለት ሳምንታት ውስጥ** ለምን ያህል ቀን የድካም ወይም የአቅም ማነስ ስሜት ይሰማዎት ነበር;  *[0] በፍፁም [1] ከ7 ቀናት ያነሰ [2] ከ7 ቀናት በላይ [3] ከሞላ ጎደል በየቀኑ* | PHQ4 |
| 905 | **ባለፉት ሁለት ሳምንታት ውስጥ** ለምን ያህል ቀን የምግብ ፍላጎትዎ ከተለመደዉ በላይ ጨምሮ ወይም ቀንሶብዎት ነበር;  *[0] በፍፁም [1] ከ7 ቀናት ያነሰ [2] ከ7 ቀናት በላይ [3] ከሞላ ጎደል በየቀኑ* | PHQ5 |
| 906 | **ባለፉት ሁለት ሳምንታት ውስጥ** ለምን ያህል ቀን ራስዎን የመጥላት ወይም ዋጋ የለኝም የማለት ወይም ራሴንም ሆነ ቤተሰቤን አሳዝኛለሁ/አሳፍሬያለሁ/ የሚል ስሜት ተሰምቶዎት ነበር;  *[0] በፍፁም [1] ከ7 ቀናት ያነሰ [2] ከ7 ቀናት በላይ [3] ከሞላ ጎደል በየቀኑ* | PHQ6 |
| 907 | **ባለፉት ሁለት ሳምንታት ውስጥ** ለምን ያህል ቀን በሚሰሩት ስራ ላይ ሃሳብዎን መሰብሰብ/ልብ የማለት ችግር (ለምሳሌ፡ ከሰዎች ጋር ሲጨዋወቱ ትኩረት ሰጥቶ ማዳመጥ) አስቸግሮዎት ነበር;  *[0] በፍፁም [1] ከ7 ቀናት ያነሰ [2] ከ7 ቀናት በላይ [3] ከሞላ ጎደል በየቀኑ* | PHQ7 |
| 908 | **ባለፉት ሁለት ሳምንታት ውስጥ** ለምን ያህል ቀን ለሌሎች ሰዎች እስከሚታወቅ ድረስ በእንቅስቃሴዎ ወይም በንግግርዎ በጣም ቀስ ብለዉ ወይም  በተቃራኒዉ መረጋጋት አቅቶዎት፣ አንድ ቦታ አርፎ መቀመጥ ወይም መቆም እስከማይችሉ ሆነዉ ነበር;  *[0] በፍፁም [1] ከ7 ቀናት ያነሰ [2] ከ7 ቀናት በላይ [3] ከሞላ ጎደል በየቀኑ* | PHQ8 |
| 909 | **ባለፉት ሁለት ሳምንታት ውስጥ** ብሞት ይሻላል ብለዉ አስበዉ ወይም ራስዎን በሆነ መንገድ ሊጎዱ አስበዉ ነበር;  *[0] በፍፁም [1] ከ7 ቀናት ያነሰ [2] ከ7 ቀናት በላይ [3] ከሞላ ጎደል በየቀኑ* | PHQ9 |
| 910 | ባለፉት ሁለት ሳምንታት በእነዚህ ከላይ ባጋጠሞዎት ችግርች ምክንያት ምን ያህል ቤት ውስጥ ስራ ለመስራት ወይም ራስዎን ለመቆጣጠር ወይም ማህበራዊ ጉዳዮችን ለማከናወን ተቸግረዋል፡፡  [0› ምንም ችግር የለም [1› የተወሰነ አስቸግሮኝ ነበር  [2› በጣም አስቸግሮኝ ነበር [3] እጅግ በጣም አስቸግሮኝ ነበር | PHQT |

## Intimate Partner Violence Measures

### Non-graphic (IPV)

በትዳር ምንም ያህል መዋደድ/መፋቀር ቢኖርም አልፎ አልፎ ግን አለመስማማት አይጠፋም፡፡ ስለሆነም እርስዎ ከባለቤትዎ ጋር ባልተስማሙ ጊዜ የሚሰማዎትን **ስሜት** ከዚህ በታች እጠይቅዎታለሁ፡፡

| 401 | ከባለቤትዎ ጋር ባልተስማሙ ጊዜ ችግሩን ለመፍታት ምን ያህል ያስቸግርዎታል?  [0] ምንም ችግር የለም [1] የተወሰነ ችግር አለ [2] በጣም ችግር አለ | **IPV1** |
| --- | --- | --- |
| 402 | በአጠቃላይ ከባለቤትዎ ጋር ያለዎት ቤተሰባዊ/ማህበራዊ/ ግንኙነት መሻከር እርስዎን ምን ያህል ያስጨንቅዎታል?  [0] ምንም አያስጨንቅም [1] የተወሰነ ያስጨንቃል [2] በጣም ያስጨንቃል | **IPV2** |
| 403 | ባለቤትዎ ለእርስዎና ለልጆችዎ የሚያደርጉትን እንክብካቤ እንዴት ይገልጹታል?  [0] ሁልጊዜ ጥሩ ነው [1] ብዙ ጊዜ ጥሩ ነው [2] ጥሩ ወይም መጥፎ የሚባል አይደለም  [3] ብዙ ጊዜ ጥሩ አይደለም [4] በፍጹም ጥሩ አይደለም | **IPV3** |
| 404 | አሁን ከባለቤትዎ ጋር ባለዎት ቤተሰባዊ/ማህበራዊ/ ግንኙነት ምን ያህል ሥጋት ይሰማዎታል?  [0] ሁልጊዜ ከሥጋት ነፃ ነኝ [1] ብዙ ጊዜ ከሥጋት ነፃ ነኝ  [2] አስጊ ነው ወይም አይደለም ለማለት ያስቸግራል [3] ብዙ ጊዜ የሰጋኛል  [4] ሁልጊዜ ያሰጋኛል | **IPV4** |
| 405 | ከባለቤትዎ ወይም ሌሎች የቤተሰብ አባላት ጋር ያለዎት ማህበራዊ ግንኙነት ምን ያህል ያሰጋዎታል?  [0] ሁልጊዜ ከሥጋት ነፃ ነኝ [1] ብዙ ጊዜ ከሥጋት ነፃ ነኝ  [2] አስጊ ነው ወይም አይደለም ለማለት ያስቸግራል [3] ብዙ ጊዜ የሰጋኛል  [4] ሁልጊዜ ያሰጋኛል | **IPV5** |

### 1.3.2. WHO IPV Questionnaire

**
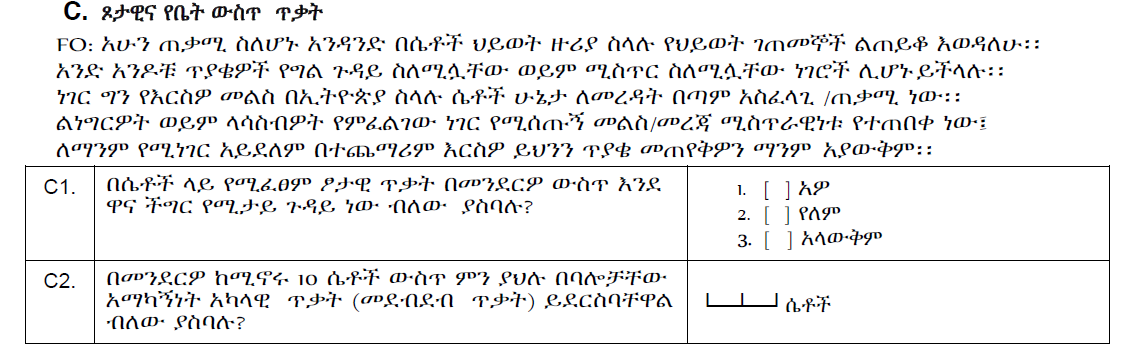
**


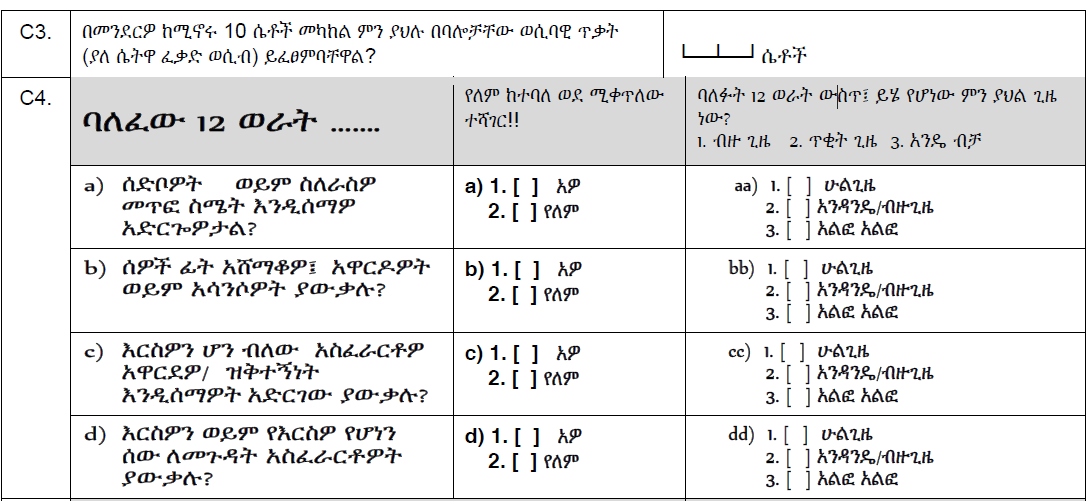


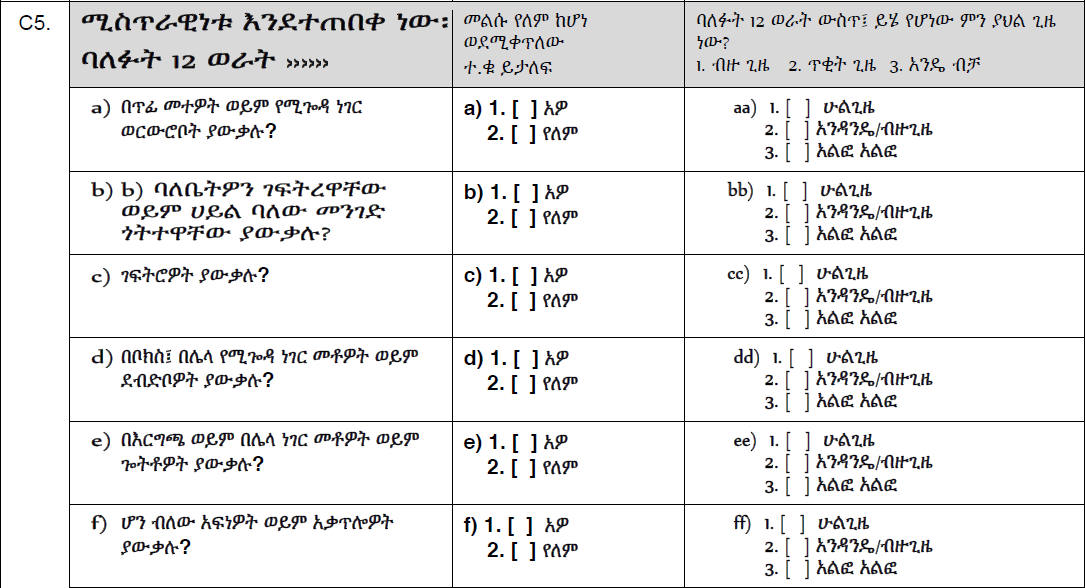


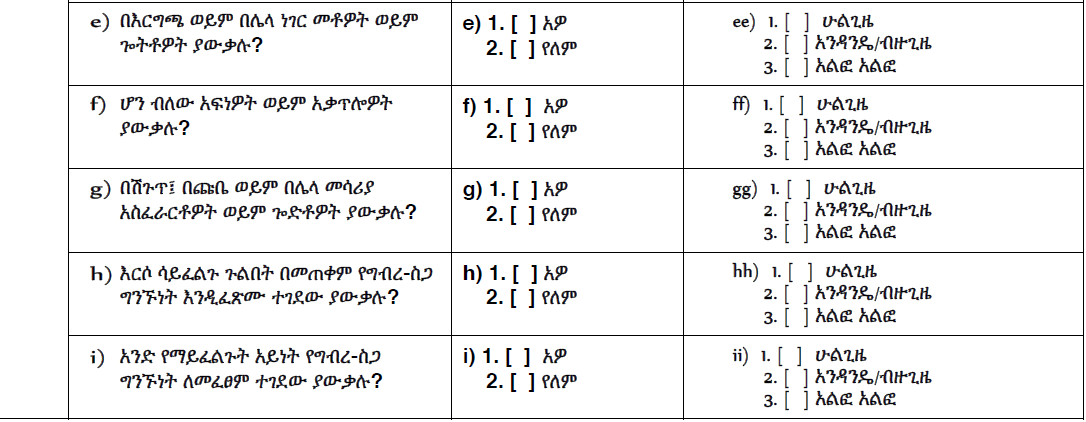


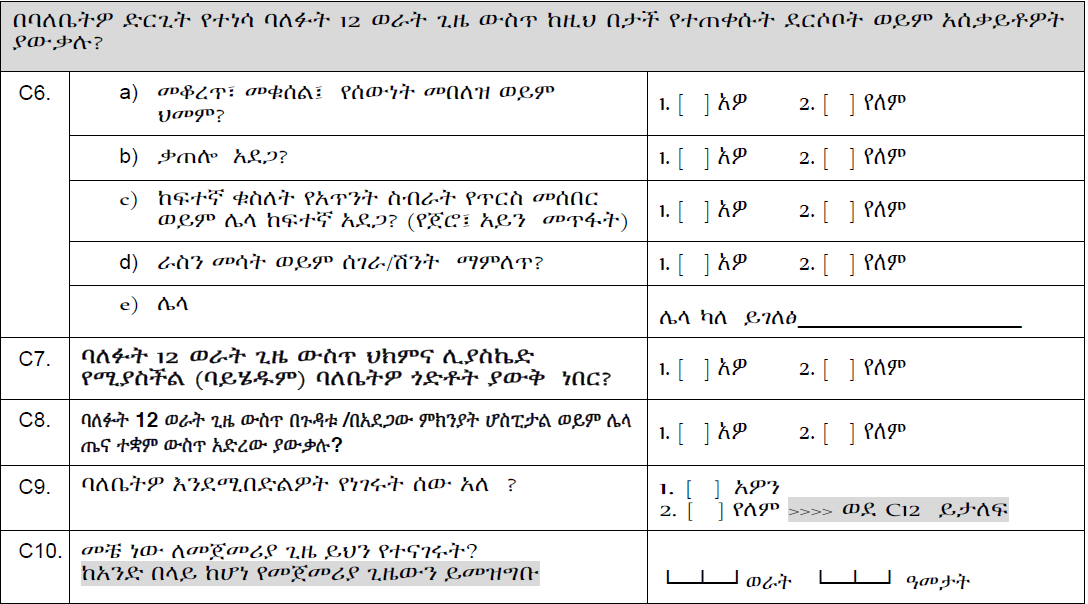


## PCL-5

ከዚህ በታች የተገለፁት የችግር ዝርዝሮች ሰዎች በጣም አስጨናቂ ክስተቶች ሲያጋጥሙዋቸው የሚያሳዩት ሊሆን ይችላል፡፡ እባክዎትን ከዚህ በታች የተዘረዘሩትን ችግሮች ካነበቡ በኋላ በችግሩ ላለፉት ለአንድ ወር ለምን ያህል እንደተረበሹ የሚመለክተውን ከበስተቀኝ ከተገለፁት ምርጫዎች አንዱን ይምረጡ ፡፡

|  | **ባለፉት አንድ ወር ምን ያህል ተረብሸው ነበር በ……..** | **በፍጹም የለም** | **በጥቂቱ** | **መሀከለኛ** | **በብዛት** | **እጅግ በጣም** |
| --- | --- | --- | --- | --- | --- | --- |
| 1 | በተደጋጋሚ የሚመጡ፤ የሚረብሹ፤ የማይፈለጉ የአስጨናቂ አጋጣሚ ትዝታዎች | 0 | 1 | 2 | 3 | 4 |
| 2 | በተዳዳጋሚ የሚመጡ በሚረብሹ የአስጨናቂው አጋጣሚ አይነት ህልሞች |  |  |  |  |  |
| 3 | ድንገት በሚመጣ የአስጨናቂው አጋጣሚ እንደገና እንደሚከሰት በሚመስል አይነት ስሜት ( ነገሩን አንደገና መከሰት) |  |  |  |  |  |
| 4 | የአስጨናቂውን አጋጣሚ የሚያስታውስ ነገር ሲኖር በሚያሰከፋ ስሜት |  |  |  |  |  |
| 5 | የአስጨናቂውን አጋጣሚ ጊዜ የሚያስታውስ ነገር ሲኖር ሰውነት ላይ በሚፈጠሩ ሂደቶች (ለምሳሌ ልብ በጣም መምታት፤ለመንተፈስ መቸገር፤ በማላብ) |  |  |  |  |  |
| 6 | የአስጨናቂው አጋጣሚ ጋር የተያያዙ ትዝታዎች፤ሀሳብ ወይም ስሜቶችን በመሸሽ |  |  |  |  |  |
| 7 | የአስጨናቂው አጋጣሚ የሚያስታውሱ ውጫዊ ነገሮችን በመሸሽ |  |  |  |  |  |
| 8 | በጣም አስፈላጊ የአስጨናቂው አጋጣሚ ክፍል ለማስታወስ በመቸገር |  |  |  |  |  |
| 9 | ስለራስህ/ሽ ወይም ስለ ሰዎች ወይም ስለአለም መጥፎ በሆነ እምነት (ለምሳሌ "እኔ መጥፎ ነኝ፤ እኔ ላይ አንድ የተሳሳተ ነገር አለ" በሚል ሀሳብ  ማንም ሰው አይታመንም፤አለም በጠቅላላ አደገኛ ናት |  |  |  |  |  |
| 10 | ራስን ወይም ሌላ ሰውን ለአስጨናቃው አጋጣሚ መከሰት መውቀስ |  |  |  |  |  |
| 11 | ሀይለኛ መጥፎ በሆኑ ስሜቶች እንደ ፍርሀት፤መሳቀቅ፤ ንዴት ፤ፀፀት፤ ወይም ሀፍረት |  |  |  |  |  |
| 12 | ከዚህ በፊት ከሚያስደስቱ እንቅስቃሴዎች ላይ ፍላጎት ማጣት |  |  |  |  |  |
| 13 | ከሰዎች መራቅ ወይም የመዘጋት ስሜት |  |  |  |  |  |
| 14 | ቅን ስሜቶችን ለማጣጣም መቸገር (ለምሳሌ የደስተኛ ስሜትን ለመሰማት ወይም ለሌሎች ቅርብ ሰዎች የፍቅር ስሜትን ለመሰማት |  |  |  |  |  |
| 15 | የመነጫነጭ ባህሪ፤ የንዴት መገንፈል፤ ወይም የአጥቂነት ድርጊቶችን |  |  |  |  |  |
| 16 | ብዙ ለአደጋ አጋላጭ ወይም ጉዳት ሊያደርሱ የሚችሉ ነገሮችን ማድረግ |  |  |  |  |  |
| 17 | በጣም ንቁ ወይም አስተዋይ ወይም ጫፍ ላይ መሆን |  |  |  |  |  |
| 18 | በቀላሉ መደንገጥ ወይም መበርገግ |  |  |  |  |  |
| 19 | ትኩረትን ለመሰብሰብ መቸገር |  |  |  |  |  |
| 20 | እንቅልፍ ለመውሰድ ወይም ለመተኛት መቸገር |  |  |  |  |  |
|  | ድምር |  |  |  |  |  |

## GAD-7

| ባለፉትሁለትሳምንታትውስጥበሚከተሉትችግሮችምክንያትምንያህልተጨንቀውነበር? | | | | |
| --- | --- | --- | --- | --- |
| 1 | የመርበድበድስሜትወይምየጭንቀትስሜት | በጭራሽ | 1 |  |
|  |  | ብዙጊዜ | 2 |  |
|  |  | ከ 7 ቀናትበላይ | 3 |  |
|  |  | ሁልጊዜ/በየእለቱአካባቢ | 4 |  |
| 2 | ስለሚያሳስብዎት/ሚያስጨንቅዎትነገርማስቆምወይምመቆጣጠርአለመቻል | በጭራሽ | 1 |  |
|  |  | ብዙጊዜ | 2 |  |
|  |  | ከ7 ቀናትበላይ | 3 |  |
|  |  | ሁልጊዜ/በየእለቱአካባቢ | 4 |  |
| 3 | ስለተለያዩነገሮችከመጠንበላይማሰብ/መጨነቅ | በጭራሽ | 1 |  |
|  |  | ብዙጊዜ | 2 |  |
|  |  | ከ7 ቀናትበላይ | 3 |  |
|  |  | ሁልጊዜ/በየእለቱአካባቢ | 4 |  |
| 4 | ዘናማለት/መዝናናትአለመቻል | በጭራሽ | 1 |  |
|  |  | ብዙጊዜ | 2 |  |
|  |  | ከ7 ቀናትበላይ | 3 |  |
|  |  | ሁልጊዜ/በየእለቱአካባቢ | 4 |  |
| 5 | መቁነጥነጥናተረጋግቶመቀመጥአለመቻል | በጭራሽ | 1 |  |
|  |  | ብዙጊዜ | 2 |  |
|  |  | ከ7 ቀናትበላይ | 3 |  |
|  |  | ሁልጊዜ/በየእለቱአካባቢ | 4 |  |
| 6 | በቀላሉመናደድወይቶሎቶሎመናድድ | በጭራሽ | 1 |  |
|  |  | ብዙጊዜ | 2 |  |
|  |  | ከ7 ቀናትበላይ | 3 |  |
|  |  | ሁልጊዜ/በየእለቱአካባቢ | 4 |  |
| 7 | የሆነየከፋነገርሊከሰትእንደሚችልመፍራት | በጭራሽ | 1 |  |
|  |  | ብዙጊዜ | 2 |  |
|  |  | ከ7 ቀናትበላይ | 3 |  |
|  |  | ሁልጊዜ/በየእለቱአካባቢ | 4 |  |

## 1.6. WHODAS-12

ይህ መጠይቅ ሰዎች በጤና እክል ምክንያት ስለሚኖራቸዉ ችግር ይሆናል፡፡ የጤና እክል ስል በሽታ ወይም ህመም፣ሌሎች ለአጭር ወይም ለረጅም ጊዜ የሚቆዩ የጤና ችግሮች፣ ጉዳቶች፣ የአእምሮ ወይም የመንፈስ መታወክ፣ እንዲሁም ከመጠጥ እና ከእጽ ጋር የተገናኙ ችግሮችን ይሆናል፡፡ ቃለ-መጠይቆቹን ሲመልሱ **ሁሉንም የጤና ችግሮችዎን** እንዲያስቡ እፈልጋለሁ፡፡ ***ጥያቄዎቹን ሲመልሱ ያለፉትን 30 ቀናት እያስታወሱ ይሁን፡፡***

| 1 | ባለፉት 30 ቀናት ውስጥ የጤናዎትን አጠቃላይ ሁኔታ እንዴት ይመዝኑታል?  [1 ]በጣም ጥሩ [2 ]ጥሩ [3 ] መካከለኛ [4 ]የከፋ [5] በጣም የከፋ | | | | | | ALL |
| --- | --- | --- | --- | --- | --- | --- | --- |
| ***ጥያቄዎቹን ሲመልሱ እነዚህን 5 የችግር ደረጃዎች ይጠቀሙ፡፡***  **ምንም ችግር የለም = [1 ] አነስተኛ ችግር = [2 ] መካከለኛ ችግር = [3 ] ከፍተኛ ችግር = [4 ] በጣም ከፍተኛ ችግር = [5]**  **ባለፉት 30 ቀናት የሚከተሉትን ሲያደርጉ ምን ያህል ይቸግርዎት ነበር?** | | | | | | | |
| 2 | **ረዘም ላለ ጊዜ መቆም** ምን ያህል ይቸግርዎት ነበር? ለምሳሌ ግማሽ ሰዓት | [1 ] | [2 ] | [3 ] | [4 ] | [5 ] | STAND |
| 3 | የቤት ዉስጥ **ሃላፊነቶችን መወጣት** ምን ያህል ይቸግርዎት ነበር? | [1 ] | [2 ] | [3 ] | [4 ] | [5 ] | RESP |
| 4 | **አዲስ ነገር ወይም ስራ**  ለመማር **ይቸገሩ ነበር**? (ለምሳሌ የእርሻ ስራ፣ ባልትና፣ የእጅ ስራ፣ የሞባይል አጠቃቀም፣ ወዘተ…) | [1 ] | [2 ] | [3 ] | [4 ] | [5 ] | LEARN |
| 5 | **በማህበራዊ እንቅስቃሴ** ዉስጥ (ለምሳሌ፡- ዓመት በዓል፣ ድግስ፣ ለቅሶ፣ እድር፣ ሊቃ…ወዘተ) ልክ እንደ ሌላዉ ሰዉ መሳተፍ ምን ያህል ይቸግርዎት ነበር? | [1 ] | [2 ] | [3 ] | [4 ] | [5 ] | SOCIAL |
| 6 | የርስዎ የጤና ችግር ምን ያህል **ስሜትዎት ላይ ተጽእኖ** አደረገብዎት? | [1 ] | [2 ] | [3 ] | [4 ] | [5 ] | EMOT |
|  | **ባለፉት 30 ቀናት የሚከተሉትን ሲያደርጉ ምን ያህል ይቸግርዎት ነበር?** | | | | | | |
| 7 | በሚሰሩት ስራ ላይ **ሀሳብዎን ለጥቂት ጊዜ (ለ10 ደቂቃ) ያህል መሰብሰብ** ይቸገሩ ነበር? | [1 ] | [2 ] | [3 ] | [4 ] | [5 ] | CONC |
| 8 | **ረዘም ያለ ርቀት ለመጓዝ**  ምን ያህል ይቸገሩ ነበር? ምሳሌ የሩብ ሰአት መንገድ (1ኪ.ሜትር) | [1 ] | [2 ] | [3 ] | [4 ] | [5 ] | WALK |
| 9 | **ሰዉነትዎን መታጠብ** ምን ያህል ይቸግርዎት ነበር? | [1 ] | [2 ] | [3 ] | [4 ] | [5 ] | SHOW |
| 10 | **ልብስዎትን መልበስ** ምን ያህል ይቸግርዎት ነበር? | [1 ] | [2 ] | [3 ] | [4 ] | [5 ] | DRESS |
| 11 | ከማያዉቁዋቸዉ ሰዎች ጋር ተግባብቶ **ጉዳይ መፈጸም** ምን ያህል ይቸግርዎት ነበር? | [1 ] | [2 ] | [3 ] | [4 ] | [5 ] | DEAL |
| 12 | **በጓደኝነት መቆየት** ምን ያህል ይቸግርዎት ነበር? | [1 ] | [2 ] | [3 ] | [4 ] | [5 ] | FRIEND |
| 13 | የዕለት ተዕለት **ስራዎን ወይም ትምህርትዎን ለማከናወን** ምን ያህል ይቸግርዎት ነበር? | [1 ] | [2 ] | [3 ] | [4 ] | [5 ] | DAILY |
| 14 | በአጠቃላይ እነዚህ ችግሮች በህይወትዎ ጣልቃ እየገቡ ምን ያህል አስቸገርዎት? | [1 ] | [2 ] | [3 ] | [4 ] | [5 ] | INTERF |

## Attitudes Towards Gender Roles (WHO Multi-Country Study)

በዚሁም ሆነ በሌላ ህብረተሰብ ዘንድ ሰዎች በአንድ ቤተሰብ ውስጥ ስለወንዶችና ስለሴቶች የባህሪ መልካምነት የተለያየ ሃሳብ አላቸው፡፡ አሁን ከዚህ በታች ዐረፍተ ነገሮቸን አነብሎታለሁ፡፡ እርስዎ ለዐረፍተ ነገሮቹ በአጠቃላይ መስማማት አለመስማማትዎን እንዲገልፁልኝ በአክብሮት እጠይቃለሁ፡፡ ትክክለኛ የሚባል ምላሸ የለውም፡፡

| 1 | መልካም ሚስት ባይስማማትም የባሏን ትዕዛዝ ታከብራለች፡፡ | | እስማማለሁ | 1 |
| --- | --- | --- | --- | --- |
|  |  |  | አልስማማም | 2 |
|  |  |  | አላወቅም | 8 |
| 2 | የቤተሰብ ችግር ለውይይት መቅረብ ያለበት ለቤተሰብ አባላት ብቻ ነው፡፡ | | እስማማለሁ | 1 |
|  |  |  | አልስማማም | 2 |
|  |  |  | አላወቅም | 8 |
| 3 | ወንድ ለሚስቱ አለቃዋ መሆኑን ማሳየት መቻል ያስፈልጋል፡፡ | | እስማማለሁ | 1 |
|  |  |  | አልስማማም | 2 |
|  |  |  | አላወቅም | 8 |
| 4 | ሴት ልጅ ባሏ ባይፈቅድላትም የራሷን ጓደኞች መምረጥ መቻል አለባት፡፡ | | እስማማለሁ | 1 |
|  |  |  | አልስማማም | 2 |
|  |  |  | አላወቅም | 8 |
| 5 | ሚስት ባትወድም ከባሏ ጋር ፆታዊ ግንኙነት ማድረግ ግዴታዋ ነው፡፡ | | እስማማለሁ | 1 |
|  |  |  | አልስማማም | 2 |
|  |  |  | አላወቅም | 8 |
| 6 | አንድ ወንድ ሚስቱን የሚያንገላታ ከሆነ ከቤተሰብ ወጭ የሆኑ ሌሎች ሰዎች መገላገል አለባቸው፡፡ | | እስማማለሁ | 1 |
|  |  |  | አልስማማም | 2 |
|  |  |  | አላወቅም | 8 |
| 7 | በእርስዎ ግምት ባል ሚስቱን ለመምታት በቂ ምክንያት የሚኖረው፡- | 1. ሚስት ባሏን በሚያረካ ሁኔታ የቤት ውስጥ ስራዎችዋን የማታከናውን ከሆነ ነው፡፡ | እስማማለሁ | 1 |
|  |  |  | አልስማማም | 2 |
|  |  |  | አላወቅም | 8 |
|  |  | 1. የባሏን ትዕዛዝ የማታከብር ከሆነ ነው፡፡ | እስማማለሁ | 1 |
|  |  |  | አልስማማም | 2 |
|  |  |  | አላወቅም | 8 |
|  |  | 1. ከእሱ ጋር ፆታዊ ግንኙነት ለማድረግ የምትቃወም/የማትፈልግ ከሆነ ነው፡፡ | እስማማለሁ | 1 |
|  |  |  | አልስማማም | 2 |
|  |  |  | አላወቅም | 8 |
|  |  | 1. እሱ ሌሎች የሴት ጓደኞች እንዳሉት የምትጠይቀው ከሆነ ነው፡፡ | እስማማለሁ | 1 |
|  |  |  | አልስማማም | 2 |
|  |  |  | አላወቅም | 8 |
|  |  | 1. እሱ በሴትነቷ አለመታመንን የሚጠራጠራት ከሆነ ነው፡፡ | እስማማለሁ | 1 |
|  |  |  | አልስማማም | 2 |
|  |  |  | አላወቅም | 8 |
|  |  | 1. በሴትነቷ አለመታመኗን ሲደርስባት ነው፡፡ | እስማማለሁ | 1 |
|  |  |  | አልስማማም | 2 |
|  |  |  | አላወቅም | 8 |
| 8 | በእርስዎ ግምት አንዲት ባለትዳር ከባሏ ጋር ፆታዊ ግንኙነት ማድረግን መቃወም የምትችለው፡- | 1. እሷ ካልፈለገች ነው፡፡ | እስማማለሁ | 1 |
|  |  |  | አልስማማም | 2 |
|  |  |  | አላወቅም | 8 |
|  |  | (b) ባሏ ጠቺ ከሆነ ነው፡፡ | እስማማለሁ | 1 |
|  |  |  | አልስማማም | 2 |
|  |  |  | አላወቅም | 8 |
|  |  | (c) ካመማት/ጤነኛ ካልሆነች ነው፡፡ | እስማማለሁ | 1 |
|  |  |  | አልስማማም | 2 |
|  |  |  | አላወቅም | 8 |
|  |  | (d) በአግባቡ የማይንከባከባት ከሆነ ነው፡፡ | እስማማለሁ | 1 |
|  |  |  | አልስማማም | 2 |
|  |  |  | አላወቅም | 8 |

## 1.8. Adapted self-efficacy scale

ከዚህ በታች ያሉት ዐረፍተ ነገሮች በያዙት ሃሳብ ያለዎትን የስምምነት መጠን ይግለፁ፡፡

1. ችግሮችን ከባለቤቴ ጋር በመነጋገር ባለኝ ችሎታ በራስ መተማመን ይሰማኛል፡፡

| **በጣም አልስማማም** | **አልስማማም** | **እርግጠኛ አይደለሁም** | **እስማማለሁ** | **በጣም እስማማለሁ** |
| --- | --- | --- | --- | --- |
| **0** | **1** | **2** | **3** | **4** |

1. ችግሮች ሲኖሩ ለባለቤቴ የመፍትሄ ሃሳብ ለመጠቆም ባለኝ ችሎታ በራስ መተማመን ይሰማኛል፡፡

| **በጣም አልስማማም** | **አልስማማም** | **እርግጠኛ አይደለሁም** | **እስማማለሁ** | **በጣም እስማማለሁ** |
| --- | --- | --- | --- | --- |
| **0** | **1** | **2** | **3** | **4** |

1. ባለቤቴን ሳላስከፋ የቤት ውስጥ ችግሮችን መወያየት እንደምችል በራስ መተማመን ይሰማኛል፡፡

| **በጣም አልስማማም** | **አልስማማም** | **እርግጠኛ አይደለሁም** | **እስማማለሁ** | **በጣም እስማማለሁ** |
| --- | --- | --- | --- | --- |
| **0** | **1** | **2** | **3** | **4** |

1. ችግሮች ሲኖሩ ባለቤቴን ሳላስከፋ የመፍትሄ ሃሳብ መጠቆም እንደምችል በራስ መተማመን ይሰማኛል፡፡

| **በጣም አልስማማም** | **አልስማማም** | **እርግጠኛ አይደለሁም** | **እስማማለሁ** | **በጣም እስማማለሁ** |
| --- | --- | --- | --- | --- |
| **0** | **1** | **2** | **3** | **4** |

1. ባለቤቴ የሆነ ነገር እንዲሰራ ከፈልግሁ ማሳመን እንደምችል በራስ መተማመን ይሰማኛል፡፡

| **በጣም አልስማማም** | **አልስማማም** | **እርግጠኛ አይደለሁም** | **እስማማለሁ** | **በጣም እስማማለሁ** |
| --- | --- | --- | --- | --- |
| **0** | **1** | **2** | **3** | **4** |

## Translated Multicultural Mastery Scale

ከዚህ በታች ላሉት ዐረፍተ ነገሮች ያለዎትን የስምምነት መጠን ይግለፁ፡፡

1. ከጎሮቤቶቼ ጋር በመወያየት ብዙ ችግሮቼን መቅረፍ እችላለሁ፡፡

|  |  |
| --- | --- |
|  |  |

በጭራሽ በመጠኑ በጣም

1. ከቤተሰቦቼ ጋር በመወያየት ብዙ ችግሮቼን መቅረፍ እችላለሁ፡፡

|  |  |
| --- | --- |
|  |  |

በጭራሽ በመጠኑ በጣም

1. በጎሮቤቶቼ እገዛ ለህይወቴ አስፈላጊ የሆኑ ብዙ ነገሮችን መቀየር እችላለሁ፡፡

|  |  |
| --- | --- |
|  |  |

በጭራሽ በመጠኑ በጣም

1. በቤተሰቦቼ እገዛ ለህይወቴ አስፈላጊ የሆኑ ብዙ ነገሮችን መቀየር እችላለሁ፡፡

|  |  |
| --- | --- |
|  |  |

በጭራሽ በመጠኑ በጣም

1. ያሰብኩትን ነገር ማከናወን እችላለሁ ምክንያቱም ጎሮቤቶቼ ይደግፉኛል፡፡

|  |  |
| --- | --- |
|  |  |

በጭራሽ በመጠኑ በጣም

1. ያሰብኩትን ነገር ማከናወን እችላለሁ ምክንያቱም ቤተሰቦቼ ይደግፉኛል፡፡

|  |  |
| --- | --- |
|  |  |

በጭራሽ በመጠኑ በጣም

1. ብዙውን ጊዜ ምኞቴ/ፍላጎቴ ሊሳካ የሚችለው በጎሮቤቶቼ ድጋፍ ነው፡፡

|  |  |
| --- | --- |
|  |  |

በጭራሽ በመጠኑ በጣም

1. ብዙውን ጊዜ ምኞቴ/ፍላጎቴ ሊሳካ የሚችለው ቤተሰቦቼ ድጋፍ ነው፡፡

|  |  |
| --- | --- |
|  |  |

በጭራሽ በመጠኑ በጣም

1. እኔ የምፈልገውን ማግኘት የምችለው ጎሮቤቶቼ የሚፈልጉትን እንዲያገኙ በማገዝ ነው፡፡

|  |  |
| --- | --- |
|  |  |

Not at all

በጭራሽ Somewhat በመጠኑ በጣም lot

1. እኔ የምፈልገውን ማግኘት የምችለው ቤተሰቦቼ የሚፈልጉትን እንዲያገኙ በማገዝ ነው፡፡

|  |  |
| --- | --- |
|  |  |

በጭራሽ በመጠኑ በጣም

1. በአብዛኛው ያሉብኝን ችግሮች ራሴ መቅረፍ እችላለሁ፡፡

|  |  |
| --- | --- |
|  |  |

በጭራሽ በመጠኑ በጣም

1. በህይወቴ አስፈላጊ የሆኑ ብዙ ነገሮችን እንደገና መወሰን (መቀየር) እችላለሁ፡፡

|  |  |
| --- | --- |
|  |  |

በጭራሽ በመጠኑ በጣም

1. በጣም አስቸጋሪ ነገሮች ቢሆኑም በጥሩ ሁኔታ አከናውናለሁ፡፡

|  |  |
| --- | --- |
|  |  |

በጭራሽ በመጠኑ በጣም

1. በእኔ ላይ የሚከሰቱ ነገሮች ብዙ ጊዜ በእኔ ማንነት ላይ የተመሰረቱ ናቸው፡፡

|  |  |
| --- | --- |
|  |  |

በጭራሽ በመጠኑ በጣም

1. አእምሮየ ያመነውን ነገር ሁሉ መስራት እችላለሁ፡፡

|  |  |
| --- | --- |
|  |  |

በጭራሽ በመጠኑ በጣም

## Enhancing Assessment of Common Therapeutic factors (ENACT) rating scale

| **ቀን፡ ___ ___ / ___ ___ / ___ ___ ___**  ቀን ወር ዓ.ም |  |
| --- | --- |

የሚገመገው ሰው ስም: _____________________________ ዕድሜ: ______ ፆታ: ወ/ሴ

ቅፁን የሞላው ሰው ስም: ____________________________ የስራ መደብ: _________

ግምገማው የተካሄድበት ቦታ:__________________

| ከንግግር ባሻገር ያለ አግባብ እና ትኩረት ሰጥቶ እና ተገቢ የሆነ የሰውነት እንቅስቃሴ በማሳየት ለመግባባት መሞከር  _ _ ተግባራዊ አይደለም | | |
| --- | --- | --- |
| ሀ | መሻሻል ያስፈልገዋል | = ተገልጋዩን በአግባቡ በአይን አታይም ወይም ታፈጥባቸዋለች፤ ትቆጣለች፤ ትስቅባቸዋለች/ታፌዝባቸዋለች፤ ጀርባዋን ትሰጣቸዋለች፤ በተደጋጋሚ ንግግራቸውን ታቋርጣቸዋለች፤ በሽተኛውን ችላ ትለዋለች፤ ሳታስፈቅድ ተንቀሳቃሽ ስልክ ታናግራለች |
| ለ | በከፊል የተፈጸመ | = ትኩረትን ለመግለጽ በተደጋጋሚ የሰውነት መግባቢያን አትጠቀምም: አልፎ አልፎ የአይን ለአይን መተያየትን ትጠቀማለች፣ የተወሰኑ ስሜቶችን ብቻ ትገልጻለች፣ ማስመሰልን ታበዛለች |
| ሐ | በትክክል የተፈጸመ | = በውይይት ወቅት ተገቢ የሆነ የአይን ለአይን መተያየት ትጠቀማለች፤ ተገቢ ሲሆን ፈገግ ትላለች፤ ከህመምተኛው በተገቢ አቅጣጫ ትቀመጣለች፤ ፍላጎትን ለማሳየት ወደ ህምምተኛው ታዘምማለች፤ `እህ እና ሌሎች በውይይቱ ፍላጎትን የሚገልጹ ድምጾችን ትጠቀማለች |
| 2. በንግግር የመግባባት፤ ማብራሪያ የሚጋብዙ ጥያቄዎች መጠየቅ፤ ፍሬ ሀሳቡን መድገም እና ግልጽ ባልሆኑ ጉዳዮች ማብራሪያ መስጠት _ _ ተግባራዊ አይደለም | | |
| ሀ | መሻሻል ያስፈልገዋል | = ብዙውን ጊዜ የአይ ወይም አዎ ጥያቄዎችን ትጠቀማለች፡፡ ለምሳሌ “መድኃኒት ትውጣለህ?” |
| ለ | በከፊል የተፈጸመ | = ማብራሪያ የሚጋብዙ ጥያቄዎችን ትጠቀማለች፡፡ ነገር ግን ስለጉዳዩ በጥልቀት አትመረምርም ወይም ፍሬ ሀሳቡን በመድገም ተገልጋዩ ሀሳቡን እንዲያብላሉት አታደርግም |
| ሐ | በትክክል የተፈጸመ | = ማብራሪያ የሚጋብዙ ጥያቄዎችን ትጠቀማለች፤ ፍሬ ሀሳቡን በመድገም እና ሀሳቡን በማብራራት ለምሳሌ “ምን ተፈጠረ?”፣ “እስኪ ሌላም ካለ ንገረኝ”? የሚሉ ዓይነት ጥያቄዎች ታቀርባለች፡፡ |
| 3. ጥሩ አቀራረብ መፍጠር _ _ ተግባራዊ አይደለም | | |
| ሀ | መሻሻል ያስፈልገዋል | = ባለሙያዋ ተገልጋዩ ዘና እንዲሉ ለማድረግ በአክብሮት ለማስተናገድ አትሞክርም፡፡ |
| ለ | በከፊል የተፈጸመ | = ባለሙያዋ ተገልጋዩ ዘና እንዲሉ ለማድረግ አትሞክርም ነገር ግን በአክብሮትና በክብር ለማስተናገድ ትሞክራለች፡፡ |
| ሐ | በትክክል የተፈጸመ | = ባለሙያዋ ተገልጋዩ ዘና እንዲሉ ለማድረግ በአክብሮትና በክብር ለማስተናገድ ትሞክራለች፡፡ |
| 4. ጠለቅ ያለ ምርምር፣ ትንተናና እና ስሜቱ እንግዳ እንዳልሆነ ማስረዳት _ _ ተግባራዊ አይደለም | | |
| ሀ | መሻሻል ያስፈልገዋል | = ባለሙያዋ ስለ ተገልጋዩ ስሜት አትጠይቅም ወይም ባለሙያዋ ስለ ተገልጋዩ ስሜቶች ትችት ወይም ፍርድ አዘል አነጋገር ትናገራለች (ምሳሌ፡ “እንደዛ ሊሰማህ አይገባም” “እንደዛ ማሰብህ ወይም እንደዛ ያለውን ስሜትህን ማቆም አለብህ፡፡” |
| ለ | በከፊል የተፈጸመ | = ስለተገልጋዩ ስሜት ትጠይቃለች ነገር ግን ማንንም ሰው ሊገጥም እንደሚችል (የተለመደ እንደሆነ) አትነግራቸውም ወይም አታረጋግጥም ወይም ስለ ተገልጋዩ ስሜት በጥልቀት አትመረምርም (የአዎ/አይ ምላሽ የሚያስፈልጋቸውን ጥያቄዎችን ትጠይቃለች) |
| ሐ | በትክክል የተፈጸመ | = ባለሙያዋ አንድ በዚህ ሁኔታ ያለ በሽተኛ የሚሰማውን ስሜት የሚጠበቅና በተገልጋዩ ሁኔታ ውስጥ ላለ ሰው የሚገጥም እንደሆነ ታስረዳለች፡፡ |
| 5. ተገልጋዩ ያለበትን ሁኔታ መረዳት (Empathy)፤ ሞቅ ያለ አቀባበል እና ከልብ በመነጨ ስሜት የመመለስ ክህሎት  _ _ ተግባራዊ አይደለም | | |
| ሀ | መሻሻል ያስፈልገዋል | = ተገልጋዩ ለሚያቀርቡት ቅሬታና ስጋት በቁጣ ወይም በትችት ትመልሳለች፡፡ |
| ለ | በከፊል የተፈጸመ | = ባለሙያዋ አትቆጣም ወይም አትተችም ነገር ግን ተገልጋዩ ያሉበትን ሁኔታ መረዳቷን አትገልጽም ወይም ከነአካቴው ከቁምነገር አትቆጥረውም፡፡ |
| ሐ | በትክክል የተፈጸመ | = ባለሙያዋ የተገልጋዩን ሁኔታ እንደተረዳች በእውነተኛ እና ከልብ በሆነ ሁኔታ ማሳየት ችላለች፡፡ |
| 6. ማህበራዊና ስነልቦናዊ ችግሮች በህይወት፤ በስራና በዕለት ተዕለት እንቅስቃሴ ላይ ያላቸውንን ተጽዕኖ የመመርመር ክህሎት_ _ ተግባራዊ አይደለም | | |
| ሀ | መሻሻል ያስፈልገዋል | = ባለሙያዋ ተገልጋዩ ያላቸውን ጭንቀት፣ ስጋት፣ ሃሳብ እና ማህበራዊ ችግሮች በዕለት ተዕለት እንቅስቃሴያቸው ላይ የፈጠሩትን ተጽዕኖዎች አትጠይቅም፡፡ |
| ለ | በከፊል የተፈጸመ | = ባለሙያዋ ተገልጋዩን በዕለት ተዕለት እንቅስቃሴው ዙሪያ ትጠይቃለች ነገር ግን ከማህበራዊና ስነልቦናዊ ጉዳዮቹ ጋር አታያይዝም፡፡ |
| ሐ | በትክክል የተፈጸመ | = ባለሙያዋ በስራ ሁኔታና በማህበራዊና ስነልቦናዊ ችግሮች መካከል ያለውን ግንኙነት ትመረምራለች፡፡ |

| 7. ስለ ህመማቸውና ስለ ሌሎች ችግሮቻቸው መንስኤ የተገልጋዩንና ድጋፍ የሚሰጡትን ሰዎች አመለካከት በጥልቀት የመመርመር ክህሎት (የመንስኤ መግለጫ) _ _ ተግባራዊ አይደለም | | |
| --- | --- | --- |
| ሀ | መሻሻል ያስፈልገዋል | = ባለሙያዋ ተገልጋዩን የህመማቸውንና የሌሎች ችግሮቻቸው መንስኤ ምን ሊሆን እንደሚችል የሚያስቡትን አትጠይቅም፡፡ ወይም ለተገልጋዩ አስተያየት የራሷን ፍርድ ወይም ትችት ትሰጣለች (ለምሳሌ፡ “ጠንቋይ ለዚህ አይነት ችግሮች መንስኤ ሊሆን አይችልም፤ ይህ ኋላቀር ወይም የድንቁርና አስተሳሰብ ነው!) |
| ለ | በከፊል የተፈጸመ | = ባለሙያዋ ተገልጋዩን የህመማቸውንና የሌሎች ችግሮቻቸው መንስኤ ምን ሊሆን እንደሚችል የሚያስቡትን ትጠይቃለች፤ ነገር ግን ከቤተሰባቸውና ሌሎች ዘመድ ወዳጆቻቸው ጋር ተመሳሳይ መሆን አለመሆኑን አትመረምርም፡፡ |
| ሐ | በትክክል የተፈጸመ | = ባለሙያዋ ተገልጋዩን የህመማቸውንና የሌሎች ችግሮቻቸው መንስኤ ምን ሊሆን እንደሚችል የሚያስቡትን ትጠይቃለች፡፡ እንዲሁም ቤተሰብ ወይም በዙሪያቸው ያሉ ድጋፍ የሚሰጡት ወዳጅ ዘመድ ተመሳሳይ ማብራሪያ ያለቸው መሆኑን ታጣራለች፡፡ |
| 8. ችግሮችንና ጫናዎችን ለመቋቋም የሚጠቀሙባቸውን ዘዴዎችና ቀደም ሲል የተጠቀሟቸውን መፍትሔዎች የመመርመር ክህሎት_ _ ተግባራዊ አይደለም | | |
| ሀ | መሻሻል ያስፈልገዋል | = ባለሙያዋ ተገልጋዩን ችግሩን እንዴት እንደተቋቋሙት አትጠይቅም ወይም ተገልጋዩን ችግሩን ስለተቋቋሙበት መንገድ ትችት/ ፍርድ አዘል ንግግር ትናገራለች፡፡ (ለምሳሌ፡ “ለምን ያ ነገር ይሆናል ብለው አሰቡ?” ወይም “ያ ነገር አይጠቅምዎትም፡፡”) |
| ለ | በከፊል የተፈጸመ | = ባለሙያዋ ተገልጋዩን ችግሩን እንዴት እንደተቋቋሙትና የቅድሚያ መፍትሄዎችን ትጠይቃለች፤ ነገር ግን አዎንታዊ አስተያየት አትሰጣቸውም፡፡ |
| ሐ | በትክክል የተፈጸመ | = ባለሙያዋ ተገልጋዩን ችግሩን እንዴት እንደተቋቋሙት ትጠይቃለች፡፡ እንዲሁም አዎንታዊ ምላሽ ትሰጣለች፡፡ |
| 9. የተገልጋዩን የቅርብ ጊዜ አስጨናቂ የሆኑ ሁኔታዎች በማህበራዊና ስነልቦናዊ ጤናቸው ላይ ያሳደረውን ተፅእኖ የመዳሰስ ክህሎት _ _ ተግባራዊ አይደለም | | |
| ሀ | መሻሻል ያስፈልገዋል | = ባለሙያዋ ስለ ወቅታዊ አስጨናቂ ሁኔታዎች (ገጠመኞች) አትጠይቃቸውም፡፡ |
| ለ | በከፊል የተፈጸመ | = ባለሙያዋ ስለ ወቅታዊ አስጨናቂ ሁኔታዎች ትጠይቃለች ግን ከወቅታዊ የአእምሮ ህመም ጋር ግንኙነቱን አታይም፡፡ |
| ሐ | በትክክል የተፈጸመ | = ባለሙያዋ ስለ ወቅታዊ አስጨናቂ ሁኔታዎች ትጠይቃለች ከአእምሮ ህመም ጋር ያለውንም ግንኙነት ትወያያለች፡፡ |
| 10. የአልኮል ወይም ዕፅ አወሳሰድ ሁኔታን (በሀኪም የሚታዘዙ መድሀኒቶችን ያለአግባብ መጠቀምን ጨምሮ) የመዳሰስ ክህሎት _ _ ተግባራዊ አይደለም | | |
| ሀ | መሻሻል ያስፈልገዋል | = ባለሙያዋ ስለ አልኮል ወይም ዕፅ አወሳሰድ ሁኔታ (በሀኪም የሚታዘዙ መድሀኒቶችን ያለአግባብ መጠቀምን ጨምሮ) አትጠይቅም፡፡ ወይም ስለ አልኮል ወይም ዕፅ አወሳሰድ ሁኔታ ተገቢ ባልሆነ መንገድ ወይም ለህመምተኛው እድሜና ፆታ በማይመጥን ወይም ስሜት አልባ በሆነ መንገድ ትጠይቃለች |
| ለ | በከፊል የተፈጸመ | = ባለሙያዋ ከፊል ታሪኩን ትወስዳለች ነገር ግን ስለ አልኮል ወይም ዕፅ አወሳሰድ ሁኔታ (በሀኪም የሚታዘዙ መድሀኒቶችን ያለአግባብ መጠቀምን ጨምሮ) ለተሰጡ አዎንታዊ ምላሾች ጥልቅ ምርመራ አታካሂድም፡፡ |
| ሐ | በትክክል የተፈጸመ | = ባለሙያዋ ስለ አልኮል ወይም ዕፅ አወሳሰድ ጉዳዮች (በሀኪም የሚታዘዙ መድሀኒቶችን ያለአግባብ መጠቀምን ጨምሮ) ዳሰሳ ታካሂዳለች እና አስፈላጊ ሲሆን ከተገልጋዩ ሁኔታ ጋር ያለውን ዝምድና ታብራራለች፡፡ ወይም ባለሙያዋ ስለ አልኮል ወይም ዕፅ አወሳሰድ ሁኔታ ለህመምተኛው እድሜና ፆታ ተገቢ በሆነ መንገድ ትጠይቃለች፡፡ ወይም ባለሞያዋ ስለ ተገልጋዩ የቅርብ ቤተሰቦች የአልኮል ወይም ዕፅ አወሳሰድ ሁኔታ ትጠይቃለች፡፡ |
| 11. ተገቢ የሆነ የቤተሰብ አባል፣ የወዳጅ ዘመድና የተንከባካቢ ተሳትፎ የመዳሰስ ክህሎት _ _ ተግባራዊ አይደለም | | |
| ሀ | መሻሻል ያስፈልገዋል | = ባለሙያዋ ከተገልጋዩ ቤተሰብ አባላት ብቻ ጋር ታወራለች፤ ለተገልጋዩ አስተያየት ተገቢውን ክብር አትሰጥም፡፡(ለምሳሌ፡ ቤተሰቦችህ የሚሉህን ሁሉ በደንብ ማዳመጥ አለብህ፡፡)፡፡ወይም (በውይይቱ ጊዜ ቤተሰብ ካልተገኘ) ህመምተኛውን ስለ ቤተሰቦቹ ተሳትፎ አትጠይቅም፡፡ |
| ለ | በከፊል የተፈጸመ | = ባለሙያዋ ስለ ቤተሰብ ተሳትፎ ትጠይቃለች ነገር ግን የተሳተፉበትን ወይም ያልተሳተፉበትን ምክንያት  ከህመምተኛው በጥልቀት አትመረምርም፡፡ |
| ሐ | በትክክል የተፈጸመ | =ባለሙያዋ የተገልጋዩን አስተያየት፤ ምን ያህል የቤተሰብ ተሳትፎ እንደሚፈልጉ፤ የሚያካትት የህክምና ዕቅድ አውጥታለች (በውይይቱ ጊዜ ቤተሰቦቹ ባይገኙም) እንዲሁም የሁለቱን ወገኖች ተግባቦት ታበረታታለች፡፡ |

| 12. የጋራ ግብ ማስቀመጥና ተገልጋዩ ከህክምናው ስለሚጠብቀው ውጤት የመዳሰስ ከህሎት_ _ ተግባራዊ አይደለም | | | |
| --- | --- | --- | --- |
| ሀ | መሻሻል ያስፈልገዋል | = ባለሙያዋ ተገልጋዩን በህክምናው ላይ ስላስቀመጧቸው ግቦችና ስለሚጠብቁት ውጤት አትጠይቃቸውም ወይም ባለሙያዋ የተገልጋዩን የወደፊት ተስፋ ሳትጠይቅ ተገልጋዩን ምን ማድረግ እንዳለባቸው ብቻ ትነግራቸውለች፡፡ | |
| ለ | በከፊል የተፈጸመ | = ባለሙያዋ ተገልጋዩን በህክምናው ላይ ያስቀመጧቸውን ግቦችና ስለሚጠብቋቸው ውጤት ትጠይቃለች ነገር ግን ግቦቹ መተግበር የሚችሉ ወይም ተጨባጭ ሁኔታ ላይ የተመሰረቱ ስለመሆናቸው አታወያያቸውም፡፡ | |
| ሐ | በትክክል የተፈጸመ | = ባለሙያዋ ተገልጋዩን በህክምናው ላይ ያስቀመጧቸውን ግቦች ትጠይቃለች እንዲሁም ተገልጋዩን የትኞቹ በህክምና መተግበር የሚችሉ ወይም ተጨባጭ ሁኔታ ላይ የተመሰረቱ እና የትኞቹ ደግሞ ተጨባጭ እንዳልሆኑ ታወያያቸዋለች፡፡ ከዚያም ህመምተኛውና ባለሙያዋ በጋራ የህክምና ዕቅድ ያዘጋጃሉ፡፡ | |
| 13. ተጨባጭነት ያለውን የለውጥ ተስፋ ስለማበረታታት _ _ ተግባራዊ አይደለም | | | |
| ሀ | መሻሻል ያስፈልገዋል | = ባለሙያዋ ምንም ተስፋ አትሰጥም (ለምሳሌ፡ “አንተ በፍጹም ሊሻልህ አይችልም፡፡” ትላለች፡፡) ወይንም ከህክምናው ስለሚገኘው ጥቅም ተጨባጭ ያልሆነ ተስፋ ትሰጣለች፡፡ (ለምሳሌ፡ “አንተ አሁን በጥቂት ቀናት ውስጥ ትድናለህ:: እና ከዚያ በኋላ ምንም አይነት ችግር ፈጽሞ በድጋሚ አይገጥምህም፡፡” ትላቸዋለች) | |
| ለ | በከፊል የተፈጸመ | = ባለሙያዋ ተገልጋዩን ስለህክምናው ሂደት ብዙም አትነግራቸውም፡፡ | |
| ሐ | በትክክል የተፈጸመ | = ባለሙያዋ ተገልጋዩን ስለወደፊቱ አወንታዊ ስሜት እንዲኖራቸው ትረዳለች እንዲሁም በህክምናው ወቅት ሊሳኩ ስለሚችሉትና ስለሚያገኘኙት ጥቅም ተጨባጭ ነገር እንዲጠብቁ ታደርጋለች፡፡ በተጨማሪም ስለህክምናው ስታብራራ ተገልጋዩ መረዳታቸውን ታረጋግጣለች፡፡ | |
| 14.የስነልቦና ትምህርት ስታቀርብ በአካባቢው አግባብነት ያላቸውን ቃላቶች የመጠቀም ካህሎት _ _ ተግባራዊ አይደለም | | | |
| ሀ | መሻሻል ያስፈልገዋል | = ባለሙያዋ ስለ አእምሮ ህመም ለማብራራት ግልጽ ያልሆኑ ቃላትን ትጠቀማለች ወይም ክብረ-ነክ ቃላትን ትጠቀማለች ወይም ህክምናው እንዴት እንደሚሰራ አታብራራም፡፡ | |
| ለ | በከፊል የተፈጸመ | = ባለሙያዋ የተወሰኑ ግልጽ ያልሆኑ ቃላትን ትጠቀማለች ነገር ግን ምንም ክብረ-ነክ ቃላት አትጠቀምም፡፡ | |
| ሐ | በትክክል የተፈጸመ | = ባለሙያዋ የስነልቦና ትምህርት ለመስጠት በአካባቢው የተለመዱ ቃላትን ትጠቀማለች፤ ስለ አእምሮ ጤናና ህክምና ስታብራራ በአካባቢው የተለመዱ አገላለጾችን ትጠቀማለች፡፡ እንዲሁም ተገልጋዩ እንደተረዳት ታረጋግጣለች፡፡ | |
| 15. ችግርን በግልጽና በትክክል መተንተን፣ ቅደም ተከተል ማስያዝ፣ መፍትሄውን ተግባር ላይ የማዋል ክህሎት  _ _ ተግባራዊ አይደለም | | | |
| ሀ | መሻሻል ያስፈልገዋል | | = ባለሙያዋ ችግርን በግልጽና በትክክል ለመተንተን ከተ.ቁ 2-4 (ከታች ተመልከት) ለማከናወን ትሞክራለች ነገር ግን 1 ወይም 2ቱን ብቻ በትክክል ታጠናቅቃለች፡፡ |
| ለ | በከፊል የተፈጸመ | | =ባለሙያዋ ችግርን በግልጽና በትክክል ለመተንተን ከተ.ቁ 2-4 (ከታች ተመልከት) ለማከናወን ትሞክራለች ነገር ግን 3ቱን ብቻ በትክክል ታጠናቅቃለች፡፡ |
| ሐ | በትክክል የተፈጸመ | | = ባለሙያዋ ተገልጋዩ የሚከተሉትን እንዲያከናውኑ ታግዛቸዋለች፡፡ (1) ዋና ችግሮቻቸው ምን ማለት እንደሆኑ እንዲሁም ቅደም ተከተል እንዲያስይዙ ትረዳለች፤ (2) መፍተሄ ሊሆኑ የሚችሉ እርምጃዎችን እንዲጠቅሱ፤ (3) ጥቅሞችንና ጉዳቶችን ይተነትናሉ፤ እና (4) እቅድ ይነድፋሉ፡፡ |

| 16. ግብረ መልስ (Feedback) መቀበል እና ምክር፣ አስተያየትና ሀሳብ መስጠት _ _ ተግባራዊ አይደለም | | | | |  |
| --- | --- | --- | --- | --- | --- |
| ሀ | | መሻሻል ያስፈልገዋል | = ባለሙያዋ በተገልጋዩ ዘንድ ተቀባይነት ይኑረው አይኑረው/ይስሟት አይስሟት ሳትጠይቅ ዲስኩር (Lecture) ትሰጣለች፡፡ | |  |
| ለ | | በከፊል የተፈጸመ | = ባለሙያዋ ለተገልጋዩ ጠቃሚ የሆኑ ምክሮችን ትሰጣለች፡፡ ነገር ግን ምክሩ የሚረዳ መሆን አለመሆኑን ከህመምተኛው ለመረዳት አትሞክርም፡፡ | |  |
| ሐ | | በትክክል የተፈጸመ | = ባለሙያዋ ለተገልጋዩ ተገቢ ምክር ትሰጣለች ምክሩ ጠቃሚ ስለመሆኑም ግብረ-መልስ በግልጽ ትጠይቃለች፡፡ | |  |
| 17. ባለሙያዋ ለተገልጋዩ ውይይታቸው በሚስጥር እንደሚያዝ ማስረዳትን በተግባር የማረጋገጥን በተመለከተ  _ _ ተግባራዊ አይደለም | | | | |  |
| ሀ | | መሻሻል ያስፈልገዋል | = ባለሙያዋ ስለተገልጋዩ መረጃ በሚስጥር መጠበቅ አታወራም፡፡ ወይም የሁኔታና የቦታውን መመቻቸት አታጣራም፡፡ ለምሳሌ ቤተሰብ ባለበት በምን ጉዳይ መነጋገር አግባብነት እንዳለው አታስብም፡፡ | |  |
| ለ | | በከፊል የተፈጸመ | = ባለሙያዋ ተገልጋዩ በእራሱ ወይም በሌሎች ላይ ጉዳት እንደሚያደርስ እያወቀችም ሚስጥሩ እንደሚጠበቅ ትነግረዋለች፡፡ | |  |
| ሐ | | በትክክል የተፈጸመ | = ባለሙያዋ ተገልጋዩ በእራሱ ወይም በሌሎች ላይ ጉዳት ሊደርስ ካልሆነ በቀር ሁሉም ንግግሮቻቸው በሚስጥር እንደሚጠበቁ ትነግራቸዋለች ወይም ለውይይታቸው ቦታና ሁኔታ ታመቻቻለች፡፡ | |  |
| 18. ራስን ወይም ሌሎችን የመጉዳት፣ በሌሎች መጎዳት መፍትሔውን በጋራ ማቀድ _ _ ተግባራዊ አይደለም | | | | |  |
| ሀ | መሻሻል ያስፈልገዋል | | | = ባለሙያዋ ተገልጋዩ በእራሳቸው ወይም በሌሎች ላይ ጉዳት ስለማድርስ አትጠይቅም ወይንም ራስን ስለመጉዳት/ ራስን ስለማጥፋት ለሚጠቁሙ ሀሳቦች ትኩረት አትሰጥም፡፡ |  |
| ለ | በከፊል የተፈጸመ | | | = ባለሙያዋ ተገልጋዩ በእራሳቸው ወይም በሌሎች ላይ ጉዳት ስለማድርስ ትጠይቃለች፡፡ ነገር ግን ተገልጋዩ ለዚህ ችግር የመፍትሔ ዕቅድ እንዲያዘጋጁ አትረዳቸውም፡፡ |  |
| ሐ | በትክክል የተፈጸመ | | | = ባለሙያዋ ተገልጋዩ በእራሳቸው ወይም በሌሎች ላይ ጉዳት ስለማድርስ ትጠይቃለች እና ደህንነታቸውን ለማረጋገጥ የሚወሰድ እርምጃን ታስተባብራለች፡፡ |  |
| \| 19. የአንጎለ- መታወክ (anti-psychotic) መድሀኒት አቅርቦትንና መድሀኒቱን በአግባቡ መውሰድን መዳሰስ _ _ ተግባራዊ አይደለም \| \| \| \| \| \| \| --- \| --- \| --- \| --- \| --- \| --- \| \| ሀ \| የፍላጎት መሻሻል \| \| \| \| = የአንጎለ- መታወክ (anti-psychotic) መድሀኒት በአግባቡ መውሰዳቸውንን አትጠይቅም ወይም መድሃኒት በአግባቡ ስላለመውሰዳቸው ትወቅሳቸዋለች፡፡ \| \| ለ \| በከፊል የሚፈጸሙ \| \| \| \| = የአንጎለ- መታወክ (anti-psychotic) መድሀኒት በአግባቡ መውሰዳቸውን ትጠይቃለች ነገር ግን መድሀቱን በአግባቡ እንዳይወስዱ ያደረጋቸውን ምክንያት አትጠይቅም፤ እና /ወይም መድሃኒት በአግባቡ ለመውሰድ የሚረዱ ዘዴዎችን አትጠይቅም፡፡ \| \| ሐ \| ሙሉ በሙሉ ይደረጋል \| \| \| \| = የአንጎለ- መታወክ (anti-psychotic) መድሀኒት በአግባቡ መውሰዳቸውን ትጠይቃለች ፡፡ እንዲሁም መድሀኒት በአግባቡ ለመውሰድ ያጋጠሟቸውን ችግሮች ካሉ ትጠይቃለች ምክንያታቸውንም ለመረዳት ትሞክራለች፡፡ በተጨማሪም መድሃኒት በአግባቡ ለመውሰድ የሚረዱ ዘዴዎችን ትጠይቃለች፡፡ \| \| ማህበረሰቡ ወይም ዘመድ ወዳጅ ለችግሮች መፍትሔ መሆኑን መዳሰስ _ _ ተግባራዊ አይደለም \| \| \| \| \| \| \| ሀ \| የፍላጎት መሻሻል \| \| = ማህበረሰቡና ዘመድ ወዳጅ በህመምተኛው የጤና መሻሻል ላይ ስላለው የመፍትሄ ሚና አስተያየት አትጠይቅም፡፡ \| \| \| \| ለ \| በከፊል የሚፈጸሙ \| \| = ማህበረሰቡና ዘመድ ወዳጅ በህመምተኛው የጤና መሻሻል ላይ ስላለው የመፍትሄ ሚና አስተያየት ትሰጠዋለች ነገር ግን የህመምተኛውን አስተያየት አትጠይቅም፡፡ \| \| \| \| ሐ \| ሙሉ በሙሉ ይደረጋል \| \| = የማህበረሰቡንና የዘመድ ወዳጅ ግንኙነትን ማጠናከር በህመምተኛው የጤና መሻሻል ላይ ስላለው የመፍትሄ ሚና አስተያየት ትሰጠዋለች የህመምተኛውንም አስተያየት ትጠይቃለች፡፡ \| \| \| \| የአካላዊ ጤና ጉዳዮችን የመዳሰስ ክህሎት _ _ ተግባራዊ አይደለም \| \| \| \| \| \| \| ሀ \| \| መሻሻል ያስፈልገዋል \| \| = ባለሙያዋ ስለ አካላዊ ጤና እና ስለ አካላዊ ጤና ጉዳዮች አትጠይቅም፡፡ \| \| \| ለ \| \| በከፊል የተፈጸመ \| \| = ባለሙያዋ ከፊል ታሪኩን ትወስዳለች ነገር ግን ስለ አካላዊ ጤና ጉዳዮች ለተሰጡ አዎንታዊ ምላሾች ጥልቅ ምርመራ አታካሂድም፡፡ \| \| \| ሐ \| \| በትክክል የተፈጸመ \| \| = ባለሙያዋ ስለተጓዳኝ አካላዊ የጤና ጉዳዮች ዳሰሳ ታካሂዳለች እና አስፈላጊ ሲሆን ከተገልጋዩ ሁኔታ ጋር ያለውን ዝምድና ታብራራለች፡፡ \| \| | | | | | |

## MINI Suicidality Scale

በስተቀኝ መጨረሻ ረድፍ ቁጥር ላላቸው ጥያቄዎች መልሱ አዎ ከሆነ ቁጥሩን አክብብ፡፡

| በባለፉት 30 ቀናት ውስጥ | | | | |
| --- | --- | --- | --- | --- |
| 9001_1 | ድንገተኛ አደጋ አጋጥሞታል?  ይህ በድንገት መድሀኒት ከመጠን በላይ መውሰድን ይጨምራል፡፡ | አዎ | 1 |  |
|  |  | የለም | 0 |  |
|  | መልሱ “የለም” ከሆነ 🡪 9004_1  መልሱ “አዎ” ከሆነ 🡪 9002_1 | | | |
| 9002_1 | ሆነ ብለው ራስዎን ለመጉዳት አቅደው ያውቃሉ? (ለምሳሌ ራስዎን ለአደጋ በማጋለጥ) | አዎ | 1 |  |
|  |  | የለም | 0 |  |
|  | መልሱ “የለም” ከሆነ 🡪 9004_1  መልሱ “አዎ” ከሆነ 🡪 9003_1 | | | |
| 9003_1 | ራስዎን ለአደጋ በማጋለጥ ህይወትዎን ለማጥፋት አስበው ያውቃሉ? | አዎ | 1 |  |
|  |  | የለም | 0 |  |
|  | | | | |
| 9004_1 | ተስፋ የመቁረጥ ስሜት ይሰማዎታል? | አዎ | 1 | 1 |
|  |  | የለም | 0 |  |
|  | | | | |
| 9005_1 | ብሞት ይሻለኛል ብለው ያስባሉ ወይም ብሞት ብለው ይመኛሉ? | አዎ | 1 | 1 |
|  |  | የለም | 0 |  |
|  | | | | |
| 9006_1 | ሊሞቱ እንደሚችሉ እያወቁ ወይም ለመሞት በማቀድ እራስዎን ለመጉዳት ወይም ለማቁሰል ያስባሉ ወይም በአእምሮዎ እራስዎን የመጉዳት ምስል ይመጣብዎታል? | አዎ | 1 | 4 |
|  |  | የለም | 0 |  |
|  | መልሱ “የለም” ከሆነ 🡪 9008_1  መልሱ “አዎ” ከሆነ 🡪 9007_1 | | | |
| 9007_1 | ምንያህል ጊዜ አስበው ያውቃሉ? | [ ] [ ] | | |
| 9008_1 | እራስዎን ለማጥፋትወይም እራስዎን ለመግደል ያስባሉ? | አዎ | 1 | 6 |
|  |  | የለም | 0 |  |
|  | መልሱ “የለም” ከሆነ 🡪 9013_1  መልሱ “አዎ” ከሆነ 🡪 9009_1 | | | |
| 9009_1 | እራስዎን የማጥፋት ሐሳብ ለምን ያህል ጊዜ ነበረዎት? | [ ] [ ] | | |
| 9010_1 | እራስዎትን የማጥፋት ሐሳብዎ ምን ያህል ይደጋገም ነበር? | አልፎ አልፎ | 1 |  |
|  |  | ሁል ጊዜ | 2 |  |
|  |  | ዘወትር | 3 |  |
|  | | | | |
| 9011_1 | እራስዎትን የማጥፋት ሐሳብዎ ምን ያህል ከባድ ነበር? | ቀላል | 1 |  |
|  |  | መካከለኛ | 2 |  |
|  |  | ከባድ | 3 |  |
|  | | | | |
| 9012_1 | ይህንን እራስዎትን የማጥፋት ስሜት ለመቆጣጠር ያቅቶታል? | አዎ | 1 | 8 |
|  |  | የለም | 0 |  |
|  | | | | |
| 9013_1 | እራስዎትን ለማጥፋት እቅድ ወይም እራስዎትን የማጥፊያ ዘዴ በልብዎ አዘጋጅተዎል (ለምሳሌ እንዴት፣ መቼ እና የት)? | አዎ | 1 | 8 |
|  |  | የለም | 0 |  |
|  | መልሱ “አዎ” ከሆነ 🡪 9014_1  መልሱ “የለም” ከሆነ 🡪 9015_1 | | | |
|  | | | | |
| 9014_1 | እራስዎን ለማጥፋትያዘጋጁትን እቅድ ለመሞከር ያስባሉ? | አዎ | 1 | 8 |
|  |  | የለም | 0 |  |
|  | | | | |
| 9015_1 | እራስዎን ለማጥፋት በሚያደርጉት ሙከራ ለመሞት ያስባሉ? | አዎ | 1 | 9 |
|  |  | የለም | 0 |  |

| 9016_1 | ለመሞት በማሰብ ወይም በመፈለግ እራስዎን ለመጉዳት ወይም ህይወትዎን የማጥፋት ሙከራ ለማድረግ ዝግጅት አድርገው ወይም ተንቀሳቅሰው ያውቃሉ? | | አዎ | 1 |  |
| --- | --- | --- | --- | --- | --- |
|  |  |  | የለም | 0 |  |
|  | **መልሱ “የለም” ከሆነ 🡪 9018_1** | |  | | |
| 9017_1 | ለምን ያህል ጊዜ ዝግጅት አድርገው ወይም ተንቀሳቅሰው ያውቃሉ? | | ___ ___ | | |
| 9018_1 | በባለፈው እንድ ወር ጊዜ ውስጥ ሕይወትዎን ለማጥፋት ሐሳብ ሳይኖረዎ እራስዎን ሆነ ብለው ጎድተዋል? | | አዎ | 1 | **4** |
|  |  |  | የለም | 0 |  |
|  | | | | | |
| 9019_1 | በባለፈው እንድ ወር ጊዜ ውስጥ እራስዎን የማጥፋት ወይም እራስዎን የመግደል ሙከራ አድርገዋል?  እራስን የማጥፋት ሙከራ ማለት ቢያንስ በጥቂቱም ቢሆን እራስዎን ለማጥፋት እያሰቡ ሊሚጎዳዎ የሚችልን ነገር ማድረግ ማለት ነው፡፡ | | አዎ | 1 | **9** |
|  |  |  | የለም | 0 |  |
|  | **መልሱ “የለም” ከሆነ 🡪 9023_1**  **መልሱ “አዎ” ከሆነ 🡪 9020_1** | | | | |
|  | | | | | |
| 9020_1 | በባለፈው እንድ ወር ጊዜ ውስጥ ለምን ያህል ጊዜ እራስዎን የማጥፋት ወይም እራስዎን የመግደል ሙከራ አድርገዋል? | | ___ ___ | | |
| 9021_1 | እራስዎን የማጥፋት ወይም እራስዎን የመግደል ሙከራ ሲያደርጉ እንደሚተርፉ ተስፋ አድርገው ነበር? | | አዎ | 1 |  |
|  |  |  | የለም | 0 |  |
|  | | | | | |
| 9022_1 | እራስዎን የማጥፋት ወይም እራስዎን የመግደል ሙከራ ሲያደርጉ እንደሚሞቱ አስበው ወይም እሞታለሁ ብለው ጠብቀው ነበር? | | አዎ | 1 |  |
|  |  |  | የለም | 0 |  |
|  | | | | | |
| 9023_1 | በሕይወት ዘመንዎ እራስዎን የማጥፋት ወይም እራስዎን የመግደል ሙከራ አድርገው ያውቃሉ? | | አዎ | 1 | **4** |
|  |  |  | የለም | 0 |  |
| 9024_1 | **ለፎርም 9 ዋና የመረጃ ምንጭ ማን ነበር** | | **ሕመምተኛወ/ዋ** | **1** |  |
|  |  |  | **አስታማሚ** | **2** |  |
|  |  |  | **ሁለቱም** | **3** |  |
| **በስተቀኝ መጨረሻ ረድፍ ላይ የተከበቡ ቁጥሮችን ደምረህ/ሽ አስቀምጥ/ጭ ፡፡** | | | | | |
| 9025_1 | አጠቃላይ ነጥብ | ___ ___ነጥብ | | | |
| 9026_1 | ተጠያው/ዋ እራሱን/ሷን የማጥፋት ችግር ደረጃ ምን ያህል ነው? | አነስተኛ (ከ 0 እስከ 8 ነጥብ) | | 1 |  |
|  |  | መካከለኛ (ከ 9 እስከ 16 ነጥብ) | | 2 |  |
|  |  | ከፍተኛ (17 እና ከዚያ በላይ ነጥብ) | | 3 |  |
| **9027_1** | **ግለሰቡ/ቧ 17 እና ከዚያ በላይ ነጥብ አምጥተዋል?**  **ወይም**  **ከሚከተሉት ጥያቄዎች ለአንዱ “አዎ” የሚል መልስ ሰጥተዋል?**  **9012_1**  **9013_1**  **9014_1**  **9015_1**  **9019_1** |  | | |  |
|  |  | አዎ | | 1 |  |
|  |  | የለም | | 0 |  |
|  |  |  | | |  |
| **መልሱ “አዎ” ከሆነ ጥናቱ ውስጥ መካተት የለባቸዉም፡፡ እንደአስፈላጊነቱ ተገቢው ምርመራ ና ሕክምና እንዲደረግለት/ላት አድርግ/ጊ::** | | | | | |

## List of Threatening Experiences questionnaire

| 8. uc¨< LÃ K=Å`c< ¾T>‹K< SØö ¾IÃ¨ƒ ›Ò×T>­‹ | | | | | |
| --- | --- | --- | --- | --- | --- |
| 801 | **vKñƒ 6 ¨^ƒ `e­ LÃ** ŸuÉ ÁK ISU' ¾›"M Ñ<Çƒ ¨ÃU ÉwÅv ›ÒØV­ƒ ’u`; | ›­ | 1 |  | LEILL9 |
|  |  | ¾KU | 2 |  |  |
|  |  | ›L¨<pU | 8 |  |  |
|  |  | SMe SeÖƒ ›MðKÑ<U | 9 |  |  |
| 802 | **vKñƒ 6 ¨^ƒ** ¨<eØ up`w ²SÉ­ LÃ ŸuÉ ÁK ISU' ÉwÅv ¨ÃU ¾›"M Ñ<Çƒ Å`f ’u`; | ›­ | 1 |  | LEIRE9 |
|  |  | ¾KU | 2 |  |  |
|  |  | ›L¨<pU / ›Le¨<eU | 8 |  |  |
|  |  | SMe SeÖƒ ›MðKÑ<U | 9 |  |  |
| 803 | **vKñƒ 6 ¨^ƒ** ¨<eØ vKu?ƒ­& Ÿ¨LÐ‹­ ›”Æ ¨ÃU ŸMÐ‹­ ›”Æ(›”ÇD) ¾V} c¨< ’u`; | ›­ | 1 |  | LEBE9 |
|  |  | ¾KU | 2 |  |  |
|  |  | ›L¨<pU | 8 |  |  |
|  |  | SMe SeÖƒ ›MðKÑ<U | 9 |  |  |
| 804 | **vKñƒ 6 ¨^ƒ** ¨<eØ ¾V}w­ ¾u?}cw p`w ÕÅ— ¾J’ c¨< ¨ÃU K?L ¾p`w ²SÉ ›K; | ›­ | 1 |  | LEBEF9 |
|  |  | ¾KU | 2 |  |  |
|  |  | ›L¨<pU | 8 |  |  |
|  |  | SMe SeÖƒ ›MðKÑ<U | 9 |  |  |
| 805 | **vKñƒ 6 ¨^ƒ** ¨<eØ uƒÇ` ¨<eØ u}ðÖ[ ›KSeTTƒ U¡”Áƒ ŸvKu?ƒ­ }KÁÃ}¨< Á¨<nK<; | ›­ | 1 |  | LEMAR9 |
|  |  | ¾KU | 2 |  |  |
|  |  | ›ÃSKŸƒU | 8 |  |  |
|  |  | SMe SeÖƒ ›MðKÑ<U | 9 |  |  |
| 806 | **vKñƒ 6 ¨^ƒ** ¨<eØ Ö”"^ ¾’u[ Ó”–<’ƒ ¨Ã”U ÕÅ˜’ƒ ›õ`cªM; | ›­ | 1 |  | LEREL9 |
|  |  | ¾KU | 2 |  |  |
|  |  | ›L¨<pU | 8 |  |  |
|  |  | SMe SeÖƒ ›MðKÑ<U | 9 |  |  |
| 807 | **vKñƒ 6 ¨^ƒ ¨<eØ** u`e­“ up`w ÕÅ™‹­' Ô[u?„‹­ ¨Ã”U ²SÊ‹­ S"ŸM Ö”Ÿ` ÁK ‹Ó` (¨ÃU Öw) ›ÒØV Á¨<nM; | ›­ | 1 |  | LEFRE9 |
|  |  | ¾KU | 2 |  |  |
|  |  | ›L¨<pU | 8 |  |  |
|  |  | SMe SeÖƒ ›MðKÑ<U | 9 |  |  |
| 808 | **vKñƒ 6 ¨^ƒ ¨<eØ** Ÿ›pU­ uLÃ ¾J’ ŸvÉ ¾Ñ”²w ‹Ó` ›ÒØV­ƒ ’u`; **(ŸuÉ ÁK Ñ”²w ¾T×ƒ ß”p)?** | ›­ | 1 |  | LEFIN9 |
|  |  | ¾KU | 2 |  |  |
|  |  | ›L¨<pU | 8 |  |  |
|  |  | SMe SeÖƒ ›MðKÑ<U | 9 |  |  |
| 809 | **vKñƒ 6 ¨^ƒ ¨<eØ** `e­ ƒMp ÓUƒ ¾T>cÖ<ƒ °n Öõ„w­ƒ ¨ÃU }c`qw­ƒ Á¨<nM; | ›­ | 1 |  | LETHF9 |
|  |  | ¾KU | 2 |  |  |
|  |  | ›L¨<pU | 8 |  |  |
|  |  | SMe SeÖƒ ›MðKÑ<U | 9 |  |  |
| 810 | vKñƒ 6 ¨^ƒ ¨<eØ ŸþK=e Ò` ¾T>ÁÑ“˜­ ¨ÃU õ`É u?ƒ ¾T>ÁeŸ?É ‹Ó` ’u[w­ƒ; | ›­ | 1 |  | LEPOL9 |
|  |  | ¾KU | 2 |  |  |
|  |  | ›L¨<pU | 8 |  |  |
|  |  | SMe SeÖƒ ›MðKÑ<U | 9 |  |  |
| 811 | **vKñƒ 6 ¨^ƒ ¨<eØ** vKu?ƒ­ e^ ð}¨< (e^ ›Ø J’¨<) ’u`; e^ Se^ƒ ›p…†¨< ¾’u[uƒ G<’@e ’u`; | ›­ | 1 |  | LEUNH9 |
|  |  | ¾KU | 2 |  |  |
|  |  | ›ÃSKŸƒU | 7 |  |  |
|  |  | ›L¨<pU | 8 |  |  |
|  |  | SMe SeÖƒ ›MðKÑ<U | 9 |  |  |
| 812 | **vKñƒ 6 ¨^ƒ** ¨<eØ ÃM ¾}ÖkSw­ƒ c¨< ’u`; ( KUdK? ¾S­ƒ' ¾ÅuÅu­ƒ' ¾Ñð}`­ƒ) | ›­ | 1 |  | VIOL9 |
|  |  | ¾KU | 2 |  |  |
|  |  | ›L¨<pU | 8 |  |  |
|  |  | SMe SeÖƒ ›MðKÑ<U | 9 |  |  |

| 813 | **vKñƒ 6 ¨^ƒ** ¨<eØ LÃ Ÿ²[²`“†¨< K?L u×U Áeq×­ƒ ¨ÃU Áudß­ƒ ’Ñ` ’u`; | ›­ | 1 |  | LEOTH9 |
| --- | --- | --- | --- | --- | --- |
|  |  | ¾KU | 2 |  |  |
|  |  | ›L¨<pU / ›Le¨<eU | 8 |  |  |
|  |  | SMe SeÖƒ ›MðKÑ<U | 9 |  |  |
| 814 | **c?ƒ¾ª LÃ Ÿ}²[²\ƒ ‹Óa‹ ¨<eØ ›”Æ” ¨Ã”U K?K—¨<” ’<aª†¨< ¾T>Á¨<p ŸJ’ ¾T>Ÿ}K¨<” ÖÃpÁ†¨<**  ›’²=I ‹Óa‹ vÒÖS­ƒ Ñ>²? eK‹Ó\ K=Á¨Á¿ƒ ¾T>‹K<ƒ c¨< ›Ó˜}¨< ’u`; | ›­ | 1 |  | TALK9 |
|  |  | ¾KU | 0 |  |  |

**4.3. uc¨< LÃ K=Å`c< ¾T>‹K< SØö ¾IÃ¨ƒ ›Ò×T>­‹ (List of Threatening Experiences)**

ከዚህ በታች በሁሉም ሰው ላይ ሊደርሱ የሚችሉ መጥፎ አጋጣሚዎች ተጠቅሰዋል፡፡ ስለዚህ ቀጥሎ የተጠቀሱት ገጠመኞች ባለፉት 6 ወራት ውስጥ አጋጥሞዎት ከሆነ **‹አዎ›** ካላጋጠሞዎት ደግሞ **‹የለም›** በማለት ይመልሱ፡፡

|  | **መጥፎ የህይወት አጋጣሚዎች** | **አዎ** | **የለም** |
| --- | --- | --- | --- |
| 1 | vKñƒ 6 ¨^ƒ ¨<eØ እ**`e­ LÃ ŸuÉ ÁK** **ISU**' ¾›"M Ñ<Çƒ ¨ÃU ÉwÅv ›ÒØV­ƒ ’u`; | [1] | [0] |
| 2 | vKñƒ 6 ¨^ƒ ¨<eØ **up`w ²SÉ­ LÃ** **ŸuÉ ÁK ISU' ÉwÅv** **¨ÃU ¾›"**M Ñ<Çƒ Å`f ’u`; | [1] | [0] |
| 3 | vKñƒ 6 ¨^ƒ ¨<eØ በቤተሰብ መካከል፤ ማለትም vKu?ƒ­& Ÿ¨LÐ‹­ ›”Æ ¨ÃU ŸMÐ‹­ ›”Æ (›”ÇD) **¾V} c¨<** ’u`;) | [1] | [0] |
| 4 | vKñƒ 6 ¨^ƒ ¨<eØ **¾V}w­** **p`w ÕÅ— ¾J’ c¨< ¨ÃU K?L ¾p`w ²SÉ** ›K; | [1] | [0] |
| 5 | vKñƒ 6 ¨^ƒ ¨<eØ **uƒÇ` ¨<eØ u}ðÖ[ ›KSeTTƒ** U¡”Áƒ ŸvKu?ƒ­ }KÁÃ}¨< Á¨<nK<; | [1] | [0] |
| 6 | vKñƒ 6 ¨^ƒ ¨<eØ **¾p`w ¨ÇÏ’ƒ ¨Ã”U ÕÅ˜’ƒ** ፈ`fw­ታM; | [1] | [0] |
| 7 | vKñƒ 6 ¨^ƒ ¨<eØ uእ`e­፤ up`w ÕÅ™‹­' Ô[u?„‹­ ¨Ã”U ²SÊ‹­ S"ŸM Ö”Ÿ` ÁK ‹Ó` (¨ÃU **Öw**) ›ÒØVት Á¨<nM; | [1] | [0] |
| 8 | vKñƒ 6 ¨^ƒ ¨<eØ Ÿ›pU­ uLÃ ¾J’ ŸvÉ **¾Ñ”²w ‹Ó`** ›ÒØV­ƒ ’u`; | [1] | [0] |
| 9 | Kñƒ 6 ¨^ƒ ¨<eØ ƒMp ÓUƒ ¾T>cÖ<ƒ °n ወይም **ንብረት Öõ„w­ƒ** ¨ÃU }c`qw­ƒ Á¨<nM; | [1] | [0] |
| 10 | vKñƒ 6 ¨^ƒ ¨<eØ ¨ንËM-’¡ ‹Ó` ¨ÃU **õ`É u?ƒ ¾T>ÁeŸ?É ‹Ó`** ’u[w­ƒ; | [1] | [0] |
| 11 | vKñƒ 6 ¨^ƒ ¨<eØ ባለቤትዎ e^ ð}¨< (e^ ›Ø J’¨<) ወይም **e^ Se^ƒ** **›p…†¨<** ¾’u[uƒ G<’@ታ ’u`; | [1] | [0] |
| 12 | vKñƒ 6 ¨^ƒ ¨<eØ ከባለቤትዎ ጋር **ተደባድበው** ያውቃሉ; | [1] | [0] |

## Household Food Insecurity Access Scale (HFIAS )

Amharic version

ኮድ ቁጥር-------------------------- መጠይቁ የተጀመረበት ሰዓት--------------------

መጠይቁ ያለቀበት ሰዓት-------------------------

**vKóƒ 30 k“ƒ ¨<eØ:**

| ØÁo | ¾KU | u×U ›Mö ›Mö | ›Mö ›Mö | G<MÑ>²? |
| --- | --- | --- | --- | --- |
| 1. ›”Ç”É c­‹ Ku?}cu? um UÓw ›Ã•`U ¾T>M eÒƒ ›L†¨<፤ እርስዎ Ku?}cu? um UÓw ›Ã•`U ¾T>M eÒƒ ’u[w­; | 1 | 2 | 3 | 4 |
| 1. ›”Ç”É c­‹ ¾S[Ö<ƒ” UÓw TÓ–ƒ Ápታ†ªM፤ u›pU እØ[ƒ U¡”Áƒ እ`e­ ¨ÃU K?L ¾u?}cብዎ ›vM ¾S[×‹G<ƒ” UÓw TÓ–ƒ ›p…‹G< ’u`; | 1 | 2 | 3 | 4 |
| 1. ›”Ç”É c­‹ Ømƒ }SddÃ UÓx‹” w‰ u¾k’< (u}ÅÒÒT>) KSSÑw ÃÑÅÇK<፤ u›pU እØ[ƒ U¡”Áƒ እርስዎ ¨ÃU ¾u?}cwዎ ›vM Ømƒ }SddÃ UÓx‹” w‰ u¾k’< (u}ÅÒÒT>) KSSÑw }ÑÇ‹G< ’u`; | 1 | 2 | 3 | 4 |
| 1. ›”Ç”É c­‹ ¾TÃðMÑ<ƒ” UÓw KSSÑw ¾T>ÑÅÆuƒ G<’@ታ አለ፤ u›pU እØ[ƒ U¡”Áƒ እርስዎ ¨ÃU ¾u?}cw­ ›vM ¾TƒðMÑ<ƒ” UÓw KSSÑw }ÑÇ‹G< ’u`; | 1 | 2 | 3 | 4 |
| 1. ›”Ç”É c­‹ um UÓw vKS•\ U¡”Áƒ SÖ’< Á’c UÓw KSSÑw ÃÑÅÅK<፤ um UÓw vKS•\ U¡”Áƒ እርስዎ ¨ÃU ¾u?}cwዎ ›vM SÖ’< Á’c UÓw KSSÑw }Ñዳችሁ ’u`; | 1 | 2 | 3 | 4 |
| 1. ›”Ç”É c­‹ um UÓw vKS•\ U¡”Áƒ uk” ¨<eØ Ømƒ Ñ>²? w‰ KSSÑw ÃÑÅÅK<፤ um UÓw vKS•\ U¡”Áƒ እርስዎ ¨ÃU ¾u?}cw­ ›vM uk” ¨<eØ Ømƒ Ñ>²? w‰ KSSÑw }Ñዳችሁ ’u`; | 1 | 2 | 3 | 4 |
| 1. u›”Ç”É c­‹ ቤት ¨<eØ U”U UÓw ¾TÃ•`uƒ ¨pƒ ›K፤ u›pU እØ[ƒ U¡”Áƒ በቤትዎ ¨<eØ U”U UÓw ÁM’u[uƒ ¨pƒ ’u`; | 1 | 2 | 3 | 4 |
| 1. ›”Ç”É c­‹ UÓw vKS•\ U¡”Áƒ UÓw dÃuK< Tታ/KK=ƒ ¨Å S˜ታ KSH@É ÃÑÅÇK<፤ UÓw vKS•\ U¡”Áƒ እርስዎ ¨ÃU ¾u?}cw­ ›vM UÓw dƒuK< Tታ/KK=ƒ ¨Å S˜ታ KSH@É }ÑÇ‹G< ’u`; | 1 | 2 | 3 | 4 |
| 1. ›”Ç”É c­‹ um UÓw vKS•\ U¡”Áƒ k’<” S<K< U”U UÓw dÃSÑu< c=¨<K< Ãe}¨LK<፤ um UÓw vKS•\ U¡”Áƒ እርስዎ ¨ÃU ¾u?}cw­ ›vM k’<” S<K< U”U UÓw dƒSÑu< ¨<L‹G< ታ¨<nL‹G<; | 1 | 2 | 3 | 4 |

## 1.14. OSLO 3-item social support scale

የሚከተሉት 3 ጥያቄዎች ደግሞ ከቤተሰብዎም ሆነ ከጎሮቤትዎ ጋር ያለዎትን ማህበራዊ ግንኙነት በተመለከተ ይሆናል፡፡ እባክዎ ለጥያቄዎቹ ከተሰጡት ምርጫዎች የእርስዎን ማህበራዊ ሁኔታ የሚመለከተውን ይምረጡ፡፡

| 412 | በህይወትዎ ዉስጥ **የቅርብ** የሆኑ፤ ችግርዎትንና ደስታዎትን የሚካፈሉ ስንት ሰዎች አሉ;  [1] ማንም [2] 1 ወይም 2 [3] ከ 3-5 [4] ከ5 በላይ | OSS1 |
| --- | --- | --- |
| 413 | ሌሎች ሰዎች እርስዎ በሚያደርጓቸው ነገሮች ላይ ምን ያህል **የሚጨነቁልዎትና** **የሚያስቡልዎት** ይመስልዎታል;  [5] በጣም ይጨነቁልኛል/ያስቡልኛል [4] በመጠኑ ይጨነቁልኛል/ያስቡልኛል  [3] እርግጠኛ መሆን አልችልም [2] ብዙም አያስቡልኝም [1] ጨርሶ አያስቡልኝም | OSS2 |
| 414 | ከጎረቤትዎ **እርዳታ** ባስፈለገዎ ጊዜ እገዛ ማግኘት ምን ያህል ቀላል ነው;  [5] በጣም ቀላል [4] ቀላል [3] ቀላል ባይሆንም ርዳታ ማግኘት እችላለሁ [2] ከባድ ነው [1] በጣም ከባድ ነው | OSS3 |

## Helping Alliance Questionnaire

Patient version

ከዚህ ቀጥሎ የምጠይቅዎ ደግም በባለፈው ቀጠርዎ ለአዕምሮ ጤና ሕክምና ሆስፒታል በመጡ ጊዜ ስላጋጠምዎት ሁኔታ ይሆናል፡፡ እባክዎ እያንዳንዱን ጥያቄ ሳነብልዎት ትክክለኛውን መልስ ይንገሩኝ፡፡

| 1901_1 | የጤና ባለሙያው ምን ያክል ችግርዎን የተረዳዎት ይመስልዎታል? | ምንም አልተረዳኝም | 1 |
| --- | --- | --- | --- |
|  |  | በትንሹ | 2 |
|  |  | በመጠኑ | 3 |
|  |  | ሙሉ በሙሉ | 4 |
|  | | | |
| 1902_1 | የጤና ባለሙያው የነቀፈዎት ወይም ጥፋተኛ ያደረገዎት ይመስልዎታል? | ምንም አላደረገኝም | 1 |
|  |  | በትንሹ | 2 |
|  |  | በመጠኑ | 3 |
|  |  | ሙሉ በሙሉ | 4 |
|  | | | |
| 1903_1 | የጤና ባለሙያው ለእርስዎ ምን ያክል ይጨነቅልኝና ያስብልኛል ብለው ያስባሉ? | ምንም አይጨነቅልኝም/አያስብልኝም | 1 |
|  |  | በትንሹ | 2 |
|  |  | በመጠኑ | 3 |
|  |  | ሙሉ በሙሉ | 4 |
|  | | | |
| 1904_1 | በአሁኑ ወቅት እየተጠቀሙት ያለው ህክምና ምን ያክል ትክክለኛ ነው ብለው ያስባሉ? | ምንም ትክክል አይደለም | 1 |
|  |  | በትንሹ | 2 |
|  |  | በመጠኑ | 3 |
|  |  | ሙሉ በሙሉ | 4 |
|  | | | |
| 1905_1 | የጤና ባለሙያዎን ካገኙና ከተነጋገሩ በኋላ ምን ይሰማዎታል? | አልተለወጠም/ እንዲያውም ብሶብኛል | 0 |
|  |  | የተሻለ ነው | 1 |

## Modified client service reciept inventoory

| 303 | vKñƒ 3 ¨^ƒ <___________> ¾Ö?“ ‹Ó` u’u[uƒ Ñ>²? ŸT>Ÿ}K<ƒ S<Á}™‹ ò} Kòƒ ›Ó˜}¨< eK MÌ(MÍ=…) Ö?”’ƒ Á’ÒÑ\ƒ ¾ƒ—¨<” ’¨<;  (Ié’< JeúM ¾Ñvuƒ Ñ>²?” ›ÃSKŸƒU)? | | | | | | | | | | |  |  |
| --- | --- | --- | --- | --- | --- | --- | --- | --- | --- | --- | --- | --- | --- |
| 303A | ¾Ö?“ ›?¡e}”i” c^}— | | | ›­” | 1 | 303B | | | | | | CCHEW9 | |
|  |  |  |  | ¾KU | 0 |  |  |  |  |  |  |  |  |
| 303A |  | A | ¾Ö?“ ‹Ó\ U” ’u`;  (eS<” Ãéñƒ)? | | | |  | | | | | CCHEWNA9 | |
|  |  | B | ¾ƒ ›¿; | | | | Ö?“ ŸL | | | 1 | | CCHEWLO9 | |
|  |  |  |  |  |  |  | u?ƒ | | | 0 | |  |  |
|  |  | C | vKñƒ 3 ¨^ƒ e”ƒ Ñ>²? ›¿; | | | | ______ Ñ>²? | | | | | CCHEWFR9 | |
|  |  | D | Å`f SMe Ñ<µ¨< e”ƒ c¯ƒ ðËw­ƒ; | | | | _________ c¯ƒ | | | | | CCHEWTR9 | |
|  |  | E | KÑ<µ­ƒ e”ƒ w` ŸðK<; | | | | __________ w` | | | | | CCHEWEX9 | |
|  |  | F | MÏ­ KI¡U“ c=H@É/eƒH@É T” ›wa H@Å; | | | | `e­ | | ›­” | | 1 | CCHEWMO9 | |
|  |  |  |  |  |  |  |  |  | ¾KU | | 0 |  |  |
|  |  |  |  |  |  |  | vKu?ƒ­ | | ›­” | | 1 | CCHEWHU9 | |
|  |  |  |  |  |  |  |  |  | ¾KU | | 0 |  |  |
|  |  |  |  |  |  |  | MÏ­ | | ›­” | | 1 | CCHEWLI9 | |
|  |  |  |  |  |  |  |  |  | ¾KU | | 0 |  |  |
|  |  |  |  |  |  |  | K?L ¾u?}cw ›vM | | ›­” | | 1 | CCHEWFA9 | |
|  |  |  |  |  |  |  |  |  | ¾KU | | 0 |  |  |
|  |  |  |  |  |  |  | Ô[u?ƒ | | ›­” | | 1 | CCHEWNE9 | |
|  |  |  |  |  |  |  |  |  | ¾KU | | 0 |  |  |
|  |  |  |  |  |  |  | Ô[u?ƒ ÁMJ’ K?L ÕÅ— | | ›­” | | 1 | CCHEWFR9 | |
|  |  |  |  |  |  |  |  |  | ¾KU | | 0 |  |  |
|  |  |  |  |  |  |  | ŸLÃ ÁM}Ökc K?L c¨< | | ›­” | | 1 | CCHEWOT9 | |
|  |  |  |  |  |  |  |  |  | ¾KU | | 0 |  |  |
|  |  | G | u²=I Ñ<µ U¡”Áƒ Ÿ}KSÅ¨< SÅu— e^ ¾k\ƒ e”ƒ k” ’u`? | | | | ________ k“ƒ | | | | | CCHEWOR9 | |
|  |  | H | uÑ<µ¨< U¡”Áƒ& Ÿ`e­ K?L Ÿ}KSÅ¨< SÅu— e^ ¾k\ e”ƒ c­‹ ’u\? | | | | [ ] [ ] አዋቂዎች | | | | | CCHEWNU9 | |
|  |  |  |  |  |  |  | አዋቂ 1 | [ ] [ ] k“ƒ | | | | CCHEWA19  CCHEWA29  CCHEWA39 | |
|  |  |  |  |  |  |  | አዋቂ 2 | [ ] [ ] k“ƒ | | | |  |  |
|  |  |  |  |  |  |  | አዋቂ 3 | [ ] [ ] k“ƒ | | | |  |  |
|  |  | J | uÖpLL¨< Ö?“ Ÿ?L ¨<eØ U” ÁIM Ñ>²? ›Öñ;  vKS<Áª u?ƒ­ ¨<eØ ŸÔu˜‹­ƒ& u?ƒ­ ¨<eØ U” ÁIM Ñ>²? q¾‹; | | | | ___________ c¯ƒ | | | | | CCHEWDU | |
|  |  | K | uÖpLL¨< KSÉH’>ƒ KU`S^“ KvKS<Á U” ÁIM w` ›Öñ; | | | | __________ w` | | | | | CCHEWME | |
| 303B | ¾Ö?“ ×u=Á c^}— | | | ›­” | 1 | 303C | | | | | | CCHC9 | |
|  |  |  |  | ¾KU | 0 |  |  |  |  |  |  |  |  |
|  |  | A | ¾Ö?“ ‹Ó\ U” ’u`;  (eS<” Ãéñƒ)? | | | |  | | | | | CCHCNA9 | |
|  |  | B | vKñƒ 3 ¨^ƒ e”ƒ Ñ>²? ¿; | | | | ______ Ñ>²? | | | | | CCHCFR9 | |
|  |  | C | Å`f SMe Ñ<µ¨< e”ƒ c¯ƒ ðËw­ƒ; | | | | _________ c¯ƒ | | | | | CCHCTR9 | |
|  |  | D | KÑ<µ­ƒ e”ƒ w` ŸðK<; | | | | __________ w` | | | | | CCHCEX9 | |
|  |  | E | Ö?“ ×u=Á uH@Æuƒ ¨pƒ KUÓw “ KS˜ Á¨Ö<ƒ ¨Ü U” Á¡M ’u`; | | | | __________ w` | | | | | CCHCAC9 | |
|  |  | F | MÏ­ KI¡U“ c=H@É/eƒH@É T” ›wa H@Å; | | | | `e­ | | ›­” | | 1 | CCHCMO9 | |
|  |  |  |  |  |  |  |  |  | ¾KU | | 0 |  |  |
|  |  |  |  |  |  |  | vKu?ƒ­ | | ›­” | | 1 | CCHCHU9 | |
|  |  |  |  |  |  |  |  |  | ¾KU | | 0 |  |  |
|  |  |  |  |  |  |  | MÏ­ | | ›­” | | 1 | CCHCLI9 | |
|  |  |  |  |  |  |  |  |  | ¾KU | | 0 |  |  |
|  |  |  |  |  |  |  | K?L ¾u?}cw ›vM | | ›­” | | 1 | CCHCFA9 | |
|  |  |  |  |  |  |  |  |  | ¾KU | | 0 |  |  |
|  |  |  |  |  |  |  | Ô[u?ƒ | | ›­” | | 1 | CCHCNE9 | |
|  |  |  |  |  |  |  |  |  | ¾KU | | 0 |  |  |
|  |  |  |  |  |  |  | Ô[u?ƒ ÁMJ’ K?L ÕÅ— | | ›­” | | 1 | CCHCFR9 | |
|  |  |  |  |  |  |  |  |  | ¾KU | | 0 |  |  |
|  |  |  |  |  |  |  | ŸLÃ ÁM}Ökc K?L c¨< | | ›­” | | 1 | CCHCOT9 | |
|  |  |  |  |  |  |  |  |  | ¾KU | | 0 |  |  |
|  |  | G | u²=I Ñ<µ U¡”Áƒ Ÿ}KSÅ¨< SÅu— e^ ¾k\ƒ e”ƒ k” ’u`? | | | | __________ k“ƒ | | | | | CCHCOR9 | |
|  |  | H | uÑ<µ¨< U¡”Áƒ& Ÿ`e­ K?L Ÿ}KSÅ¨< SÅu— e^ ¾k\ e”ƒ c­‹ ’u\? | | | | [ ] [ ] ›ªm­‹ | | | | | CCHCNU9 | |
|  |  |  |  |  |  |  | ›ªm 1 | [ ] [ ] k“ƒ | | | | CCHCA19  CCHCA29  CCHCA39 | |
|  |  |  |  |  |  |  | ›ªm 2 | [ ] [ ] k“ƒ | | | |  |  |
|  |  |  |  |  |  |  | ›ªm 3 | [ ] [ ] k“ƒ | | | |  |  |
|  |  | J | uÖpLL¨< Ö?“ ×u=Á ¨<eØ U” ÁIM Ñ>²? ›Öñ; | | | | ____________ c¯ƒ | | | | | CCHCDU9 | |
|  |  | K | uÖpLL¨< KSÉH’>ƒ KU`S^“ KvKS<Á U” ÁIM w` ›Öñ; | | | | __________ w` | | | | | CCHCME9 | |
| 303C | ¾u<Í=^ JeúM c^}— | | | ›­” | 1 | --$ 303D | | | | | | CCHOS9 | |
|  |  |  |  | ¾KU | 0 |  |  |  |  |  |  |  |  |
|  |  | A | ¾Ö?“ ‹Ó\ U” ’u`;  (eS<” Ãéñƒ)? | | | |  | | | | | CCHOSNA9 | |
|  |  | B | vKñƒ 3 ¨^ƒ e”ƒ Ñ>²? ¿; | | | | ______ Ñ>²? | | | | | CCHOSFR9 | |
|  |  | C | Å`f SMe Ñ<µ¨< e”ƒ c¯ƒ ðËw­ƒ; | | | | _________ c¯ƒ | | | | | CCHOSTR9 | |
|  |  | D | KÑ<µ­ƒ e”ƒ w` ŸðK<; | | | | __________ w` | | | | | CCHOSEX9 | |
|  |  | E | JeúM uH@Æuƒ ¨pƒ KUÓw “ KS˜ Á¨Ö<ƒ ¨Ü U” Á¡M ’u`; | | | | __________ w` | | | | | CCHOSAC9 | |
|  |  | F | MÏ­ KI¡U“ c=H@É/eƒH@É T” ›wa H@Å; | | | | `e­ | | ›­” | | 1 | CCHOSMO9 | |
|  |  |  |  |  |  |  |  |  | ¾KU | | 0 |  |  |
|  |  |  |  |  |  |  | vKu?ƒ­ | | ›­” | | 1 | CCHOSHU9 | |
|  |  |  |  |  |  |  |  |  | ¾KU | | 0 |  |  |
|  |  |  |  |  |  |  | MÏ­ | | ›­” | | 1 | CCHOSLI9 | |
|  |  |  |  |  |  |  |  |  | ¾KU | | 0 |  |  |
|  |  |  |  |  |  |  | K?L ¾u?}cw ›vM | | ›­” | | 1 | CCHOSFA9 | |
|  |  |  |  |  |  |  |  |  | ¾KU | | 0 |  |  |
|  |  |  |  |  |  |  | Ô[u?ƒ | | ›­” | | 1 | CCHOSNE9 | |
|  |  |  |  |  |  |  |  |  | ¾KU | | 0 |  |  |
|  |  |  |  |  |  |  | Ô[u?ƒ ÁMJ’ K?L ÕÅ— | | ›­” | | 1 | CCHOSFR9 | |
|  |  |  |  |  |  |  |  |  | ¾KU | | 0 |  |  |
|  |  |  |  |  |  |  | ŸLÃ ÁM}Ökc K?L c¨< | | ›­” | | 1 | CCHOSOT9 | |
|  |  |  |  |  |  |  |  |  | ¾KU | | 0 |  |  |
|  |  | G | u²=I Ñ<µ U¡”Áƒ Ÿ}KSÅ¨< SÅu— e^ ¾k\ƒ e”ƒ k” ’u`? | | | | __________ k“ƒ | | | | | CCHOSOR9 | |
|  |  | H | uÑ<µ¨< U¡”Áƒ& Ÿ`e­ K?L Ÿ}KSÅ¨< SÅu— e^ ¾k\ e”ƒ c­‹ ’u\? | | | | [ ] [ ] ›ªm­‹ | | | | | CCHOSNU9 | |
|  |  |  |  |  |  |  | ›ªm 1 | [ ] [ ] k“ƒ | | | | CCHOSA19  CCHOSA29  CCHOSA39 | |
|  |  |  |  |  |  |  | ›ªm 2 | [ ] [ ] k“ƒ | | | |  |  |
|  |  |  |  |  |  |  | ›ªm 3 | [ ] [ ] k“ƒ | | | |  |  |
|  |  | J | uÖpLL¨< JeúM ¨<eØ U” ÁIM Ñ>²? ›Öñ; | | | | ___________ c¯ƒ | | | | | CCHOSDU9 | |
|  |  | K | uÖpLL¨< KSÉH’>ƒ KU`S^“ KvKS<Á U” ÁIM w` ›Öñ; | | | | __________w` | | | | | CCHOSME9 | |
| 303D | ¾S`c= ýaË¡ƒ JeúM c^}— | | | ›­” | 1 | --$ 303E | | | | | | CCMP9 | |
|  |  |  |  | ¾KU | 0 |  |  |  |  |  |  |  |  |
|  |  | A | ¾Ö?“ ‹Ó\ U” ’u`;  (eS<” Ãéñƒ)? | | | |  | | | | | CCMPNA9 | |
|  |  | B | vKñƒ 3 ¨^ƒ e”ƒ Ñ>²? ¿; | | | | ______ Ñ>²? | | | | | CCMPFR9 | |
|  |  | C | Å`f SMe Ñ<µ¨< e”ƒ c¯ƒ ðËw­ƒ; | | | | _________ c¯ƒ | | | | | CCMPTR9 | |
|  |  | D | KÑ<µ­ƒ e”ƒ w` ŸðK<; | | | | __________ w` | | | | | CCMPEX9 | |
|  |  | E | JeúM uH@Æuƒ ¨pƒ KUÓw “ KS˜ Á¨Ö<ƒ ¨Ü U” Á¡M ’u`; | | | | __________ w` | | | | | CCMPAC9 | |

| 303D |  | F | MÏ­ KI¡U“ c=H@É/eƒH@É T” ›wa H@Å; | | | | `e­ | | ›­” | 1 | CCMPMO9 |
| --- | --- | --- | --- | --- | --- | --- | --- | --- | --- | --- | --- |
|  |  |  |  |  |  |  |  |  | ¾KU | 0 |  |
|  |  |  |  |  |  |  | vKu?ƒ­ | | ›­” | 1 | CCMPHU9 |
|  |  |  |  |  |  |  |  |  | ¾KU | 0 |  |
|  |  |  |  |  |  |  | MÏ­ | | ›­” | 1 | CCMPLI9 |
|  |  |  |  |  |  |  |  |  | ¾KU | 0 |  |
|  |  |  |  |  |  |  | K?L ¾u?}cw ›vM | | ›­” | 1 | CCMPFA9 |
|  |  |  |  |  |  |  |  |  | ¾KU | 0 |  |
|  |  |  |  |  |  |  | Ô[u?ƒ | | ›­” | 1 | CCMPNE9 |
|  |  |  |  |  |  |  |  |  | ¾KU | 0 |  |
|  |  |  |  |  |  |  | Ô[u?ƒ ÁMJ’ K?L ÕÅ— | | ›­” | 1 | CCMPFR9 |
|  |  |  |  |  |  |  |  |  | ¾KU | 0 |  |
|  |  |  |  |  |  |  | ŸLÃ ÁM}Ökc K?L c¨< | | ›­” | 1 | CCMPOT9 |
|  |  |  |  |  |  |  |  |  | ¾KU | 0 |  |
|  |  | G | u²=I Ñ<µ U¡”Áƒ Ÿ}KSÅ¨< SÅu— e^ ¾k\ƒ e”ƒ k” ’u`? | | | | _________ k“ƒ | | | | CCMPOR9 |
|  |  | H | uÑ<µ¨< U¡”Áƒ& Ÿ`e­ K?L Ÿ}KSÅ¨< SÅu— e^ ¾k\ e”ƒ c­‹ ’u\? | | | | [ ] [ ] ›ªm­‹ | | | | CCMPNU9 |
|  |  |  |  |  |  |  | ›ªm 1 | [ ] [ ] k“ƒ | | | CCMPA19  CCMPA29  CCMPA39 |
|  |  |  |  |  |  |  | ›ªm 2 | [ ] [ ] k“ƒ | | |  |
|  |  |  |  |  |  |  | ›ªm 3 | [ ] [ ] k“ƒ | | |  |
|  |  | J | uÖpLL¨< JeúM ¨<eØ U” ÁIM Ñ>²? ›Öñ; | | | | ____________ c¯ƒ | | | | CCMPDU9 |
|  |  | K | uÖpLL¨< KSÉH’>ƒ KU`S^“ KvKS<Á U” ÁIM w` ›Öñ; | | | | ____________ w` | | | | CCMPME9 |
| 303E | ¾ÓM ¡K=’>¡ c^}— | | | ›­” | 1 | --$ 303F | | | | | CCPC9 |
|  |  |  |  | ¾KU | 0 |  |  |  |  |  |  |
|  |  | A | ¾Ö?“ ‹Ó\ U” ’u`;  (eS<” Ãéñƒ) | | | |  | | | | CCPCNA9 |
|  |  | B | vKñƒ 3 ¨^ƒ e”ƒ Ñ>²? ¿; | | | | ______ Ñ>²? | | | | CCPCFR9 |
|  |  | C | Å`f SMe Ñ<µ¨< e”ƒ c¯ƒ ðËw­ƒ; | | | | _________ c¯ƒ | | | | CCPCTR9 |
|  |  | D | KÑ<µ­ƒ e”ƒ w` ŸðK<; | | | | __________ w` | | | | CCPCEX9 |
|  |  | E | ¡K=’>¡ uH@Æuƒ ¨pƒ KUÓw “ KS˜ Á¨Ö<ƒ ¨Ü U” Á¡M ’u`; | | | | __________ w` | | | | CCPCAC9 |
|  |  | F | MÏ­ KI¡U“ c=H@É/eƒH@É T” ›wa H@Å; | | | | `e­ | | ›­” | 1 | CCPCMO9 |
|  |  |  |  |  |  |  |  |  | ¾KU | 0 |  |
|  |  |  |  |  |  |  | vKu?ƒ­ | | ›­” | 1 | CCPCHU9 |
|  |  |  |  |  |  |  |  |  | ¾KU | 0 |  |
|  |  |  |  |  |  |  | MÏ­ | | ›­” | 1 | CCPCLI9 |
|  |  |  |  |  |  |  |  |  | ¾KU | 0 |  |
|  |  |  |  |  |  |  | K?L ¾u?}cw ›vM | | ›­” | 1 | CCPCFA9 |
|  |  |  |  |  |  |  |  |  | ¾KU | 0 |  |
|  |  |  |  |  |  |  | Ô[u?ƒ | | ›­” | 1 | CCPCNE9 |
|  |  |  |  |  |  |  |  |  | ¾KU | 0 |  |
|  |  |  |  |  |  |  | Ô[u?ƒ ÁMJ’ K?L ÕÅ— | | ›­” | 1 | CCPCFR9 |
|  |  |  |  |  |  |  |  |  | ¾KU | 0 |  |
|  |  |  |  |  |  |  | ŸLÃ ÁM}Ökc K?L c¨< | | ›­” | 1 | CCPCOT9 |
|  |  |  |  |  |  |  |  |  | ¾KU | 0 |  |
|  |  | G | u²=I Ñ<µ U¡”Áƒ Ÿ}KSÅ¨< SÅu— e^ ¾k\ƒ e”ƒ k” ’u`? | | | | ________ k“ƒ | | | | CCPCOR9 |

| 303E |  | H | uÑ<µ¨< U¡”Áƒ& Ÿ`e­ K?L Ÿ}KSÅ¨< SÅu— e^ ¾k\ e”ƒ c­‹ ’u\? | | | | [ ] [ ] ›ªm­‹ | | | | CCPCNU9 |
| --- | --- | --- | --- | --- | --- | --- | --- | --- | --- | --- | --- |
|  |  |  |  |  |  |  | ›ªm 1 | [ ] [ ] k“ƒ | | | CCPCA19  CCPCA29  CCPCA39 |
|  |  |  |  |  |  |  | ›ªm 2 | [ ] [ ] k“ƒ | | |  |
|  |  |  |  |  |  |  | ›ªm 3 | [ ] [ ] k“ƒ | | |  |
|  |  | J | uÖpLL¨< ¾ÓM ¡K=’>Ÿ< ¨<eØ U” ÁIM Ñ>²? ›Öñ; | | | | ___________ c¯ƒ | | | | CCPCDU9 |
|  |  | K | uÖpLL¨< KSÉH’>ƒ KU`S^“ KvKS<Á U” ÁIM w` ›Öñ; | | | | ___________ w` | | | | CCPCME9 |
| 303F | ¾ÓM ó`Tc=/SÉG’>ƒ u?ƒ | | | ›­” | 1 | --$ 303G | | | | | CCPHA9 |
|  |  |  |  | ¾KU | 0 |  |  |  |  |  |  |
|  |  | A | ¾Ö?“ ‹Ó\ U” ’u`;  (eS<” Ãéñƒ) | | | |  | | | | CCPHANA9 |
|  |  | B | vKñƒ 3 ¨^ƒ e”ƒ Ñ>²? SÉH’>ƒ Ñ²<; | | | | ______ Ñ>²? | | | | CCPHAFR9 |
|  |  | C | Å`f SMe Ñ<µ¨< e”ƒ c¯ƒ ðËw­ƒ; | | | | _________ c¯ƒ | | | | CCPHATR9 |
|  |  | D | KÑ<µ­ƒ e”ƒ w` ŸðK<; | | | | __________ w` | | | | CCPHAEX9 |
|  |  | E | ó`Tc= uH@Æuƒ ¨pƒ KUÓw “ KS˜ Á¨Ö<ƒ ¨Ü U” Á¡M ’u`; | | | | __________ w` | | | | CCPHAAC9 |
|  |  | F | MÏ­ KI¡U“ c=H@É/eƒH@É T” ›wa H@Å; | | | | `e­ | | ›­” | 1 | CCPHAMO9 |
|  |  |  |  |  |  |  |  |  | ¾KU | 0 |  |
|  |  |  |  |  |  |  | vKu?ƒ­ | | ›­” | 1 | CCPHAHU9 |
|  |  |  |  |  |  |  |  |  | ¾KU | 0 |  |
|  |  |  |  |  |  |  | MÏ­ | | ›­” | 1 | CCPHALI9 |
|  |  |  |  |  |  |  |  |  | ¾KU | 0 |  |
|  |  |  |  |  |  |  | K?L ¾u?}cw ›vM | | ›­” | 1 | CCPHAFA9 |
|  |  |  |  |  |  |  |  |  | ¾KU | 0 |  |
|  |  |  |  |  |  |  | Ô[u?ƒ | | ›­” | 1 | CCPHANE9 |
|  |  |  |  |  |  |  |  |  | ¾KU | 0 |  |
|  |  |  |  |  |  |  | Ô[u?ƒ ÁMJ’ K?L ÕÅ— | | ›­” | 1 | CCPHAFR9 |
|  |  |  |  |  |  |  |  |  | ¾KU | 0 |  |
|  |  |  |  |  |  |  | ŸLÃ ÁM}Ökc K?L c¨< | | ›­” | 1 | CCPHAOT9 |
|  |  |  |  |  |  |  |  |  | ¾KU | 0 |  |
|  |  | G | u²=I Ñ<µ U¡”Áƒ Ÿ}KSÅ¨< SÅu— e^ ¾k\ƒ e”ƒ k” ’u`? | | | | __________ k“ƒ | | | | CCPHAOR9 |
|  |  | H | uÑ<µ¨< U¡”Áƒ& Ÿ`e­ K?L Ÿ}KSÅ¨< SÅu— e^ ¾k\ e”ƒ c­‹ ’u\? | | | | [ ] [ ] ›ªm­‹ | | | | CCPHNU9 |
|  |  |  |  |  |  |  | ›ªm 1 | [ ] [ ] k“ƒ | | | CCPHAA19  CCPHAA29  CCPHAA39 |
|  |  |  |  |  |  |  | ›ªm 2 | [ ] [ ] k“ƒ | | |  |
|  |  |  |  |  |  |  | ›ªm 3 | [ ] [ ] k“ƒ | | |  |
|  |  | J | uÖpLL¨< ¾ÓM ó`Tc= ¨<eØ U” ÁIM Ñ>²? ›Öñ; | | | | ___________c¯ƒ | | | | CCPHADU9 |
|  |  | K | vÖpLL¨< KSÉH’>ƒ KU`S^“ KvKS<Á U” ÁIM w` ›Öñ; | | | | __________ w` | | | | CCPHAME9 |

| 303G | ¾HÃT•ƒ ›vƒ | | | ›­” | 1 | --$ 303H | | | | | CCREL9 |
| --- | --- | --- | --- | --- | --- | --- | --- | --- | --- | --- | --- |
|  |  |  |  | ¾KU | 0 |  |  |  |  |  |  |
|  |  | A | ¾Ö?“ ‹Ó\ U” ’u`;  (eS<” Ãéñƒ)? | | | |  | | | | CCRELNA9 |
|  |  | B | vKñƒ 3 ¨^ƒ e”ƒ Ñ>²? ¿; | | | | ______ Ñ>²? | | | | CCRELFR9 |
|  |  | C | Å`f SMe Ñ<µ¨< e”ƒ c¯ƒ ðËw­ƒ; | | | | _________ c¯ƒ | | | | CCRELTR9 |
|  |  | D | KÑ<µ­ƒ e”ƒ w` ŸðK<; | | | | __________ w` | | | | CCRELEX9 |
|  |  | E | ¾HÃT•ƒ ›vƒ KT“Ñ`  uH@Æuƒ ¨pƒ KUÓw “ KS˜ Á¨Ö<ƒ ¨Ü U” Á¡M ’u`; | | | | __________ w` | | | | CCRELAC9 |
|  |  | F | MÏ­ KI¡U“ c=H@É/eƒH@É T” ›wa H@Å; | | | | `e­ | | ›­” | 1 | CCRELMO9 |
|  |  |  |  |  |  |  |  |  | ¾KU | 0 |  |
|  |  |  |  |  |  |  | vKu?ƒ­ | | ›­” | 1 | CCRELHU9 |
|  |  |  |  |  |  |  |  |  | ¾KU | 0 |  |
|  |  |  |  |  |  |  | MÏ­ | | ›­” | 1 | CCRELLI9 |
|  |  |  |  |  |  |  |  |  | ¾KU | 0 |  |
|  |  |  |  |  |  |  | K?L ¾u?}cw ›vM | | ›­” | 1 | CCRELFA9 |
|  |  |  |  |  |  |  |  |  | ¾KU | 0 |  |
|  |  |  |  |  |  |  | Ô[u?ƒ | | ›­” | 1 | CCRELNE9 |
|  |  |  |  |  |  |  |  |  | ¾KU | 0 |  |
|  |  |  |  |  |  |  | Ô[u?ƒ ÁMJ’ K?L ÕÅ— | | ›­” | 1 | CCRELFR9 |
|  |  |  |  |  |  |  |  |  | ¾KU | 0 |  |
|  |  |  |  |  |  |  | ŸLÃ ÁM}Ökc K?L c¨< | | ›­” | 1 | CCRELOT9 |
|  |  |  |  |  |  |  |  |  | ¾KU | 0 |  |
|  |  | G | u²=I Ñ<µ U¡”Áƒ Ÿ}KSÅ¨< SÅu— e^ ¾k\ƒ e”ƒ k” ’u`? | | | | __________ k“ƒ | | | | CCRELOR9 |
|  |  | H | uÑ<µ¨< U¡”Áƒ& Ÿ`e­ K?L Ÿ}KSÅ¨< SÅu— e^ ¾k\ e”ƒ c­‹ ’u\? | | | | [ ] [ ] ›ªm­‹ | | | | CCRELNU9 |
|  |  |  |  |  |  |  | ›ªm 1 | [ ] [ ] k“ƒ | | | CCRELA19  CCRELA29  CCRELA39 |
|  |  |  |  |  |  |  | ›ªm 2 | [ ] [ ] k“ƒ | | |  |
|  |  |  |  |  |  |  | ›ªm 3 | [ ] [ ] k“ƒ | | |  |
|  |  | J | ¾HÃT•ƒ ›vƒ uT“ÒÑ` U” ÁIM Ñ>²? ›Öñ; | | | | __________ c¯ƒ | | | | CCRELDU9 |
|  |  | K | uÖpLL¨< KSÉH’>ƒ KU`S^“ KvKS<Á U” ÁIM w` ›Öñ; | | | | ____________ w` | | | | CCRELME9 |
| 303H | vKSÅH’>ƒ | | | ›­” | 1 | --$ 303J | | | | | CCHER9 |
|  |  |  |  | ¾KU | 0 |  |  |  |  |  |  |
|  |  | A | ¾Ö?“ ‹Ó\ U” ’u`;  (eS<” Ãéñƒ)? | | | |  | | | | CCHERNA9 |
|  |  | B | vKñƒ 3 ¨^ƒ e”ƒ Ñ>²? ¿; | | | | ______ Ñ>²? | | | | CCHERFR9 |
|  |  | C | Å`f SMe Ñ<µ¨< e”ƒ c¯ƒ ðËw­ƒ; | | | | _________ c¯ƒ | | | | CCHERTR9 |
|  |  | D | KÑ<µ­ƒ e”ƒ w` ŸðK<; | | | | __________ w` | | | | CCHEREX9 |
|  |  | E | ¨Å vKSÅH’>ƒ uH@Æuƒ ¨pƒ KUÓw “ KS˜ Á¨Ö<ƒ ¨Ü U” Á¡M ’u`; | | | | __________ w` | | | | CCHERAC9 |

| 303H |  | F | MÏ­ KI¡U“ c=H@É/eƒH@É T” ›wa H@Å; | | | | `e­ | | ›­” | 1 | CCHERMO9 |
| --- | --- | --- | --- | --- | --- | --- | --- | --- | --- | --- | --- |
|  |  |  |  |  |  |  |  |  | ¾KU | 0 |  |
|  |  |  |  |  |  |  | vKu?ƒ­ | | ›­” | 1 | CCHERHU9 |
|  |  |  |  |  |  |  |  |  | ¾KU | 0 |  |
|  |  |  |  |  |  |  | MÏ­ | | ›­” | 1 | CCHERLI9 |
|  |  |  |  |  |  |  |  |  | ¾KU | 0 |  |
|  |  |  |  |  |  |  | K?L ¾u?}cw ›vM | | ›­” | 1 | CCHERFA9 |
|  |  |  |  |  |  |  |  |  | ¾KU | 0 |  |
|  |  |  |  |  |  |  | Ô[u?ƒ | | ›­” | 1 | CCHERNE9 |
|  |  |  |  |  |  |  |  |  | ¾KU | 0 |  |
|  |  |  |  |  |  |  | Ô[u?ƒ ÁMJ’ K?L ÕÅ— | | ›­” | 1 | CCHERFR9 |
|  |  |  |  |  |  |  |  |  | ¾KU | 0 |  |
|  |  |  |  |  |  |  | ŸLÃ ÁM}Ökc K?L c¨< | | ›­” | 1 | CCHEROT9 |
|  |  |  |  |  |  |  |  |  | ¾KU | 0 |  |
|  |  | G | u²=I Ñ<µ U¡”Áƒ Ÿ}KSÅ¨< SÅu— e^ ¾k\ƒ e”ƒ k” ’u`? | | | | __________k“ƒ | | | | CCHEROR9 |
|  |  | H | uÑ<µ¨< U¡”Áƒ& Ÿ`e­ K?L Ÿ}KSÅ¨< SÅu— e^ ¾k\ e”ƒ c­‹ ’u\? | | | | [ ] [ ] ›ªm­‹ | | | | CCHERNU9 |
|  |  |  |  |  |  |  | ›ªm 1 | [ ] [ ] k“ƒ | | | CCHERA19  CCHERA29  CCHERA39 |
|  |  |  |  |  |  |  | ›ªm 2 | [ ] [ ] k“ƒ | | |  |
|  |  |  |  |  |  |  | ›ªm 3 | [ ] [ ] k“ƒ | | |  |
|  |  | J | uÖpLL¨< ŸvKSÅH’>ƒ `Ç uTÓ–ƒ U” ÁIM Ñ>²? ›Öñ; | | | | ____________ c¯ƒ | | | | CCHERDU9 |
|  |  | K | uÖpLL¨< KSÉH’>ƒ KU`S^“ KvKS<Á U” ÁIM w` ›Öñ; | | | | ___________ w` | | | | CCHERME9 |
| 303J | ¨Ñ@h | | | ›­” | 1 | 🡪303K | | | | | CCWO9 |
|  |  |  |  | ¾KU | 0 |  |  |  |  |  |  |
|  |  | A | ¾Ö?“ ‹Ó\ U” ’u`;  (eS<” Ãéñƒ)? | | | |  | | | | CCWONA9 |
|  |  | B | vKñƒ 3 ¨^ƒ e”ƒ Ñ>²? ¿; | | | | ______ Ñ>²? | | | | CCWOFR9 |
|  |  | C | Å`f SMe Ñ<µ¨< e”ƒ c¯ƒ ðËw­ƒ; | | | | _________ c¯ƒ | | | | CCWOTR9 |
|  |  | D | KÑ<µ­ƒ e”ƒ w` ŸðK<; | | | | __________ w` | | | | CCWOEX9 |
|  |  | E | ¨Ñ@h KT’ÒÑ` uH@Æuƒ ¨pƒ KUÓw “ KS˜ Á¨Ö<ƒ ¨Ü U” Á¡M ’u`; | | | | __________ w` | | | | CCWOAC9 |
|  |  | F | MÏ­ KI¡U“ c=H@É/eƒH@É T” ›wa H@Å; | | | | `e­ | | ›­” | 1 | CCWOMO9 |
|  |  |  |  |  |  |  |  |  | ¾KU | 0 |  |
|  |  |  |  |  |  |  | vKu?ƒ­ | | ›­” | 1 | CCWOHU9 |
|  |  |  |  |  |  |  |  |  | ¾KU | 0 |  |
|  |  |  |  |  |  |  | MÏ­ | | ›­” | 1 | CCWOLI9 |
|  |  |  |  |  |  |  |  |  | ¾KU | 0 |  |
|  |  |  |  |  |  |  | K?L ¾u?}cw ›vM | | ›­” | 1 | CCWOFA9 |
|  |  |  |  |  |  |  |  |  | ¾KU | 0 |  |
|  |  |  |  |  |  |  | Ô[u?ƒ | | ›­” | 1 | CCWONE9 |
|  |  |  |  |  |  |  |  |  | ¾KU | 0 |  |
|  |  |  |  |  |  |  | Ô[u?ƒ ÁMJ’ K?L ÕÅ— | | ›­” | 1 | CCWOFR9 |
|  |  |  |  |  |  |  |  |  | ¾KU | 0 |  |
|  |  |  |  |  |  |  | ŸLÃ ÁM}Ökc K?L c¨< | | ›­” | 1 | CCWOOT9 |
|  |  |  |  |  |  |  |  |  | ¾KU | 0 |  |
|  |  | G | u²=I Ñ<µ U¡”Áƒ Ÿ}KSÅ¨< SÅu— e^ ¾k\ƒ e”ƒ k” ’u`? | | | | ________ k“ƒ | | | | CCWOOR9 |

| 303J |  | H | uÑ<µ¨< U¡”Áƒ& Ÿ`e­ K?L Ÿ}KSÅ¨< SÅu— e^ ¾k\ e”ƒ c­‹ ’u\? | | | | [ ] [ ] ›ªm­‹ | | | | CCWONU9 |
| --- | --- | --- | --- | --- | --- | --- | --- | --- | --- | --- | --- |
|  |  |  |  |  |  |  | ›ªm 1 | [ ] [ ] k“ƒ | | | CCWOA19  CCWOA29  CCWOA39 |
|  |  |  |  |  |  |  | ›ªm 2 | [ ] [ ] k“ƒ | | |  |
|  |  |  |  |  |  |  | ›ªm 3 | [ ] [ ] k“ƒ | | |  |
|  |  | J | uÖpLL¨< ¨Ñ@h KT’ÒÑ` uH@Æuƒ ¨pƒ Ÿ¨Ñ@h¨< Ò` U” ÁIM Ñ>²? ›Öñ; | | | | ___________ c¯ƒ | | | | CCWODU9 |
|  |  | K | uÖpLL¨< KSÉH’>ƒ KU`S^“ KvKS<Á U” ÁIM w` ›Öñ; | | | | ___________ w` | | | | CCWOME9 |
| 303K | Ö”sÃ | | | ›­” | 1 | --$ 303M | | | | | CCTNQ9 |
|  |  |  |  | ¾KU | 0 |  |  |  |  |  |  |
|  |  | A | ¾Ö?“ ‹Ó\ U” ’u`;  (eS<” Ãéñƒ)? | | | |  | | | | CCTNQNA9 |
|  |  | B | vKñƒ 3 ¨^ƒ e”ƒ Ñ>²? ¿; | | | | ______ Ñ>²? | | | | CCTNQFR9 |
|  |  | C | Å`f SMe Ñ<µ¨< e”ƒ c¯ƒ ðËw­ƒ; | | | | _________ c¯ƒ | | | | CCTNQTR9 |
|  |  | D | KÑ<µ­ƒ e”ƒ w` ŸðK<; | | | | __________ w` | | | | CCTNQEX9 |
|  |  | E | Ö”sÃ KT’ÒÑ` uH@Æuƒ ¨pƒ KUÓw “ KS˜ Á¨Ö<ƒ ¨Ü U” Á¡M ’u`; | | | | __________ w` | | | | CCTNQAC9 |
|  |  | F | MÏ­ KI¡U“ c=H@É/eƒH@É T” ›wa H@Å; | | | | `e­ | | ›­” | 1 | CCTNQMO9 |
|  |  |  |  |  |  |  |  |  | ¾KU | 0 |  |
|  |  |  |  |  |  |  | vKu?ƒ­ | | ›­” | 1 | CCTNQHU9 |
|  |  |  |  |  |  |  |  |  | ¾KU | 0 |  |
|  |  |  |  |  |  |  | MÏ­ | | ›­” | 1 | CCTNQLI9 |
|  |  |  |  |  |  |  |  |  | ¾KU | 0 |  |
|  |  |  |  |  |  |  | K?L ¾u?}cw ›vM | | ›­” | 1 | CCTNQFA9 |
|  |  |  |  |  |  |  |  |  | ¾KU | 0 |  |
|  |  |  |  |  |  |  | Ô[u?ƒ | | ›­” | 1 | CCTNQNE9 |
|  |  |  |  |  |  |  |  |  | ¾KU | 0 |  |
|  |  |  |  |  |  |  | Ô[u?ƒ ÁMJ’ K?L ÕÅ— | | ›­” | 1 | CCTNQFR9 |
|  |  |  |  |  |  |  |  |  | ¾KU | 0 |  |
|  |  |  |  |  |  |  | ŸLÃ ÁM}Ökc K?L c¨< | | ›­” | 1 | CCYNQOT9 |
|  |  |  |  |  |  |  |  |  | ¾KU | 0 |  |
|  |  | G | u²=I Ñ<µ U¡”Áƒ Ÿ}KSÅ¨< SÅu— e^ ¾k\ƒ e”ƒ k” ’u`? | | | | __________k“ƒ | | | | CCYNQOR9 |
|  |  | H | uÑ<µ¨< U¡”Áƒ& Ÿ`e­ K?L Ÿ}KSÅ¨< SÅu— e^ ¾k\ e”ƒ c­‹ ’u\? | | | | [ ] [ ] ›ªm­‹ | | | | CCTNQNU9 |
|  |  |  |  |  |  |  | ›ªm 1 | [ ] [ ] k“ƒ | | | CCTNQA19  CCTNQA29  CCTNQA39 |
|  |  |  |  |  |  |  | ›ªm 2 | [ ] [ ] k“ƒ | | |  |
|  |  |  |  |  |  |  | ›ªm 3 | [ ] [ ] k“ƒ | | |  |
|  |  | J | uÖpLL¨< ŸÖ”s¿ Ò` U” ÁIM Ñ>²? ›Öñ; | | | | ___________ c¯ƒ | | | | CCTNQDU9 |
|  |  | K | uÖpLL¨< KSÉH’>ƒ KU`S^“ KvKS<Á U” ÁIM w` ›Öñ; | | | | ___________ w` | | | | CCTNQME9 |

| 303M | K?L ¾vIM I¡U“ ›ªm ¨ÃU ¾Ö?“ vKS<Á (ÃÑKî) | | | ›­” | 1 | --$ 304 | | | | | CCOTH9 |
| --- | --- | --- | --- | --- | --- | --- | --- | --- | --- | --- | --- |
|  |  |  |  | ¾KU | 0 |  |  |  |  |  |  |
|  |  | A | ¾Ö?“ ‹Ó\ U” ’u`;  (eS<” Ãéñƒ)? | | | |  | | | | CCOTHNA9 |
|  |  | B | vKñƒ 3 ¨^ƒ e”ƒ Ñ>²? ¿; | | | | ______ Ñ>²? | | | | CCOTHFR9 |
|  |  | C | Å`f SMe Ñ<µ¨< e”ƒ c¯ƒ ðËw­ƒ; | | | | _________ c¯ƒ | | | | CCOTHTR9 |
|  |  | D | KÑ<µ­ƒ e”ƒ w` ŸðK<; | | | | __________ w` | | | | CCOTHEX9 |
|  |  | E | ÃI” vKS<Á KTÓ–ƒ uH@Æuƒ ¨pƒ KUÓw “ KS˜ Á¨Ö<ƒ ¨Ü U” Á¡M ’u`; | | | | __________ w` | | | | CCOTHAC9 |
|  |  | F | MÏ­ KI¡U“ c=H@É/eƒH@É T” ›wa H@Å; | | | | `e­ | | ›­” | 1 | CCOTHMO9 |
|  |  |  |  |  |  |  |  |  | ¾KU | 0 |  |
|  |  |  |  |  |  |  | vKu?ƒ­ | | ›­” | 1 | CCOTHHU9 |
|  |  |  |  |  |  |  |  |  | ¾KU | 0 |  |
|  |  |  |  |  |  |  | MÏ­ | | ›­” | 1 | CCOTHLI9 |
|  |  |  |  |  |  |  |  |  | ¾KU | 0 |  |
|  |  |  |  |  |  |  | K?L ¾u?}cw ›vM | | ›­” | 1 | CCOTHFA9 |
|  |  |  |  |  |  |  |  |  | ¾KU | 0 |  |
|  |  |  |  |  |  |  | Ô[u?ƒ | | ›­” | 1 | CCOTHNE9 |
|  |  |  |  |  |  |  |  |  | ¾KU | 0 |  |
|  |  |  |  |  |  |  | Ô[u?ƒ ÁMJ’ K?L ÕÅ— | | ›­” | 1 | CCOTHFR9 |
|  |  |  |  |  |  |  |  |  | ¾KU | 0 |  |
|  |  |  |  |  |  |  | ŸLÃ ÁM}Ökc K?L c¨< | | ›­” | 1 | CCOTHOT9 |
|  |  |  |  |  |  |  |  |  | ¾KU | 0 |  |
|  |  | G | u²=I Ñ<µ U¡”Áƒ Ÿ}KSÅ¨< SÅu— e^ ¾k\ƒ e”ƒ k” ’u`? | | | | ________ k“ƒ | | | | CCOTHOR9 |
|  |  | H | uÑ<µ¨< U¡”Áƒ& Ÿ`e­ K?L Ÿ}KSÅ¨< SÅu— e^ ¾k\ e”ƒ c­‹ ’u\? | | | | [ ] [ ] ›ªm­‹ | | | | CCOTNU9 |
|  |  |  |  |  |  |  | ›ªm 1 | [ ] [ ] k“ƒ | | | CCOTHA19  CCOTHA29  CCOTHA39 |
|  |  |  |  |  |  |  | ›ªm 2 | [ ] [ ] k“ƒ | | |  |
|  |  |  |  |  |  |  | ›ªm 3 | [ ] [ ] k“ƒ | | |  |
|  |  | J | uÖpLL¨< Ÿ²=I vKS<Á Ò` U” ÁIM Ñ>²? ›Öñ; | | | | _____________ c¯ƒ | | | | CCOTHDU9 |
|  |  | K | uÖpLL¨< KSÉH’>ƒ KU`S^“ KvKS<Á U” ÁIM w` ›Öñ; | | | | ___________ w` | | | | CCOTHME9 |

**Information provided from medical records kept by Health Extension Workers**

| 001 | Pseudonymised identifier |  | |
| --- | --- | --- | --- |
| 1 | Timing of first attendance for antenatal care | ____ ____ weeks gestation | |
| 2 | Number of current ANC visit | 1^st^ visit | 1 |
|  |  | 2^nd^ visit | 2 |
|  |  | 3^rd^ visit | 3 |
|  |  | 4^th^ visit | 4 |
|  |  | 5^th^ or more visit | 5 |
|  |  | No documentation | 7 |
| 3 | Gravida | ____ ____ | |
| 4 | Parity | ____ ____ | |
| 5 | Gestation | ____ ____ weeks | |
| 6 | Any current problems | 1.  2.  3. | |
| 7 | Documentation of past history of mental health problems | Yes, documented history of mental disorder | 1 |
|  |  | Yes, documented that no history of mental disorder | 2 |
|  |  | No documentation | 7 |
| 8 | Documentation of current mental health problems | Yes | 1 |
|  |  | No | 0 |
|  |  | No documentation | 7 |
| 9 | Documentation of current violence exposure | Yes | 1 |
|  |  | No | 0 |
|  |  | No documentation | 7 |
| 10 | ANC appointment attendance | All attended | 1 |
|  |  | Some attended | 2 |
|  |  | None attended | 3 |
|  |  | No documentation | 5 |
| 11 | Medications prescribed | Physical health | 1 |
|  |  | Mental health | 2 |
|  |  | Both | 3 |
|  |  | None prescribed | 4 |
|  |  | No documentation | 7 |
| 12 | Obstetric outcome/complications | Haemorrhage | 1 |
|  |  | Obstructed/prolonged labour | 2 |
|  |  | Sepsis | 3 |
|  |  | Miscarriage | 4 |
|  |  | (Pre-)eclampsia | 5 |
|  |  | Ruptured uterus | 6 |
|  |  | No documentation | 7 |
| 13 | Neonatal outcome/complications | Live birth without complications of prematurity | 1 |
|  |  | Live birth with complications of prematurity | 2 |
|  |  | Stillbirth (late foetal death at/after 24 weeks at/before delivery) | 3 |
|  |  | Missed abortion (early foetal death before 24 weeks gestation with retention of foetus) | 4 |
|  |  | Early neonatal death | 5 |
|  |  | Spontaneous abortion | 6 |
|  |  | No documentation | 7 |

# Suplementary Files 5

## Date and version identifier

“Issue Date: 17 Dec, 2019
Protocol Amendment Number: 00
Author(s): PhD*.; TB*.

**Revision Chronology:**

| Feb 24, 2019 | Original  After a series of comments by supervisors and coauthors |
| --- | --- |
| Feb 28, 2019 | Initial submission to IRB |
| Aug 09, 2019 | IRB comments revised |
| July 10, 2019 | IRP Approved |
| Dec 17, 2019 | Submission to trials  Protocol written in a manuscript form following SPIRIT checklist |
| Feb 27, 2020 | Protocol revised and enrollment, intervention and assessment schedule prepared |

##

## Name and contact information for the trial sponsor (****Item 5b)****

**Trial Sponsor**: Addis Ababa University
**Sponsor’s Reference**:
**Contact name**: Dr Charlotte Hanlon
**Address**: College of Health Science, Department of Psychiatry
**Telephone**: +251912803374
**Email**: charlotte.hanlon@kcl.ac,uk

## Organizational structure and responsibilities

**Principal Investigator and investigators**

- Design and conduct of the trial
- Preparation of protocol and revisions
- Preparation of intervention manual and flip chart
- Organising steering committee meetings
- Publication of study reports]

**Steering committee (SC):** (members: All lead investigators on title page)

- Agreement of final protocol
- Recruitment of patients and liasing with PI
- Reviewing progress of study and if necessary agreeing changes to the protocol and/or training manual to facilitate the smooth running of the study.

**Trial Management Committee (TMC) (members:** PI, Administrator, trial coordinator)

- Study planning
- Organisation of steering committee meetings
- Provide annual risk report MHRA [Medicines and Healthcare products Regulatory Agency] and ethics committee
- Reporting SAE
- Responsible for trial master file
- Budget administration and contractual issues with individual centres
- Advice for lead investigators
- Audit of 6 monthly feedback forms and decide when site visit to occur.
- Assistance with international review, board/independent ethics committee applications
- Data verification
- Randomisation
- Organisation of central serum sample collection

**Data Manager**

Maintenance of trial IT system and data entry

- Data verification

Standard Protocol Items for Clinical Trials (SPIRIT) checklist ([81](#_ENREF_81)) was adhered to write this protocol manuscript report.

Supplementary file 6: theory of change map

1. Changed from statement into question format. [↑](#footnote-ref-1)
2. Response categories were simplified (initial yes/no, with further probing of the frequency in persons responding ‘yes’). [↑](#footnote-ref-2)
3. Reference period repeated for each item. [↑](#footnote-ref-3)
4. Broken into two parts. [↑](#footnote-ref-4)
